# Supplementary material for: Synthesis, Structure and Cytotoxicity Testing of Novel 7-(4,5-Dihydro-1H-imidazol-2-yl)-2-aryl-6,7-dihydro-2H-imidazo[2,1-c][1,2,4]triazol-3(5H)-Imine Derivatives
Source: Molecules. 2020 Dec 14;25(24):5924. doi: 10.3390/molecules25245924 (PMC7765142; doi:10.3390/molecules25245924)

Supplementary Materials

Synthesis, structure and cytotoxicity testing of novel 7-(4,5-dihydro-1*H*-imidazol-2-yl)-2-aryl-6,7-dihydro-2*H*-imidazo[2,1-*c*][1,2,4]triazol-3(5*H*)-imine derivatives

Łukasz Balewski[1,*], Franciszek Sączewski[1,†], Patrick J. Bednarski[2], Lisa Wolff[2], Anna Nadworska[1], Maria Gdaniec[3], and Anita Kornicka[1]

[1] Department of Chemical Technology of Drugs, Faculty of Pharmacy, Medical University of Gdańsk, Al. Gen. J. Hallera 107, 80-416 Gdańsk, Poland;

[2] Department of Pharmaceutical and Medicinal Chemistry, Institute of Pharmacy, University of Greifswald, F.-L. Jahn Strasse 17, D-17489 Greifswald, Germany;

[3] Faculty of Chemistry, Adam Mickiewicz University, ul. Uniwersytetu Poznańskiego 8, 61-614 Poznań, Poland;

[*] Author to whom correspondence should be addressed; E-Mail: lukasz.balewski@gumed.edu.pl

[†] Deceased 18 October 2018

Synthesis of compounds **1a-c** and **2**

1-Phenylhydrazinecarbonitrile (**1a**)

Cyanogen bromide (7.41 g; 70 mmol) was dissolved in 30 ml of ethanol and added dropwise with stirring over 15 min. into 180 ml of water at room temperature (20-22°C). Phenylhydrazine (7.57 g, 70 mmol) was dissolved in 10 ml of ethanol and added dropwise into the resulting mixture. The mixture was kept at ambient temperature for 1.5 h and cooled in an ice bath. The precipitate was filtered, washed with a small amount of water, and dried. The crude product was purified by column chromatography (eluent: dichloromethane or chloroform); yield 2.8 g (30%); m.p. 80-82°C; IR (KBr, cm^{-1}): 3360, 3283, 3039, 2925, 2278, 2204, 1623, 1597, 1496, 1458, 1310, 1214, 1175, 1147, 1063, 1023, 887, 750, 741, 688; m/z (ESI): 134 $[M+H]^+$. Anal. Calcd for $C_7H_7N_3$ (133.15): C, 63.14; H, 5.30; N, 31.56. Found: C, 63.09; H, 5.28; N, 31.48.

1-(p-Tolyl)hydrazinecarbonitrile (**1b**)

p-Tolylhydrazine hydrochloride (5.0 g; 31.52 mmol) was added into 100 ml of 5% NaOH at temperatures below 20-22°C. The resulting suspension was then extracted with diethyl ether (4x25 ml). The combined organic extract was dried with anhydrous magnesium sulfate(VI), filtered, and concentrated under reduced pressure. Cyanogen bromide (2.90 g; 27.3 mmol) was dissolved in 10 ml of ethanol and added dropwise with stirring over 15 min. into 100 ml of water at room temperature (20-22°C). Crude *p*-tolylhydrazine (3.34 g, 27.3 mmol) was dissolved in 15 ml of ethanol and added dropwise into the resulting mixture. The mixture was kept at ambient temperature for 3 h and cooled in an ice bath. The precipitate was filtered, washed with a small amount of water, and dried. The crude product was purified by column chromatography (eluent: dichloromethane); yield 1.2 g (30%); m.p. 71-73°C; IR (KBr, cm^{-1}): 3404, 3317, 3205, 3073, 2943, 2862, 1678, 1626, 1591, 1533, 1500, 1457, 1386, 1330, 1287, 1251, 1217, 1104, 1055, 1027, 992, 927 , 902, 872, 801, 759, 707, 697, 652, 610; m/z (ESI): 148 $[M+H]^+$. Anal. Calcd for $C_8H_9N_3$ (147.18): C, 65.29; H, 6.16; N, 28.55. Found: C, 65.34; H, 6.08; N, 28.49.

1-(4-Chlorophenyl)hydrazinecarbonitrile (**1c**)

(4-Chlorophenyl)hydrazine hydrochloride (5.0 g; 27.925 mmol) was added into 60-70 ml of 5% NaOH at temperatures below 20-22°C. The resulting suspension was then extracted with diethyl ether (4x20 ml). The combined organic extract was dried with anhydrous magnesium sulfate(VI), filtered, and concentrated under reduced pressure. Cyanogen bromide (2.82 g; 26.6 mmol) was dissolved in 20 ml of ethanol and added dropwise with stirring over 15 min. into 130 ml of water at room temperature (20-22°C). Crude (4-chlorophenyl)hydrazine (3.8 g; 26.65 mmol) was dissolved in 20 ml of ethanol and added dropwise into the solution of cyanogen bromide at room temperature (20-22°C). The mixture was kept at ambient temperature for 3 h and cooled in an acetone-ice bath (-10°C). The precipitate was filtered, washed with a small amount of water, and dried. The crude product quickly turned dark by air and was purified by column chromatography (eluent: dichloromethane); yield 1.6 g (36%); m.p. 74-79°C; IR (KBr, cm^{-1}): 3338, 3278, 3197, 3097, 3069, 2209, 1624, 1595, 1491, 1269, 1093, 819, 807, 555, 497; m/z (ESI): 168 $[M+H]^+$. Anal. Calcd for $C_7H_6ClN_3$ (167.60): C, 50.17; H, 3.61; N, 25.07. Found: C, 50.12; H, 3.65; N, 24.91.

2-Chloro-4,5-dihydro-1H-imidazole (solution in dichloromethane) (**2**)

2-Chloro-4,5-dihydro-1*H*-imidazole hydrogen sulfate(VI) (5.0 g, 25.0 mmol) was added portion-wise to 50 ml of a 5% sodium hydroxide solution at temperatures below 10°C. The resulting solution was then extracted with dichloromethane (4x20 ml). The combined organic extract was dried with anhydrous magnesium sulfate(VI), filtered, and concentrated to a volume of 25-30 ml (solution of 2.5 g, 25 mmol of 2-chloro-4,5-dihydro-1*H*-imidazole in dichloromethane).

Copies of ^{1}H-NMR and ^{13}C-NMR spectra

^{1}H-NMR ($CDCl_3$, 200 MHz) spectrum of 7-(4,5-dihydro-1*H*-imidazol-2-yl)-2-phenyl-6,7-dihydro-2*H*-imidazo[2,1-*c*][1,2,4]triazol-3(5*H*)-imine (**3a**)

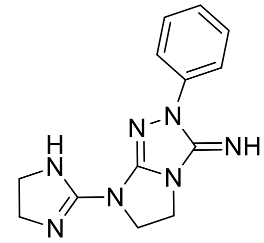

FREQ	PPM	INTENSITY	1009.45	5.048	25.935
1514.40	7.573	26.908	893.25	4.467	17.782
1506.34	7.533	47.871	886.00	4.431	34.469
1488.03	7.441	21.906	884.09	4.421	28.346
1480.42	7.403	39.916	877.86	4.390	32.950
1472.64	7.364	26.337	806.07	4.031	25.955
1467.88	7.340	18.315	797.65	3.989	35.711
1460.72	7.304	4.396	790.69	3.954	25.456
1452.41	7.263	10.568	783.65	3.919	7.883
1444.23	7.222	13.817	782.55	3.913	7.619
1436.83	7.185	20.399	776.40	3.882	13.959
1429.58	7.149	11.107	772.37	3.862	8.632
			732.45	3.663	167.711

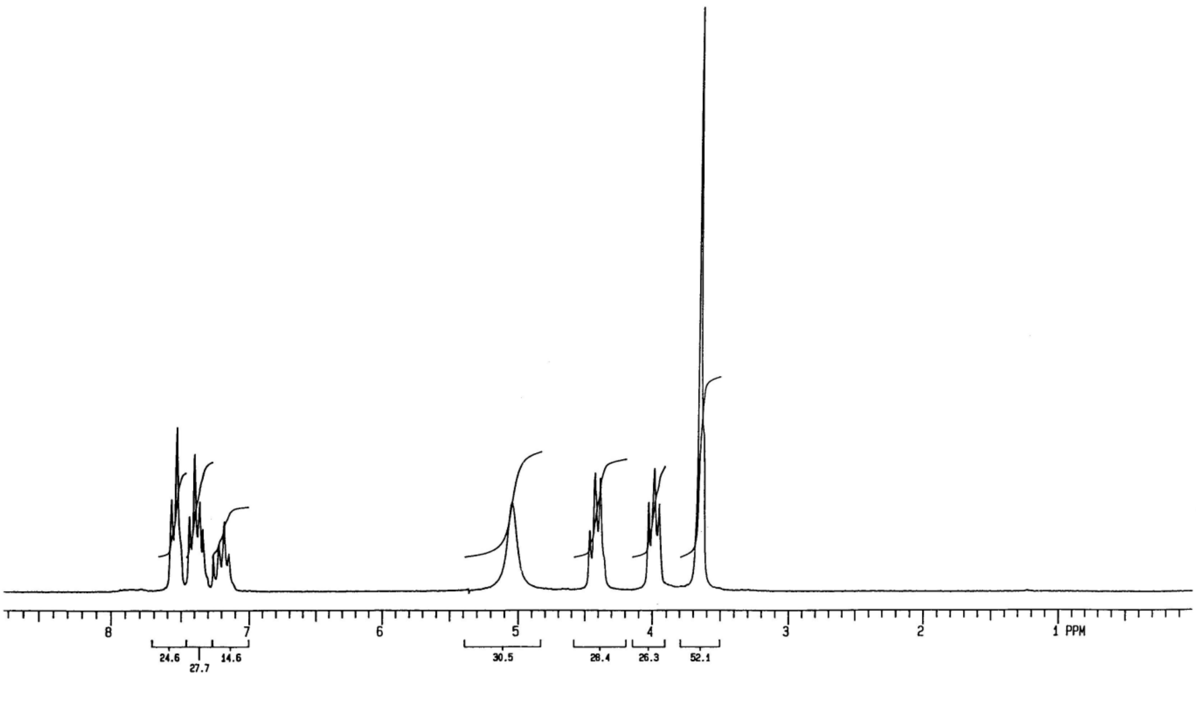

^{13}C-NMR ($CDCl_3$, 50 MHz) spectrum of 7-(4,5-dihydro-1*H*-imidazol-2-yl)-2-phenyl-6,7-dihydro-2*H*-imidazo[2,1-*c*][1,2,4]triazol-3(5*H*)-imine (**3a**)

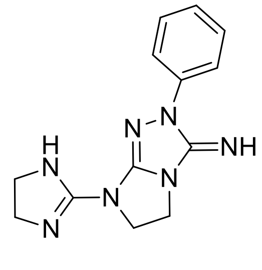

FREQ	PPM	INTENSITY
7820.6	155.512	17.212
7556.9	150.269	20.836
7535.3	149.838	11.998
7005.5	139.304	13.912
6523.9	129.726	131.262
6476.1	128.776	5.224
6322.3	125.719	68.909
6093.3	121.166	123.975
3932.4	78.196	43.240
3900.5	77.561	51.039
3868.5	76.924	49.754
2628.4	52.265	10.007
2566.6	51.036	63.431
2493.8	49.588	15.988
2305.0	45.835	10.347
2005.6	39.881	60.221

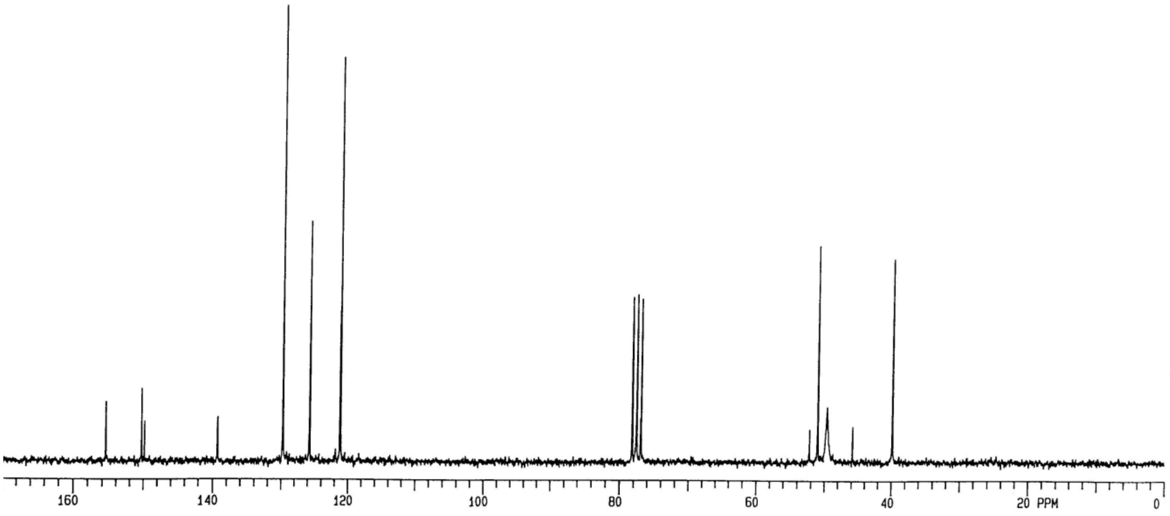

^{1}H-NMR (DMSO-d_6, 500 MHz) spectrum of 7-(4,5-dihydro-1*H*-imidazol-2-yl)-2-(*p*-tolyl)-6,7-dihydro-2*H*-imidazo[2,1-*c*][1,2,4]triazol-3(5*H*)-imine (**3b**)

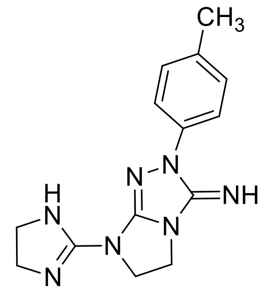

FREQUENCY	PPM	HEIGHT
3930.129	7.863	74.0
3921.339	7.846	75.6
3571.220	7.145	80.2
3562.431	7.128	75.4
2124.843	4.251	46.3
2118.007	4.238	65.9
2109.217	4.220	52.6
1931.472	3.864	53.1
1923.659	3.849	65.6
1916.334	3.834	44.2
1741.519	3.484	390.5
1244.907	2.491	11.3
1243.442	2.488	14.0
1128.200	2.257	275.9

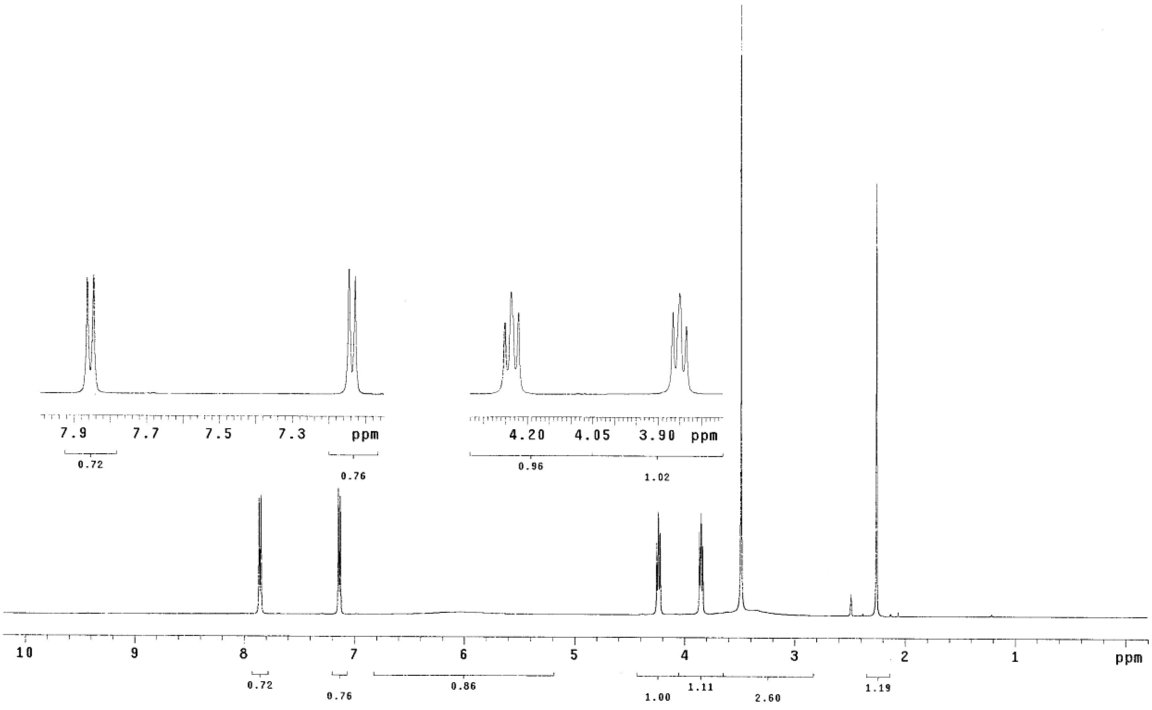

^{13}C-NMR (DMSO-d_6+TFA, 125 MHz) spectrum of 7-(4,5-dihydro-1*H*-imidazol-2-yl)-2-(*p*-tolyl)-6,7-dihydro-2*H*-imidazo[2,1-*c*][1,2,4]triazol-3(5*H*)-imine (**3b**)

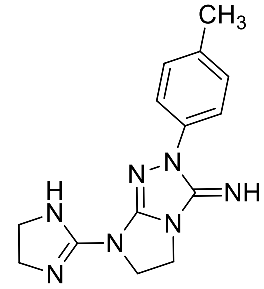

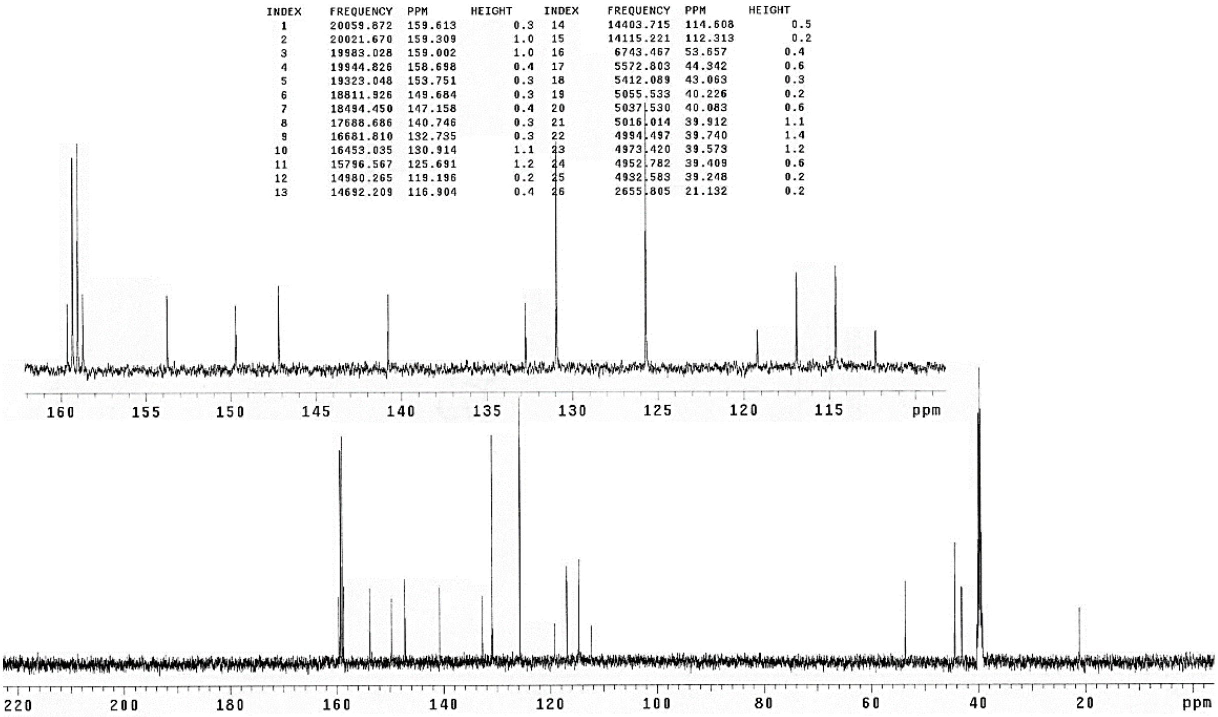

INDEX	FREQUENCY	PPM	HEIGHT	INDEX	FREQUENCY	PPM	HEIGHT
1	20059.872	159.613	0.3	14	14403.715	114.608	0.5
2	20021.670	159.309	1.0	15	14115.221	112.313	0.2
3	19983.028	159.002	1.0	16	6743.467	53.657	0.4
4	19944.826	158.698	0.4	17	5572.803	44.342	0.6
5	19323.048	153.751	0.3	18	5412.089	43.063	0.3
6	18811.926	149.684	0.3	19	5055.533	40.226	0.2
7	18494.450	147.158	0.4	20	5037.530	40.083	0.6
8	17688.686	140.746	0.3	21	5016.014	39.912	1.1
9	16681.810	132.735	0.3	22	4994.497	39.740	1.4
10	16453.035	130.914	1.1	23	4973.420	39.573	1.2
11	15796.567	125.691	1.2	24	4952.782	39.409	0.6
12	14980.265	119.196	0.2	25	4932.583	39.248	0.2
13	14692.209	116.904	0.4	26	2655.805	21.132	0.2

^{1}H-NMR (DMSO-d_6, 400 MHz) spectrum of 2-(4-chlorophenyl)-7-(4,5-dihydro-1*H*-imidazol-2-yl)-6,7-dihydro-2*H*-imidazo[2,1-*c*][1,2,4]triazol-3(5*H*)-imine (**3c**)

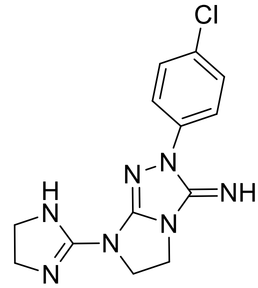

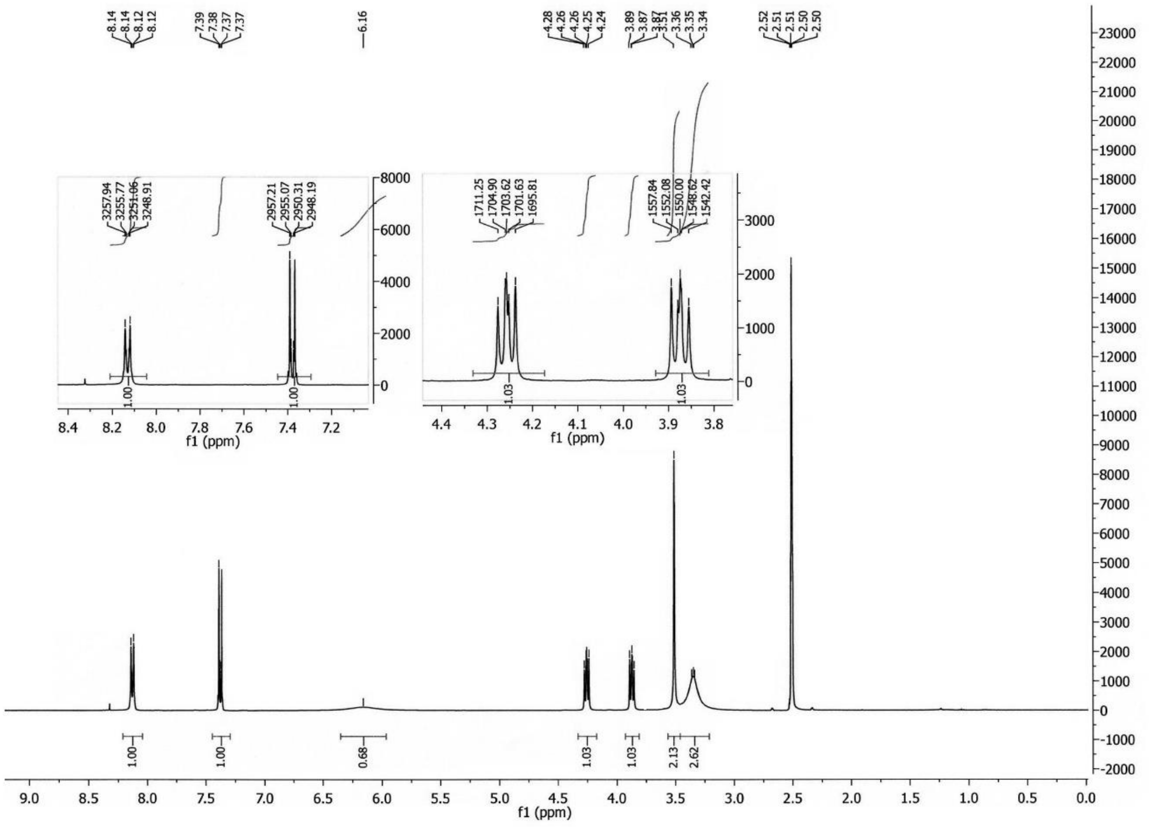

^{13}C-NMR (DMSO-d_6, 100 MHz) spectrum of 2-(4-chlorophenyl)-7-(4,5-dihydro-1*H*-imidazol-2-yl)-6,7-dihydro-2*H*-imidazo[2,1-*c*][1,2,4]triazol-3(5*H*)-imine (**3c**)

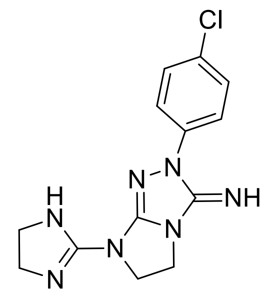

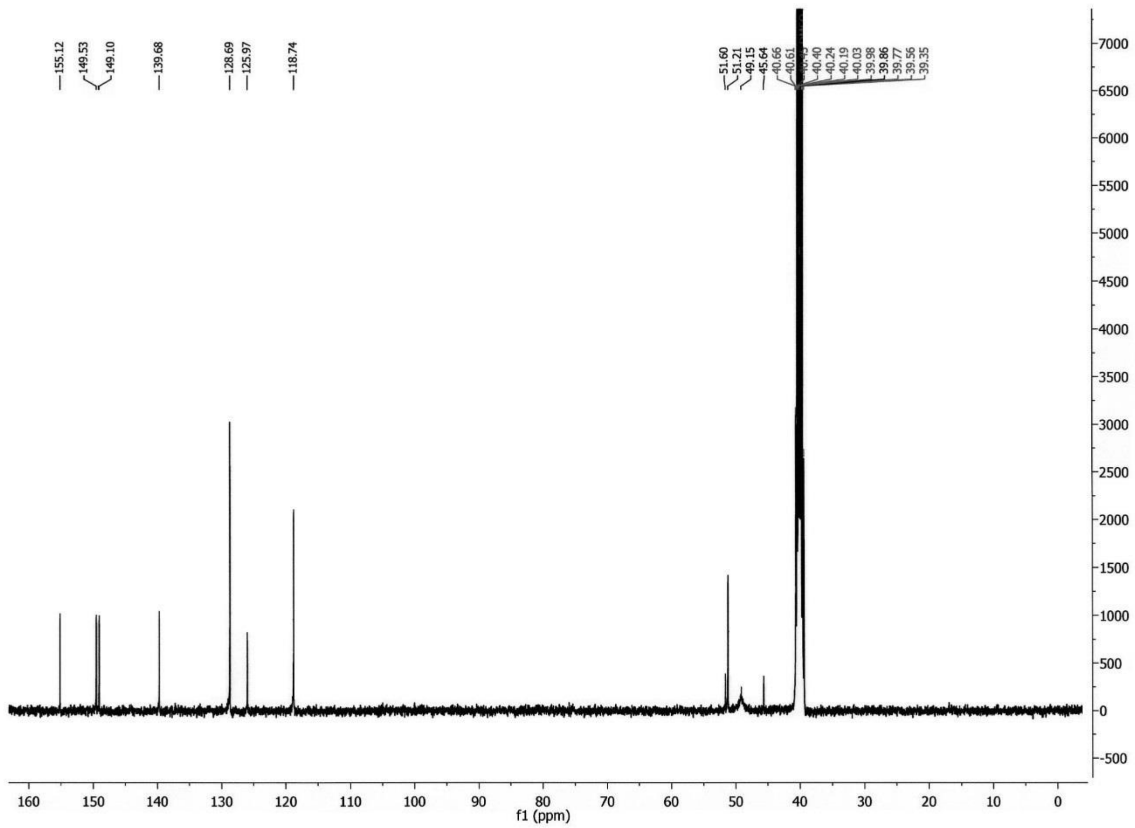

^{1}H-NMR ($CDCl_3$, 200 MHz) spectrum of *N*-(7-(4,5-dihydro-1*H*-imidazol-2-yl)-2-phenyl-6,7-dihydro-2*H*-imidazo[2,1-*c*][1,2,4]triazol-3(5*H*)-ylidene)benzamide (**4a**)

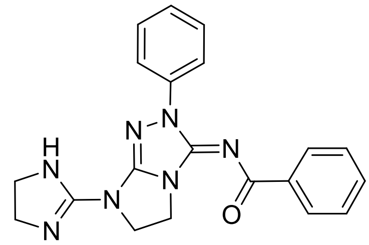

FREQ	PPM	INTENSITY
1642.93	8.216	58.082
1635.79	8.180	62.546
1607.43	8.038	57.798
1599.55	7.999	65.773
1497.34	7.488	56.888
1490.04	7.451	143.897
1482.26	7.412	94.244
1472.00	7.361	15.240
1470.48	7.353	8.638
1469.39	7.348	7.956
1463.23	7.317	27.737
1455.06	7.276	35.385
1450.01	7.251	14.752
1117.92	5.590	7.885
1114.95	5.575	8.082
1113.51	5.568	8.106
1111.88	5.560	8.179
1109.90	5.550	7.958
909.35	4.547	47.121
903.42	4.510	67.102
895.76	4.479	66.511
890.09	4.451	45.683
745.63	3.727	45.700
743.93	3.720	45.465

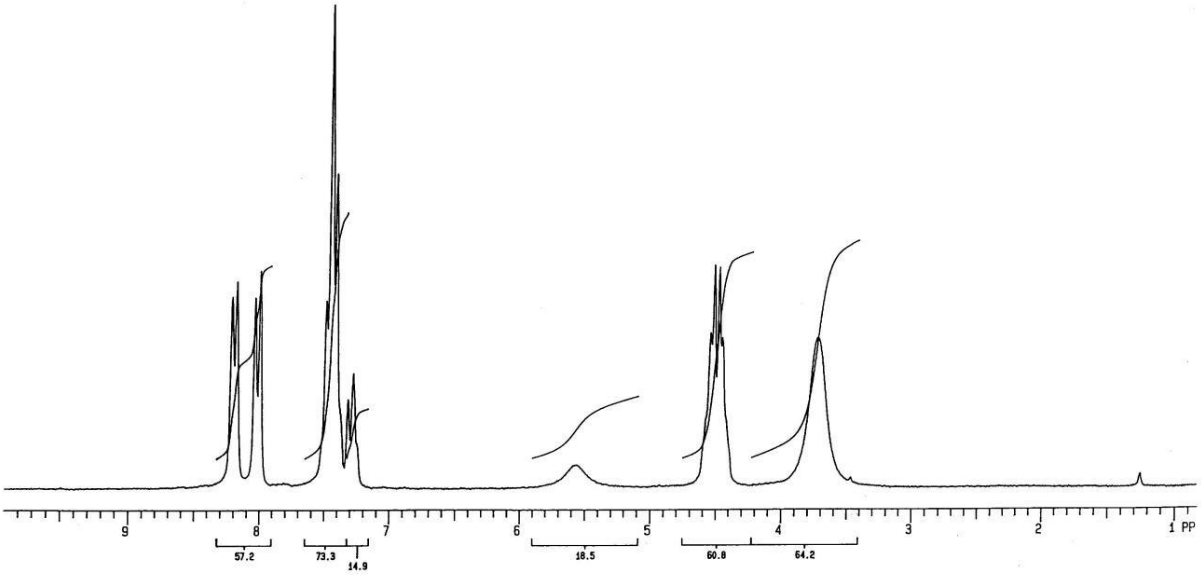

^{1}H-NMR ($CDCl_3$, 200 MHz) spectrum of *N*-(7-(4,5-dihydro-1*H*-imidazol-2-yl)-2-(*p*-tolyl)-6,7-dihydro-2*H*-imidazo[2,1-*c*][1,2,4]triazol-3(5*H*)-ylidene)-4-methylbenzamide (**4b**)

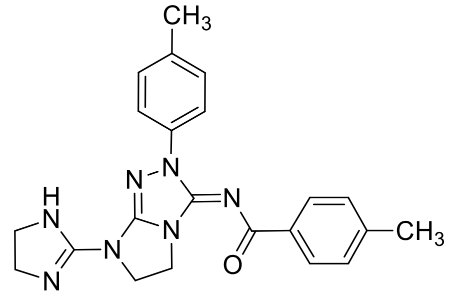

FREQ	PPM	INTENSITY
1619.15	8.097	24.459
1611.27	8.057	26.243
1576.18	7.882	23.565
1567.88	7.840	26.152
1451.37	7.258	33.995
1444.64	7.224	43.972
1437.73	7.190	28.693
907.43	4.538	21.059
902.33	4.512	29.130
895.09	4.476	28.891
889.80	4.450	19.988
744.80	3.724	62.446
478.13	2.391	124.946

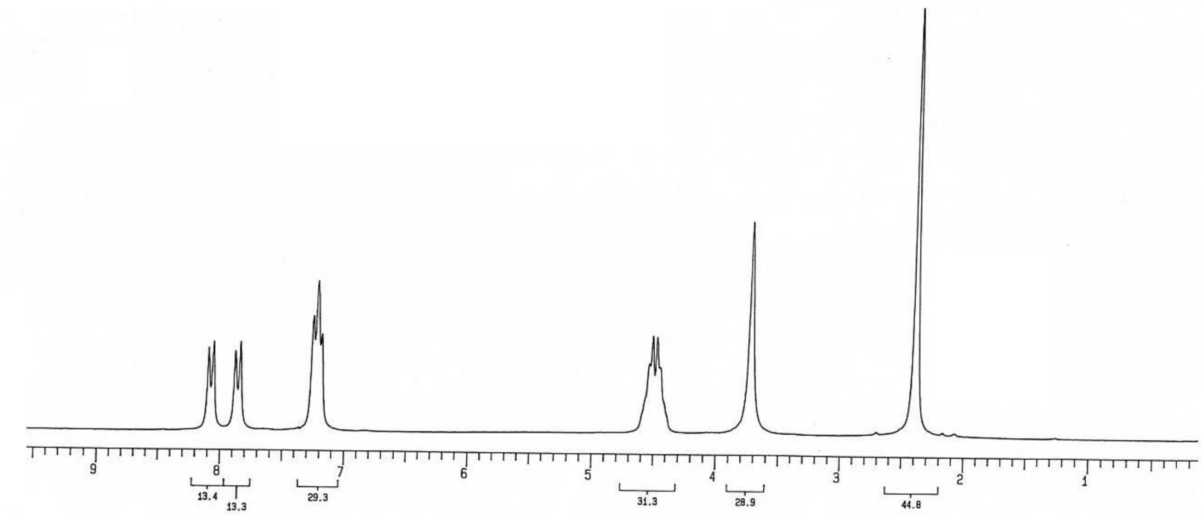

^{1}H-NMR ($CDCl_3$+TFA, 200 MHz) spectrum of *N*-(7-(4,5-dihydro-1*H*-imidazol-2-yl)-2-(*p*-tolyl)-6,7-dihydro-2*H*-imidazo[2,1-*c*][1,2,4]triazol-3(5*H*)-ylidene)-4-methylbenzamide (**4b**)

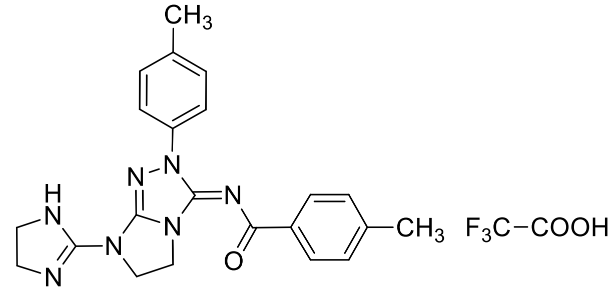

FREQ	PPM	INTENSITY
1575.72	7.880	9.129
1544.45	7.723	17.559
1536.56	7.684	19.606
1473.74	7.370	74.883
1462.84	7.315	21.158
1454.72	7.275	18.848
987.01	4.936	25.813
816.44	4.083	58.511
485.14	2.426	96.574

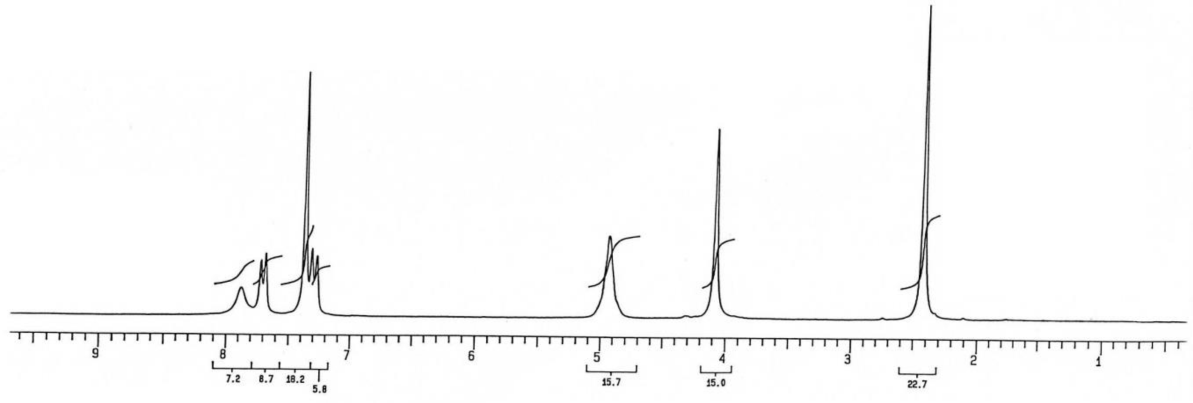

^{1}H-NMR ($CDCl_3$, 500 MHz) spectrum of *N*-(7-(4,5-dihydro-1*H*-imidazol-2-yl)-2-phenyl-6,7-dihydro-2*H*-imidazo[2,1-*c*][1,2,4]triazol-3(5*H*)-ylidene)-4-fluorobenzamide (**4c**)

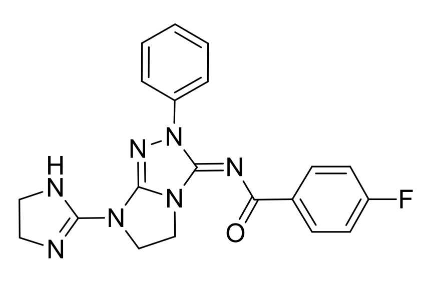

INDEX	FREQUENCY	PPM	HEIGHT	INDEX	FREQUENCY	PPM	HEIGHT
1	4095.657	8.194	24.3	17	2286.953	4.576	14.2
2	4090.286	8.184	29.6	18	2285.000	4.572	14.3
3	4087.356	8.178	27.4	19	2278.163	4.558	34.4
4	4081.496	8.166	23.0	20	2276.210	4.554	25.0
5	3986.764	7.977	37.0	21	2270.350	4.542	41.9
6	3978.951	7.961	38.8	22	2256.678	4.515	5.0
7	3732.354	7.468	24.6	23	2249.841	4.501	39.8
8	3725.029	7.453	42.5	24	2242.028	4.486	32.5
9	3716.728	7.436	25.6	25	2235.192	4.472	13.3
10	3653.247	7.309	13.0	26	2233.239	4.468	12.1
11	3645.923	7.295	21.3	27	1868.470	3.738	72.8
12	3638.598	7.280	9.5				
13	3630.785	7.264	23.2				
14	3538.982	7.081	26.2				
15	3530.193	7.063	48.2				
16	3521.403	7.045	23.1				

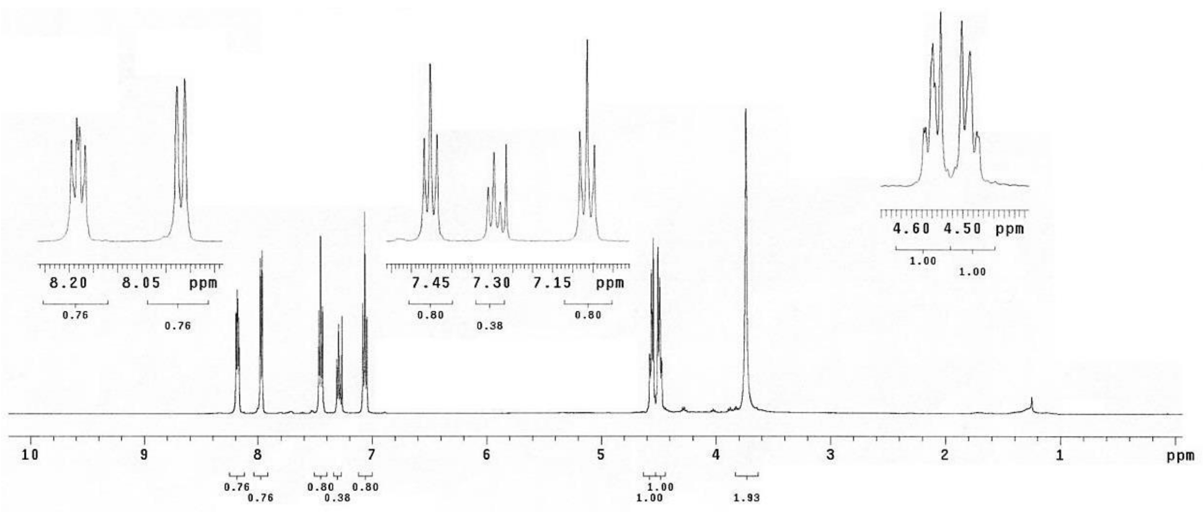

^{13}C-NMR ($CDCl_3$, 125 MHz) spectrum of *N*-(7-(4,5-dihydro-1*H*-imidazol-2-yl)-2-phenyl-6,7-dihydro-2*H*-imidazo[2,1-*c*][1,2,4]triazol-3(5*H*)-ylidene)-4-fluorobenzamide (**4c**)

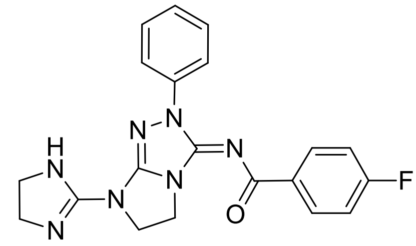

INDEX	FREQUENCY	PPM	HEIGHT	INDEX	FREQUENCY	PPM	HEIGHT
1	21565.541	171.595	0.4	21	5726.896	45.568	1.3
2	20888.875	166.210	0.3				
3	20638.144	164.215	0.2				
4	19460.894	154.848	0.6				
5	18922.986	150.568	0.5				
6	18646.347	148.367	0.4				
7	17358.881	138.123	0.5				
8	16795.944	133.643	0.3				
9	16792.870	133.619	0.3				
10	16601.419	132.096	1.5				
11	16592.197	132.022	1.4				
12	16213.247	129.007	3.3				
13	15941.439	126.844	1.5				
14	15333.273	122.005	3.5				
15	14475.694	115.182	1.5				
16	14454.178	115.010	1.6				
17	9745.178	77.541	1.9				
18	9713.562	77.290	2.0				
19	9681.507	77.035	2.0				
20	6361.847	50.621	1.2				

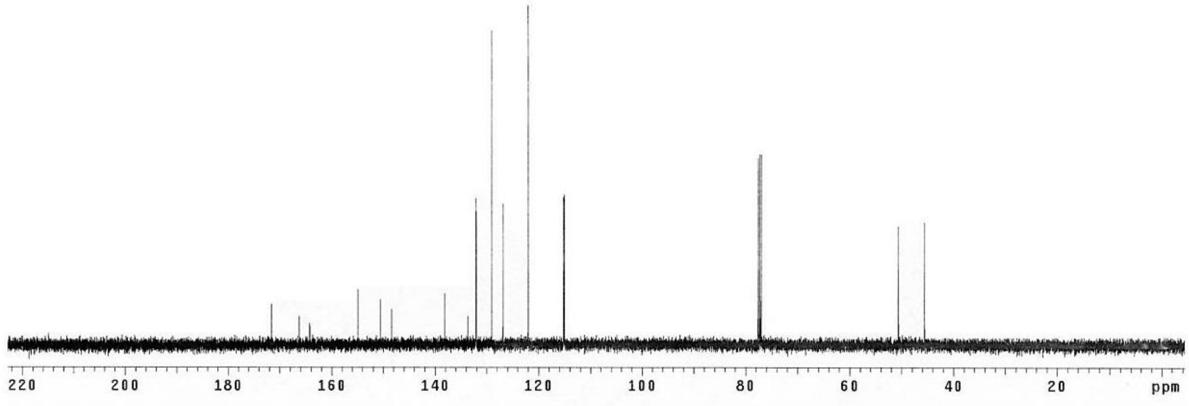

^{1}H-NMR ($CDCl_3$, 200 MHz) spectrum of 4-chloro-*N*-(7-(4,5-dihydro-1*H*-imidazol-2-yl)-2-phenyl-6,7-dihydro-2*H*-imidazo[2,1-*c*][1,2,4]triazol-3(5*H*)-ylidene)benzamide (**4d**)

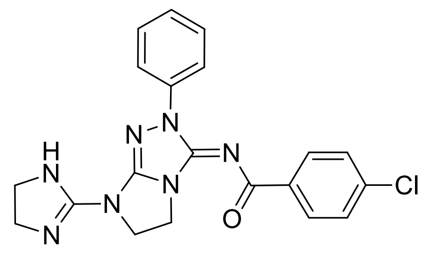

FREQ	PPM	INTENSITY			
1625.81	8.130	107.112	1109.03	5.546	14.794
1617.28	8.087	113.762	920.27	4.602	14.326
1596.77	7.985	80.376	916.73	4.584	20.281
1589.13	7.947	96.787	914.63	4.574	21.811
1497.06	7.486	42.841	910.32	4.552	53.002
1489.88	7.450	93.800	908.27	4.542	48.270
1481.67	7.409	65.922	903.60	4.519	87.382
1476.17	7.382	119.739	896.16	4.481	88.706
1467.57	7.339	109.294	889.23	4.447	51.929
1465.71	7.329	53.144	884.56	4.423	22.337
1457.56	7.289	47.908	882.77	4.414	20.254
1452.63	7.264	45.428	878.98	4.395	14.264
1450.13	7.252	17.646	756.07	3.781	33.778
1110.25	5.552	14.912	733.50	3.668	34.032

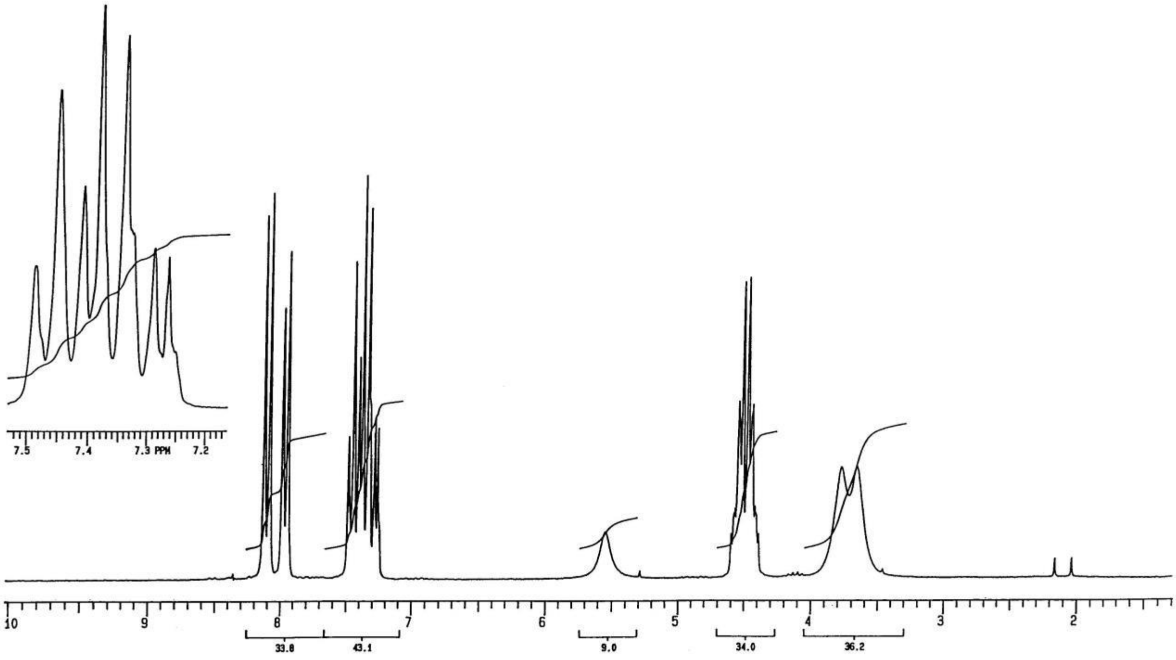

^{1}H-NMR (DMSO-d_6, 400 MHz) spectrum of 4-chloro-*N*-(2-(4-chlorophenyl)-7-(4,5-dihydro-1*H*-imidazol-2-yl)-6,7-dihydro-2*H*-imidazo[2,1-*c*][1,2,4]triazol-3(5*H*)-ylidene)benzamide (**4e**)

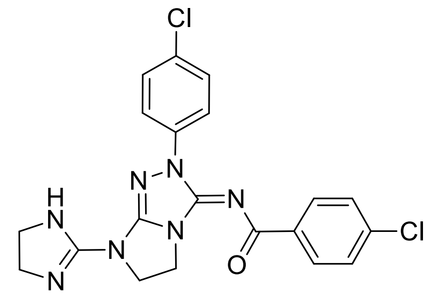

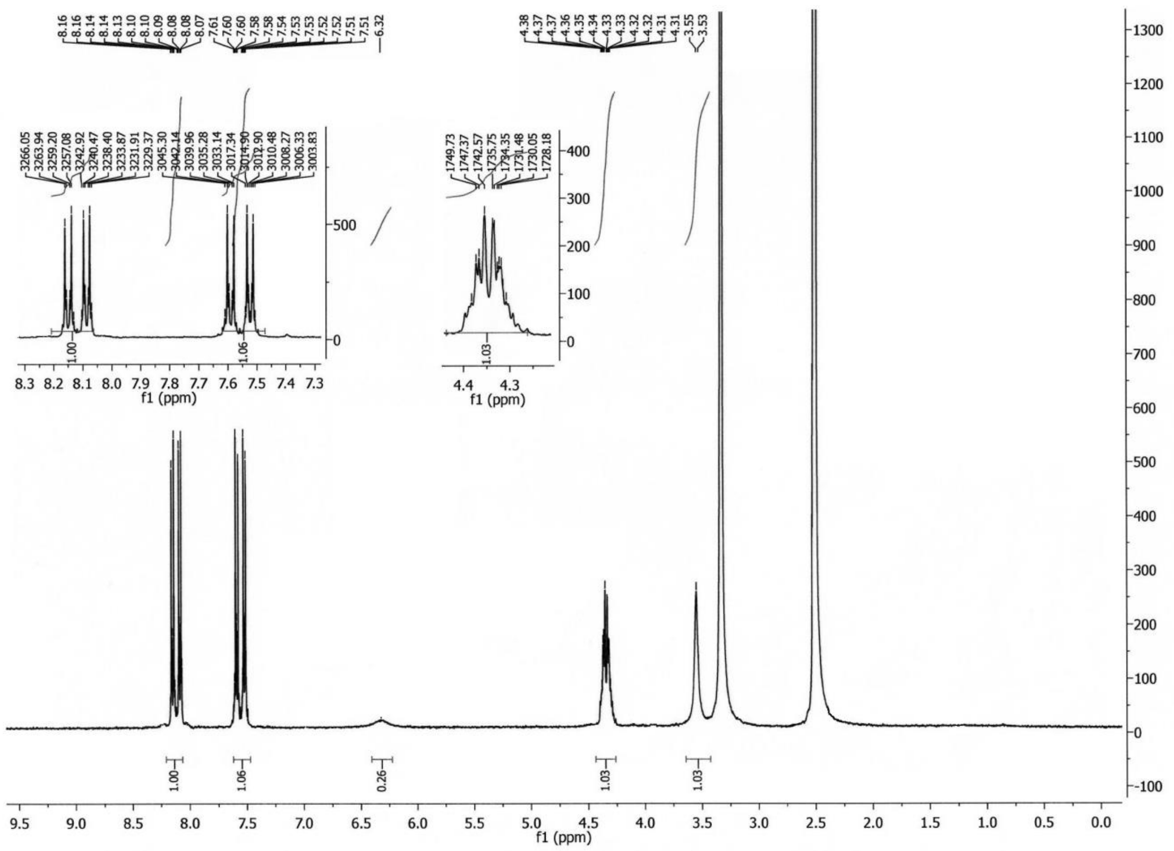

^{1}H-NMR ($CDCl_3$, 200 MHz) spectrum of *N*-(7-(4,5-dihydro-1*H*-imidazol-2-yl)-2-phenyl-6,7-dihydro-2*H*-imidazo[2,1-*c*][1,2,4]triazol-3(5*H*)-ylidene)methanesulfonamide (**5a**)

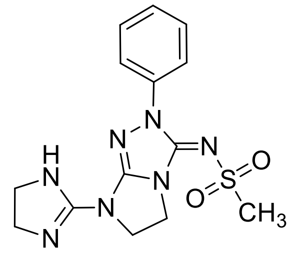

FREQ	PPM	INTENSITY
1560.97	7.806	31.003
1553.27	7.767	34.074
1490.34	7.453	20.033
1483.02	7.416	35.603
1475.13	7.377	23.243
1460.41	7.303	16.917
1453.09	7.266	25.682
1446.37	7.233	6.945
939.45	4.698	18.080
932.62	4.664	33.637
924.37	4.622	30.271
897.92	4.490	32.668
889.80	4.450	31.630
882.97	4.415	14.812
747.58	3.738	23.930
742.38	3.712	23.495
740.16	3.701	23.349
737.84	3.690	23.414
736.81	3.685	23.340
619.93	3.100	124.896

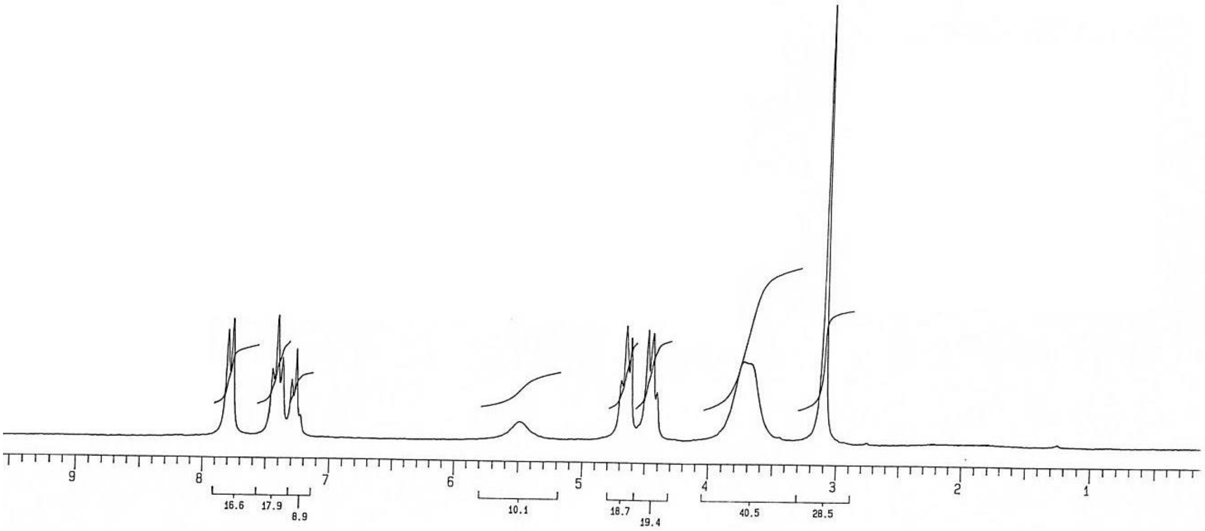

^{13}C-NMR ($CDCl_3$, 50 MHz) spectrum of *N*-(7-(4,5-dihydro-1*H*-imidazol-2-yl)-2-phenyl-6,7-dihydro-2*H*-imidazo[2,1-*c*][1,2,4]triazol-3(5*H*)-ylidene)methanesulfonamide (**5a**)

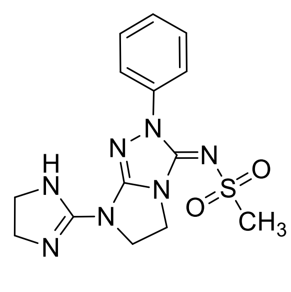

FREQ	PPM	INTENSIT
7791.2	154.928	16.648
7565.8	150.445	9.537
7258.8	144.341	6.786
6937.5	137.952	11.666
6500.3	129.257	104.031
6393.3	127.130	54.004
6137.6	122.045	107.439
3930.5	78.158	100.031
3908.3	77.717	8.142
3898.5	77.521	102.176
3866.5	76.886	99.845
2556.4	50.834	50.190
2300.6	45.746	52.945
2184.2	43.433	52.033

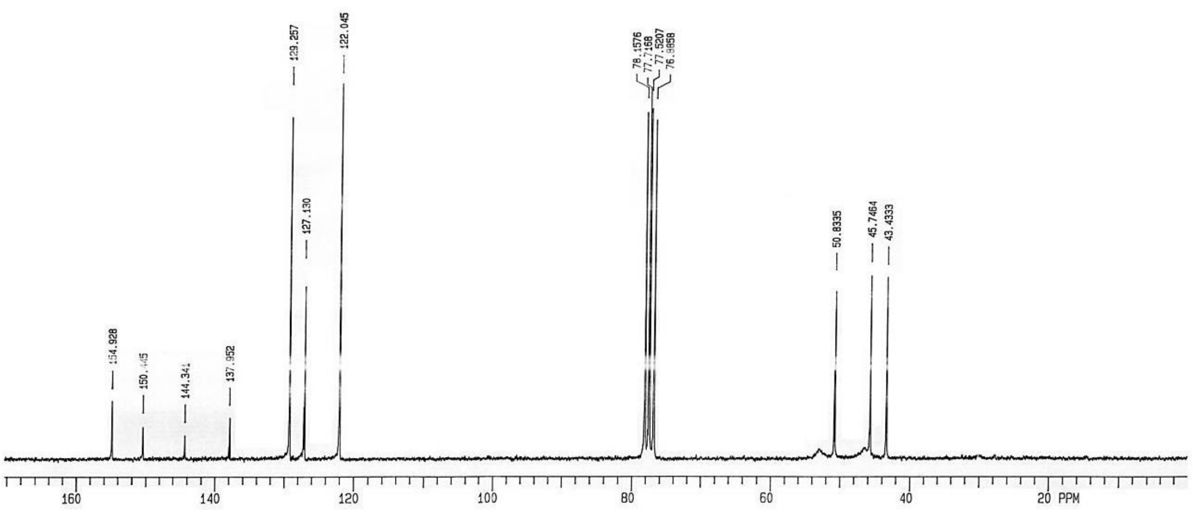

^{1}H-NMR ($CDCl_3$, 200 MHz) spectrum of *N*-(7-(4,5-dihydro-1*H*-imidazol-2-yl)-2-(*p*-tolyl)-6,7-dihydro-2*H*-imidazo[2,1-*c*][1,2,4]triazol-3(5*H*)-ylidene)methanesulfonamide (**5b**)

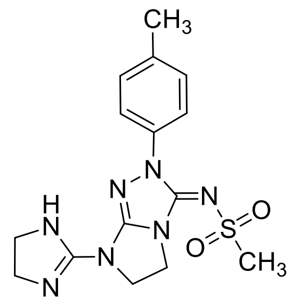

FRFQ	PPM	TNTFNSTTY
1530.53	7.654	34.360
1577.18	7.617	43.967
1453.77	7.767	17.801
1446.31	7.737	47.358
1438.75	7.197	36.897
938.88	4.695	14.606
931.97	4.660	33.617
973.85	4.670	34.748
897.87	4.490	31.983
889.70	4.449	34.594
887.84	4.415	17.003
740.99	3.705	37.815
616.69	3.084	148.319
474.01	7.370	178.705

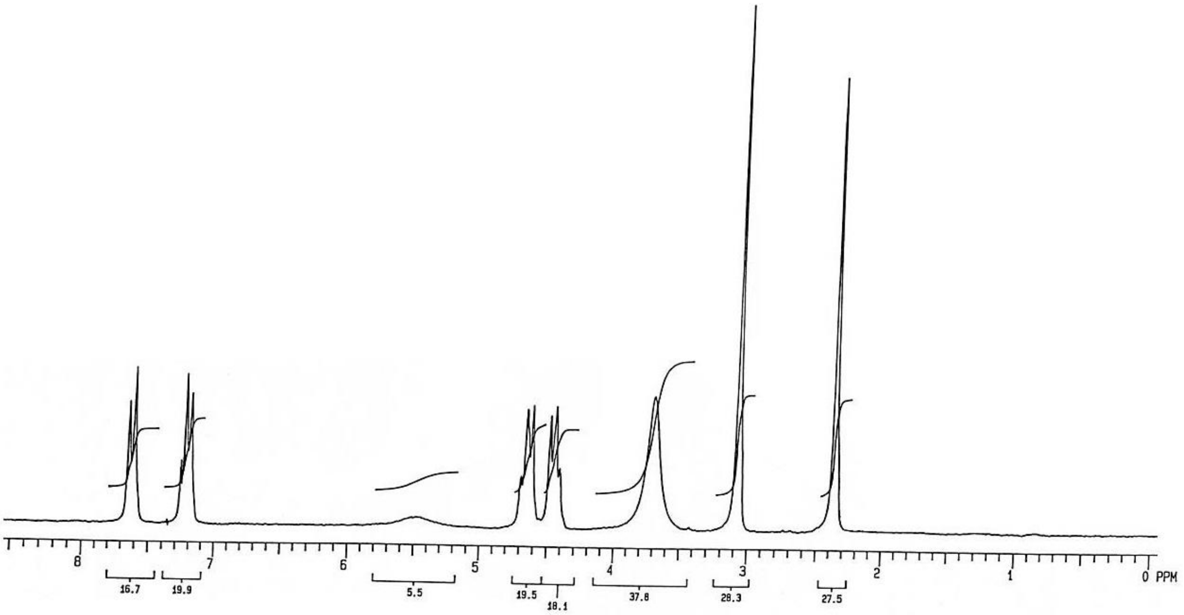

^{13}C-NMR ($CDCl_3$, 50 MHz) spectrum of *N*-(7-(4,5-dihydro-1*H*-imidazol-2-yl)-2-(*p*-tolyl)-6,7-dihydro-2*H*-imidazo[2,1-*c*][1,2,4]triazol-3(5*H*)-ylidene)methanesulfonamide (**5b**)

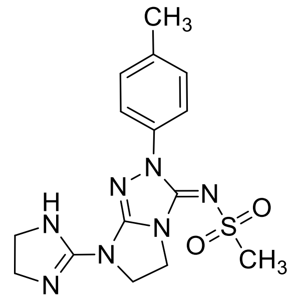

FRFQ	PPM	TNTFNSTTY
7793.6	154.974	18.395
7561.4	150.358	13.800
7754.6	144.758	8.117
6901.3	137.737	70.841
6811.7	135.440	13.474
6578.4	179.817	179.337
6146.8	177.779	130.369
3930.6	78.160	95.105
3898.7	77.575	98.330
3866.7	76.890	95.306
7555.3	50.811	56.598
7798.8	45.717	60.056
7183.3	43.415	64.845
1087.4	71.574	45.731

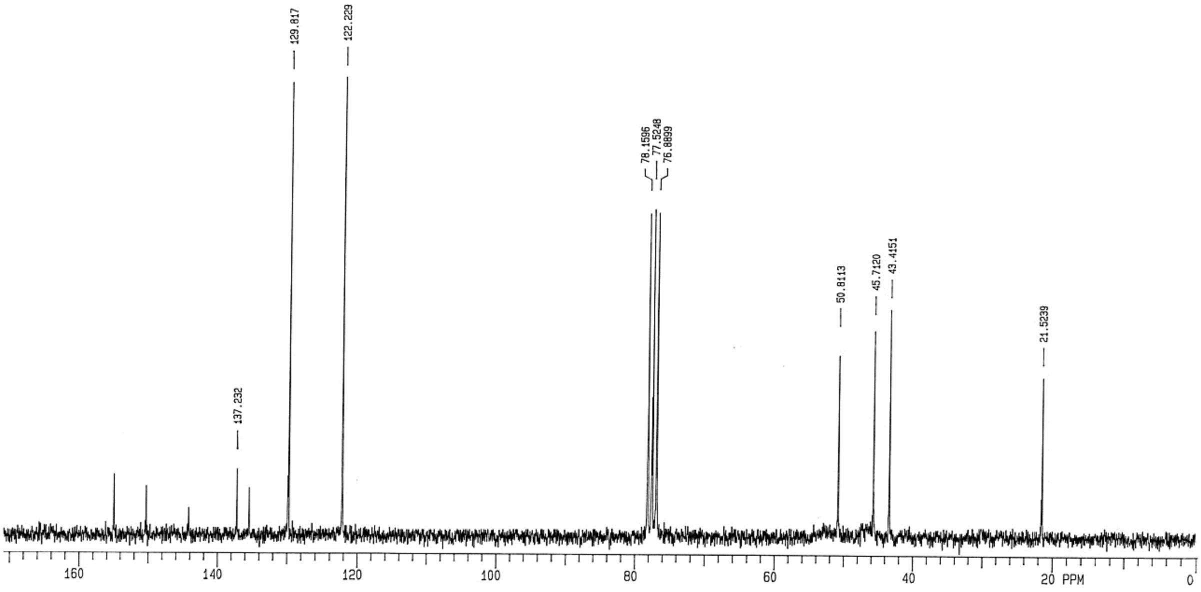

^{1}H-NMR ($CDCl_3$, 200 MHz) spectrum of *N*-(7-(4,5-dihydro-1*H*-imidazol-2-yl)-2-phenyl-6,7-dihydro-2*H*-imidazo[2,1-*c*][1,2,4]triazol-3(5*H*)-ylidene)benzenesulfonamide (**5c**)

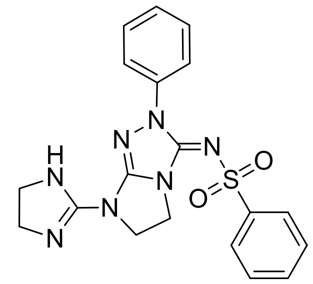

FREQ	PPM	INTENSITY
1597.46	7.988	50.973
1595.89	7.980	60.056
1594.49	7.973	41.931
1590.09	7.951	59.999
1588.00	7.941	63.052
1553.43	7.768	68.848
1545.54	7.729	76.365
1513.81	7.570	7.565
1512.08	7.561	7.650
1505.69	7.529	27.598
1500.48	7.503	122.871
1493.11	7.466	62.657
1487.54	7.439	18.339
1484.29	7.422	13.642
1478.08	7.391	35.066
1476.36	7.383	21.949
1471.02	7.356	76.325
1462.95	7.316	53.712
1452.97	7.266	71.201
1445.09	7.226	38.039
1437.66	7.189	12.021
1101.44	5.508	11.039
1099.82	5.500	10.923
1087.52	5.438	4.586
950.70	4.754	27.424
949.82	4.750	27.334
942.81	4.715	61.055
934.46	4.673	54.751
925.59	4.629	4.243
924.76	4.624	4.159
899.43	4.498	54.130
891.07	4.456	58.683
883.19	4.416	25.605
781.16	3.906	6.384
753.32	3.767	27.705
743.01	3.715	19.152
739.06	3.696	19.265
728.39	3.642	27.906
708.49	3.543	7.367
702.74	3.514	4.923
701.01	3.506	4.593
672.35	3.362	4.286
520.16	2.601	17.234
365.08	1.826	4.142
358.69	1.794	4.616
355.80	1.779	4.565
353.58	1.768	4.463
350.23	1.751	3.964

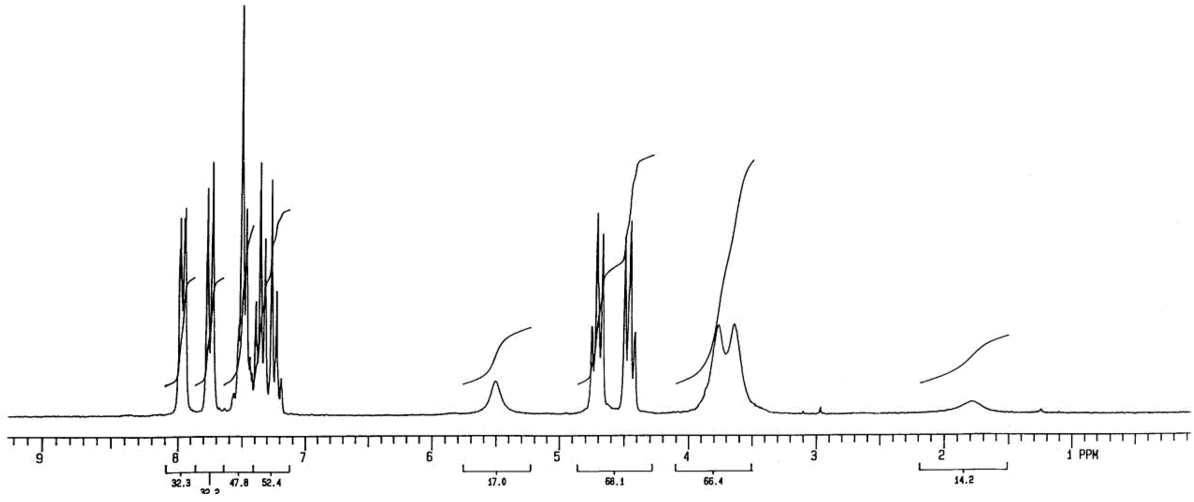

^{13}C-NMR ($CDCl_3$, 50 MHz) spectrum of *N*-(7-(4,5-dihydro-1*H*-imidazol-2-yl)-2-phenyl-6,7-dihydro-2*H*-imidazo[2,1-*c*][1,2,4]triazol-3(5*H*)-ylidene)benzenesulfonamide (**5c**)

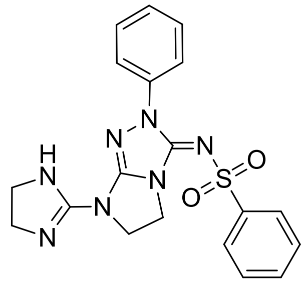

FREQ	PPM	INTENSITY
7790.6	154.915	14.353
7571.2	150.552	9.080
7257.5	144.315	10.259
7224.5	143.659	10.327
6933.6	137.875	16.365
6653.8	132.310	74.102
6500.0	129.251	158.255
6496.0	129.172	125.135
6394.5	127.155	69.514
6366.1	126.588	121.690
6133.9	121.972	142.004
3931.9	78.186	84.686
3926.1	78.071	8.720
3910.1	77.751	93.245
3900.0	77.551	89.950
3868.0	76.914	84.374
2668.0	53.054	54.504
2556.5	50.836	56.739
2333.3	46.397	11.388
2329.6	46.325	57.790
2304.9	45.833	57.871

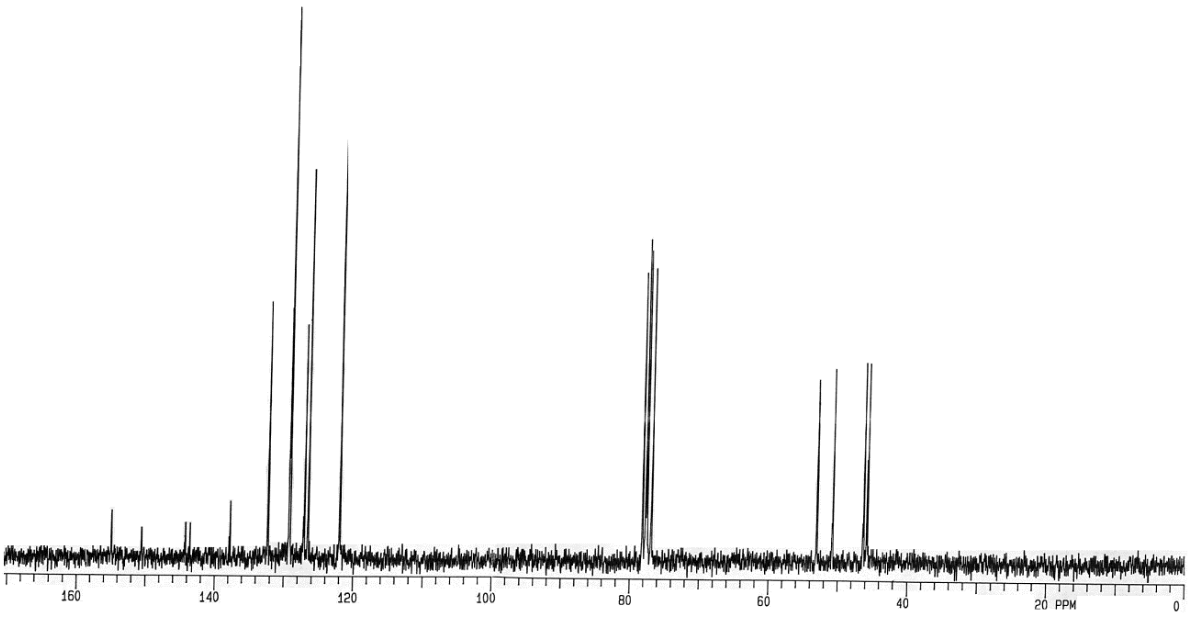

^{1}H-NMR ($CDCl_3$, 200 MHz) spectrum of *N*-(7-(4,5-dihydro-1*H*-imidazol-2-yl)-2-(*p*-tolyl)-6,7-dihydro-2*H*-imidazo[2,1-*c*][1,2,4]triazol-3(5*H*)-ylidene)benzenesulfonamide (**5d**)

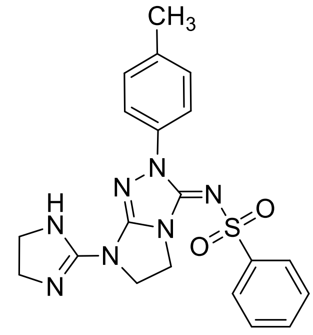

FREQ	PPM	INTENSITY			
1594.56	7.974	39.562	949.61	4.749	23.066
1588.81	7.945	41.702	942.18	4.711	50.287
1587.55	7.939	43.014	933.60	4.669	46.514
1524.73	7.625	54.645	898.80	4.495	46.096
1516.38	7.583	66.411	890.21	4.452	50.260
1499.21	7.497	86.682	883.02	4.416	22.508
1492.30	7.462	47.109	761.69	3.809	24.123
1486.86	7.435	12.710	754.49	3.773	27.507
1486.17	7.432	12.974	745.45	3.728	8.283
1483.95	7.421	8.215	726.37	3.632	27.032
1482.92	7.416	7.718	722.89	3.615	23.518
1452.58	7.264	19.368	720.80	3.604	23.612
1433.97	7.171	63.889	719.98	3.600	23.700
1425.67	7.129	53.251	712.97	3.565	11.380
1098.26	5.492	19.034	711.34	3.557	9.934
			710.41	3.553	9.249
			466.53	2.333	212.111

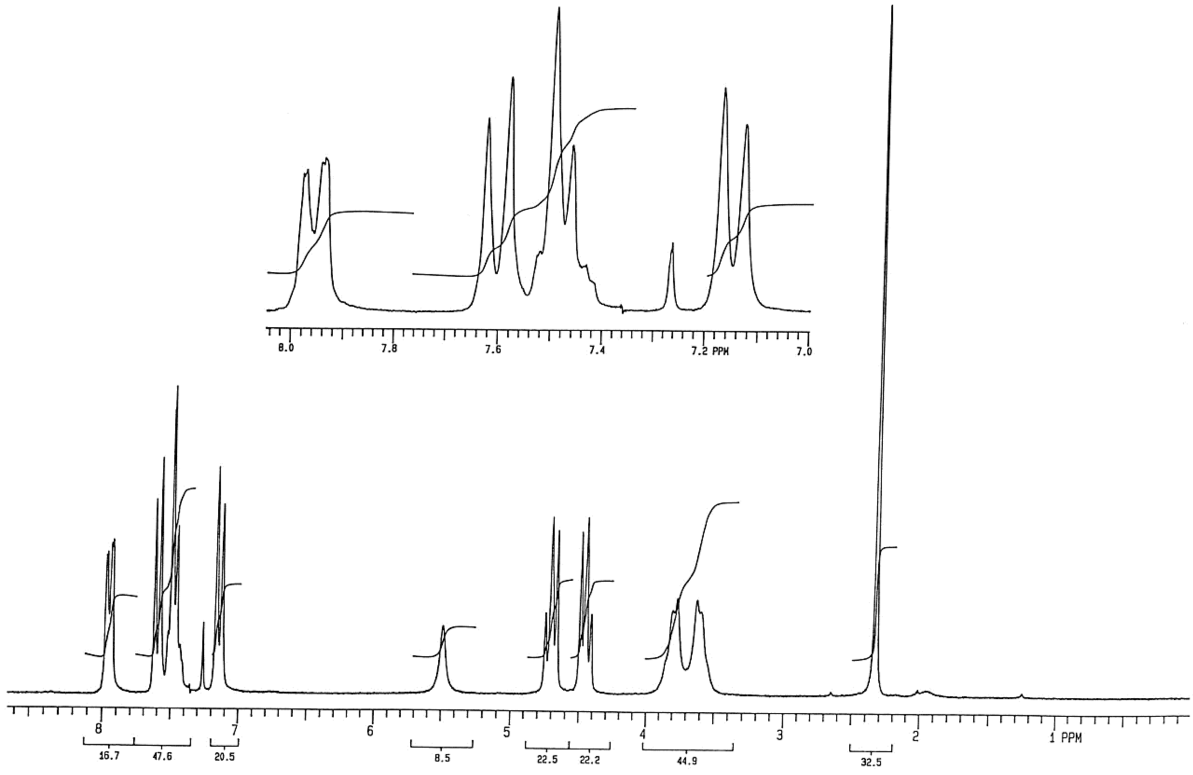

^{13}C-NMR ($CDCl_3$, 50 MHz) spectrum of *N*-(7-(4,5-dihydro-1*H*-imidazol-2-yl)-2-(*p*-tolyl)-6,7-dihydro-2*H*-imidazo[2,1-*c*][1,2,4]triazol-3(5*H*)-ylidene)benzenesulfonamide (**5d**)

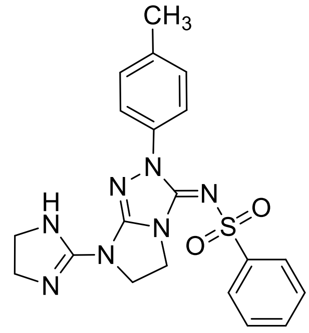

FREQ	PPM	INTENSITY
7790.9	154.921	20.448
7567.6	150.481	11.299
7252.5	144.215	8.796
7229.4	143.756	11.119
6899.9	137.203	24.574
6809.5	135.406	12.954
6649.2	132.219	60.377
6527.4	129.797	130.052
6493.8	129.128	101.538
6366.0	126.586	97.336
6139.1	122.076	110.743
3930.9	78.166	85.102
3899.0	77.531	86.971
3866.9	76.894	84.745
2669.8	53.088	10.867
2556.3	50.832	49.312
2338.8	46.507	8.841
2334.1	46.414	9.516
2303.5	45.805	50.216
1080.6	21.488	43.086

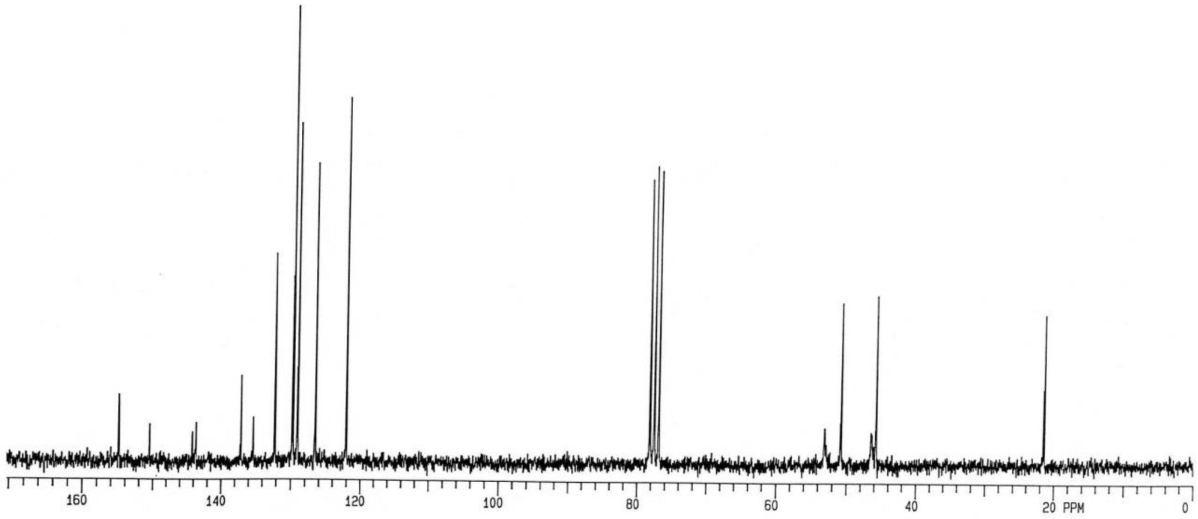

^{1}H-NMR ($CDCl_3$, 200 MHz) spectrum of *N*-(7-(4,5-dihydro-1*H*-imidazol-2-yl)-2-phenyl-6,7-dihydro-2*H*-imidazo[2,1-*c*][1,2,4]triazol-3(5*H*)-ylidene)-4-methylbenzenesulfonamide (**5e**)

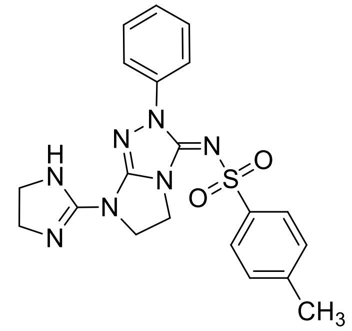

FREQ	PPM	INTENSITY	1435.34	7.178	6.015
1571.99	7.861	34.024	1098.76	5.495	6.307
1563.87	7.820	39.100	946.99	4.736	13.234
1556.03	7.781	30.497	939.56	4.698	27.593
1547.68	7.739	37.649	931.16	4.656	24.674
1477.57	7.389	15.376	895.48	4.478	24.703
1469.86	7.350	34.279	887.08	4.436	27.620
1461.97	7.311	25.117	879.71	4.399	12.822
1456.27	7.282	39.650	751.69	3.759	13.830
1448.57	7.244	42.529	727.23	3.637	13.702
1443.00	7.216	17.641	480.44	2.402	116.045

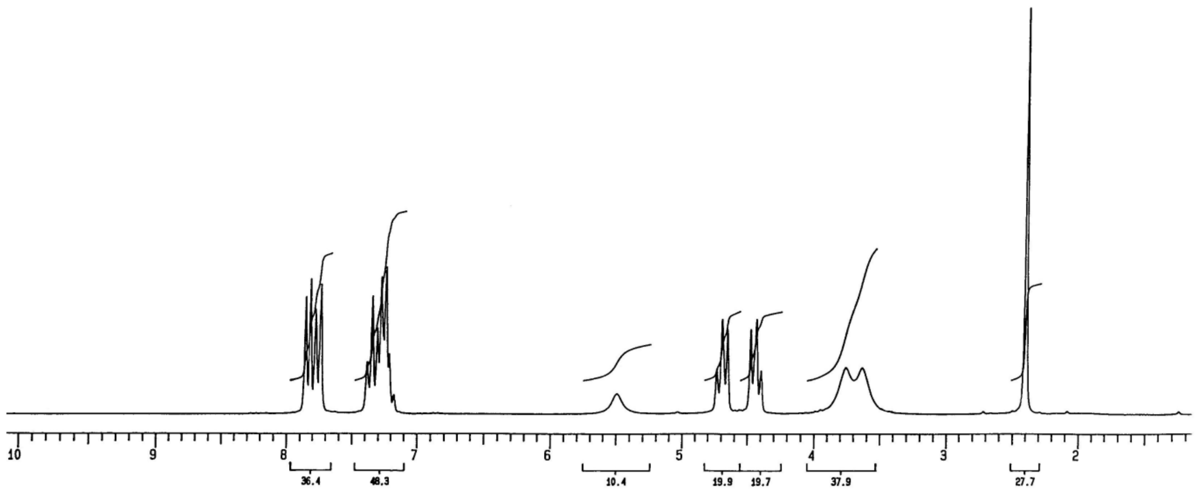

^{13}C-NMR ($CDCl_3$, 50 MHz) spectrum of *N*-(7-(4,5-dihydro-1*H*-imidazol-2-yl)-2-phenyl-6,7-dihydro-2*H*-imidazo[2,1-*c*][1,2,4]triazol-3(5*H*)-ylidene)-4-methylbenzenesulfonamide (**5e**)

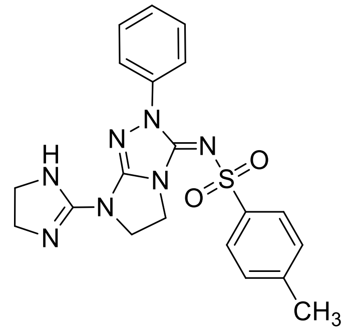

FREQ	PPM	INTENSIT'
7790.1	154.905	21.417
7569.8	150.524	14.129
7256.4	144.292	8.866
7183.6	142.845	20.560
7085.5	140.895	13.908
6937.8	137.958	13.523
6525.3	129.755	103.884
6498.3	129.219	117.942
6387.6	127.017	56.356
6367.2	126.611	117.976
6129.3	121.881	130.052
3931.6	78.180	55.269
3899.6	77.543	55.958
3867.6	76.906	56.492
2555.9	50.823	53.245
2305.7	45.850	52.958
1104.7	21.967	36.959

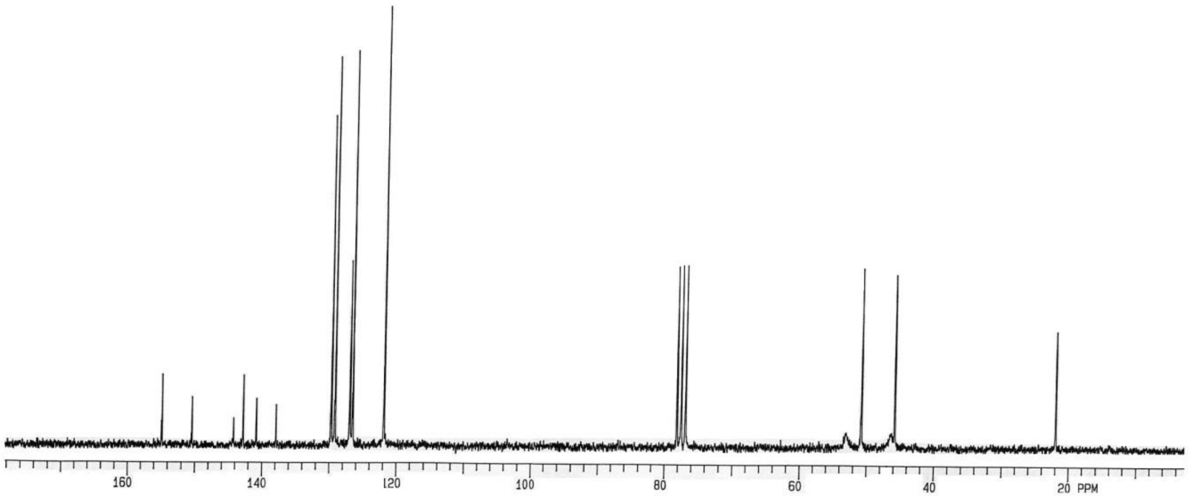

^{1}H-NMR ($CDCl_3$, 200 MHz) spectrum of *N*-(7-(4,5-dihydro-1*H*-imidazol-2-yl)-2-(*p*-tolyl)-6,7-dihydro-2*H*-imidazo[2,1-*c*][1,2,4]triazol-3(5*H*)-ylidene)-4-methylbenzenesulfonamide (**5f**)

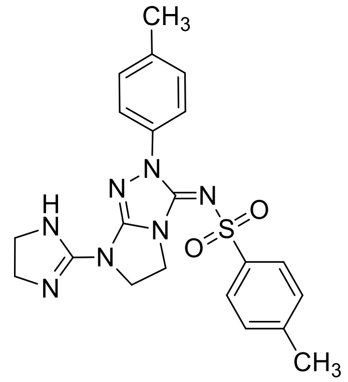

FREQ	PPM	INTENSITY	880.93	4.405	20.143
1571.31	7.858	48.597	760.58	3.803	24.923
1563.19	7.817	56.598	758.49	3.793	23.885
1526.87	7.635	48.467	754.31	3.772	27.119
1518.47	7.593	58.030	745.63	3.729	9.414
1456.01	7.281	56.770	744.23	3.722	8.732
1453.33	7.268	37.703	737.97	3.690	8.372
1447.94	7.241	49.403	726.14	3.631	27.076
			723.17	3.616	24.133
1433.79	7.170	56.674	719.75	3.599	23.880
1425.43	7.128	47.471	711.34	3.557	10.672
1098.96	5.495	19.009	710.36	3.552	9.835
947.52	4.738	21.388	709.20	3.546	9.020
940.27	4.702	45.608	708.09	3.541	8.004
931.69	4.659	39.679	706.93	3.535	6.868
896.71	4.484	40.517	480.97	2.405	182.186
888.18	4.441	44.063	466.17	2.331	179.284

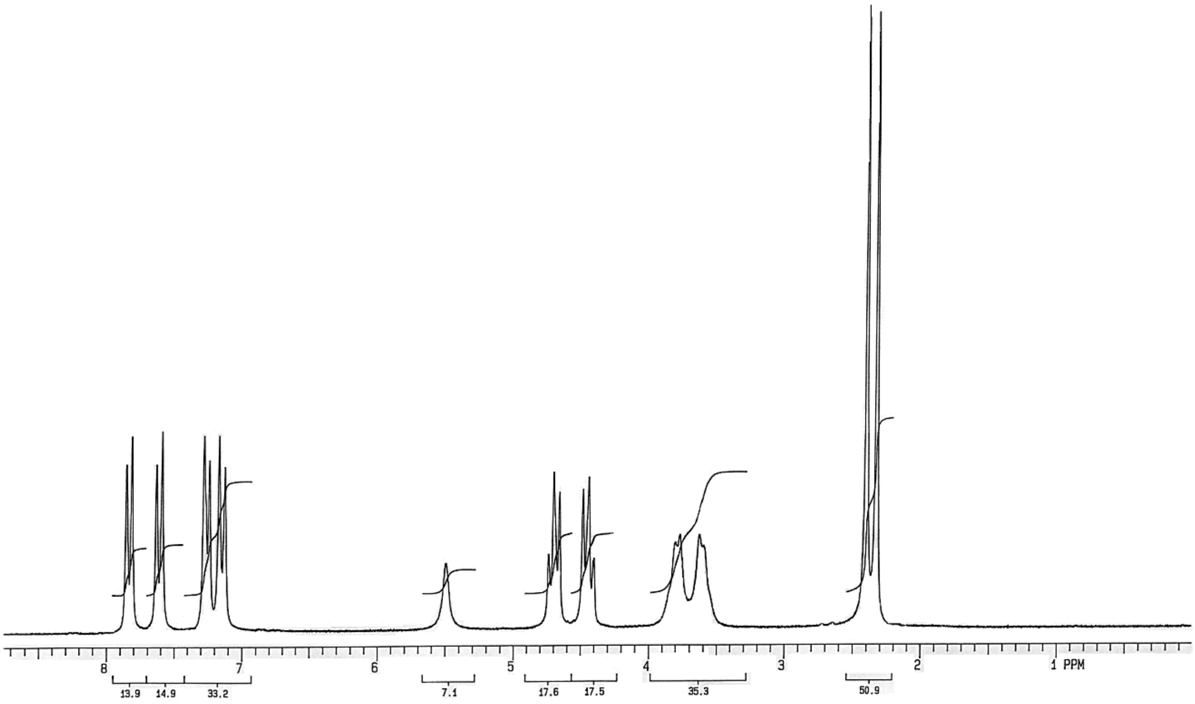

^{13}C-NMR ($CDCl_3$, 50 MHz) spectrum of *N*-(7-(4,5-dihydro-1*H*-imidazol-2-yl)-2-(*p*-tolyl)-6,7-dihydro-2*H*-imidazo[2,1-*c*][1,2,4]triazol-3(5*H*)-ylidene)-4-methylbenzenesulfonamide (**5f**)

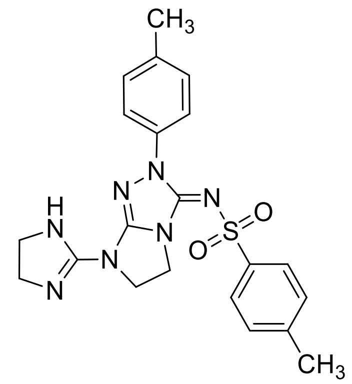

FREQ	PPM	INTENSITY
7792.1	154.946	21.280
7565.9	150.447	12.098
7251.5	144.195	9.483
7179.0	142.754	20.037
7089.5	140.974	11.584
6893.5	137.076	21.592
6812.9	135.475	13.287
6526.3	129.775	129.912
6523.8	129.724	130.052
6367.1	126.609	102.678
6135.7	122.009	114.628
3930.8	78.164	83.222
3898.9	77.529	86.629
3866.9	76.894	85.168
2555.6	50.817	51.797
2333.2	46.395	6.842
2304.3	45.821	51.142
1104.5	21.963	40.013
1080.3	21.481	39.009

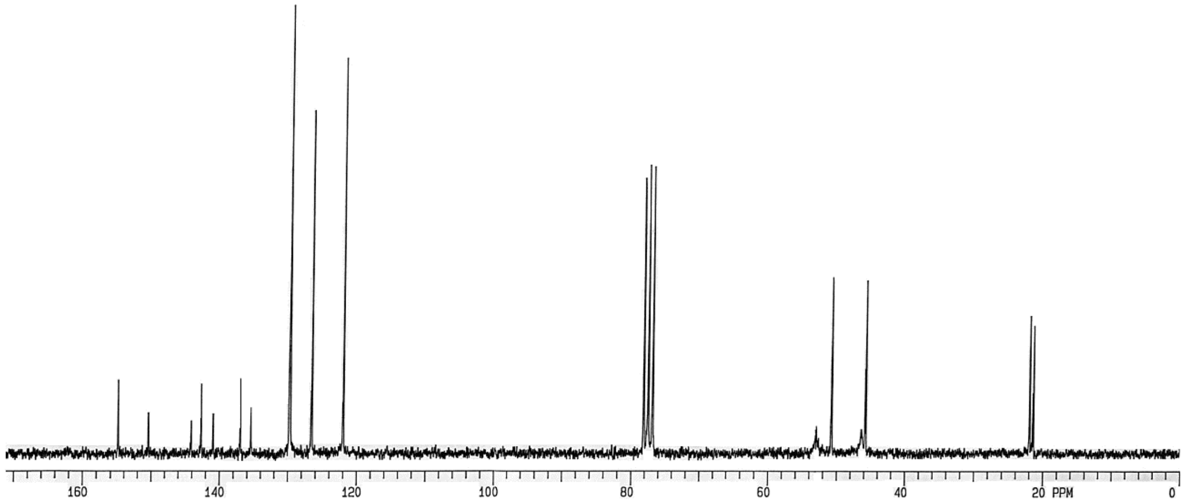

^{1}H-NMR ($CDCl_3$, 200 MHz) spectrum of *N*-(7-(4,5-dihydro-1*H*-imidazol-2-yl)-2-phenyl-6,7-dihydro-2*H*-imidazo[2,1-*c*][1,2,4]triazol-3(5*H*)-ylidene)-4-methoxybenzenesulfonamide(**5g**)

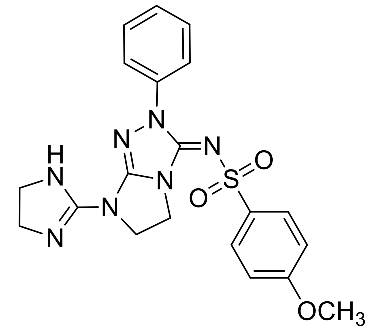

FREQUENCY	PPM	HEIGHT
1582.434	7.914	34.4
1574.230	7.873	38.1
1555.516	7.779	32.9
1547.825	7.741	39.6
1478.352	7.393	18.4
1471.174	7.357	38.4
1463.740	7.320	26.5
1451.947	7.261	21.8
1444.513	7.224	17.2
1437.335	7.188	7.0
1393.241	6.967	42.2
1385.038	6.926	39.7
950.511	4.753	18.1
943.076	4.716	38.6
935.129	4.676	32.7
899.752	4.500	32.4
891.805	4.460	39.2
884.627	4.424	19.0
770.804	3.855	137.4
742.604	3.714	39.5
250.909	1.255	5.0

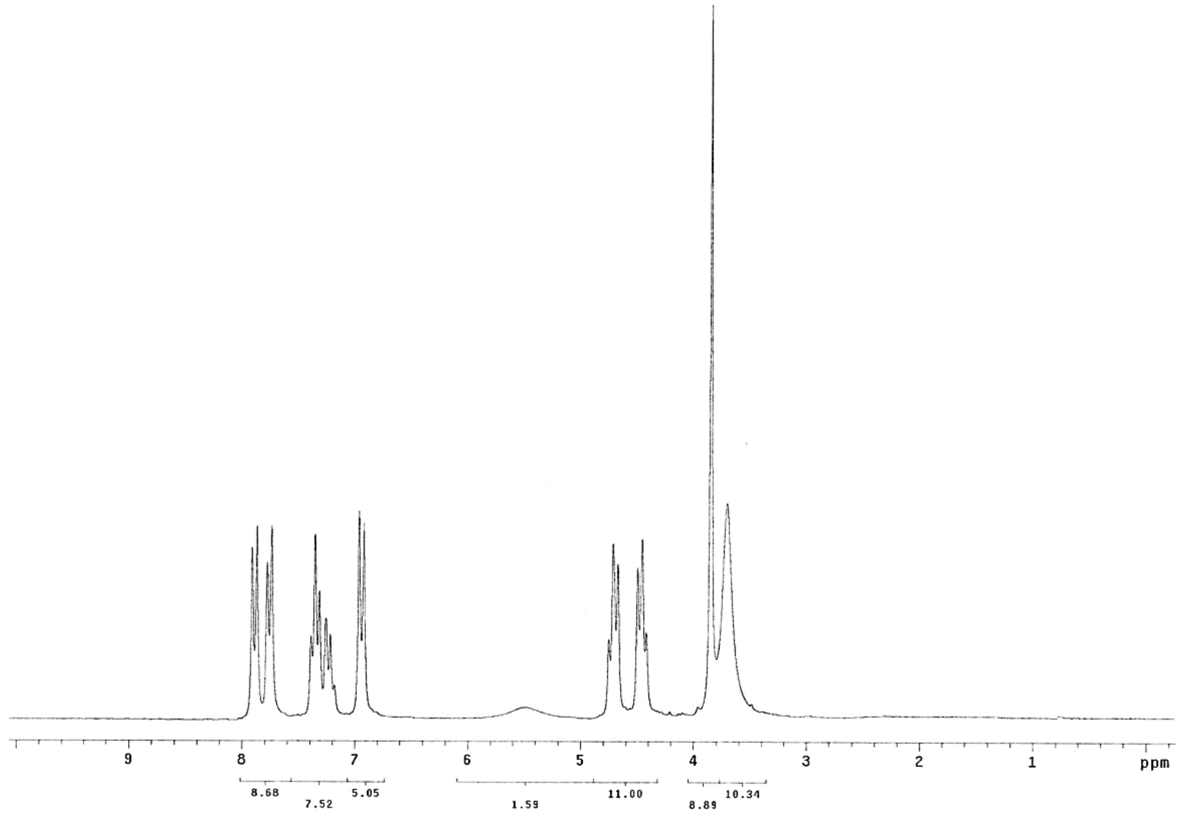

^{13}C-NMR ($CDCl_3$, 50 MHz) spectrum of *N*-(7-(4,5-dihydro-1*H*-imidazol-2-yl)-2-phenyl-6,7-dihydro-2*H*-imidazo[2,1-*c*][1,2,4]triazol-3(5*H*)-ylidene)-4-methoxybenzenesulfonamide (**5g**)

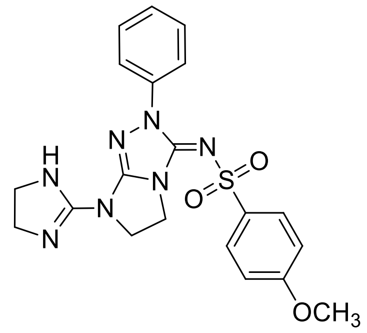

FREQUENCY	PPM	HEIGHT
8152.935	162.147	13.5
7762.124	154.375	16.2
7539.736	149.952	9.3
7225.521	143.702	7.7
6909.564	137.419	11.5
6796.412	135.168	11.9
6471.316	128.703	84.4
6441.723	128.114	81.8
6359.905	126.487	41.3
6100.524	121.328	83.9
5719.723	113.755	79.5
3904.062	77.645	70.6
3871.857	77.004	71.7
3840.088	76.372	72.4
2790.817	55.504	36.5
2530.131	50.320	39.7
2279.455	45.334	38.8

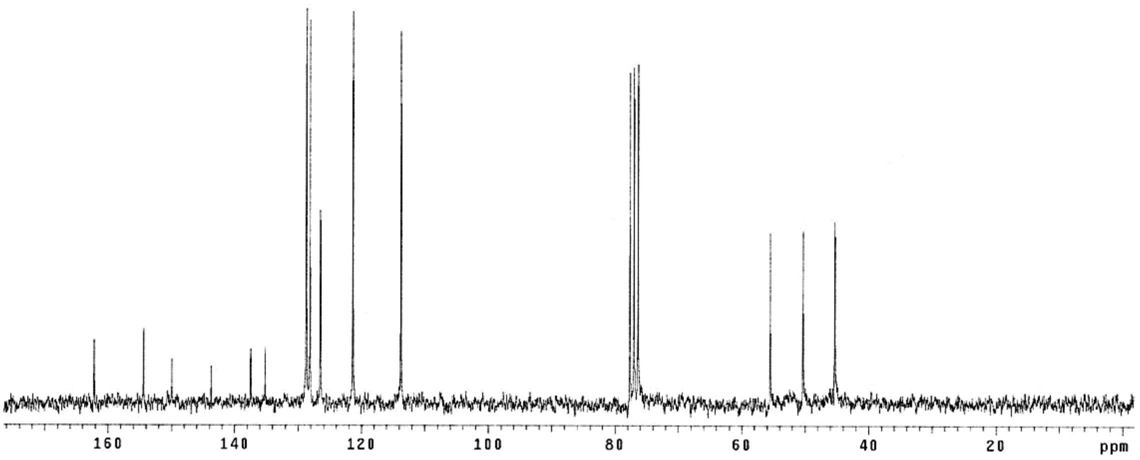

^{1}H-NMR (DMSO-d_6, 200 MHz) spectrum of *N*-(7-(4,5-dihydro-1*H*-imidazol-2-yl)-2-phenyl-6,7-dihydro-2*H*-imidazo[2,1-*c*][1,2,4]triazol-3(5*H*)-ylidene)-4-nitrobenzenesulfonamide (**5h**)

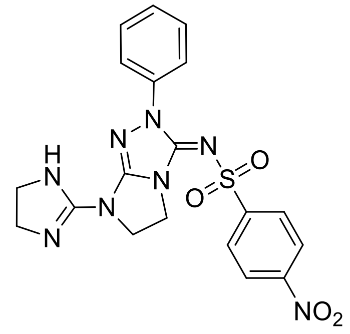

1677.179	8.387	13.7
1668.461	8.344	18.0
1624.459	8.124	18.4
1616.157	8.082	13.9
1558.871	7.796	14.7
1550.984	7.756	17.1
1491.623	7.459	15.5
1483.735	7.420	10.7
1469.621	7.349	7.6
1462.564	7.314	7.3
872.271	4.362	17.6
866.875	4.335	17.6
702.905	3.515	42.6
668.035	3.341	180.5
499.914	2.500	120.2
396.965	1.985	4.3
245.863	1.230	9.6
165.331	0.827	5.0

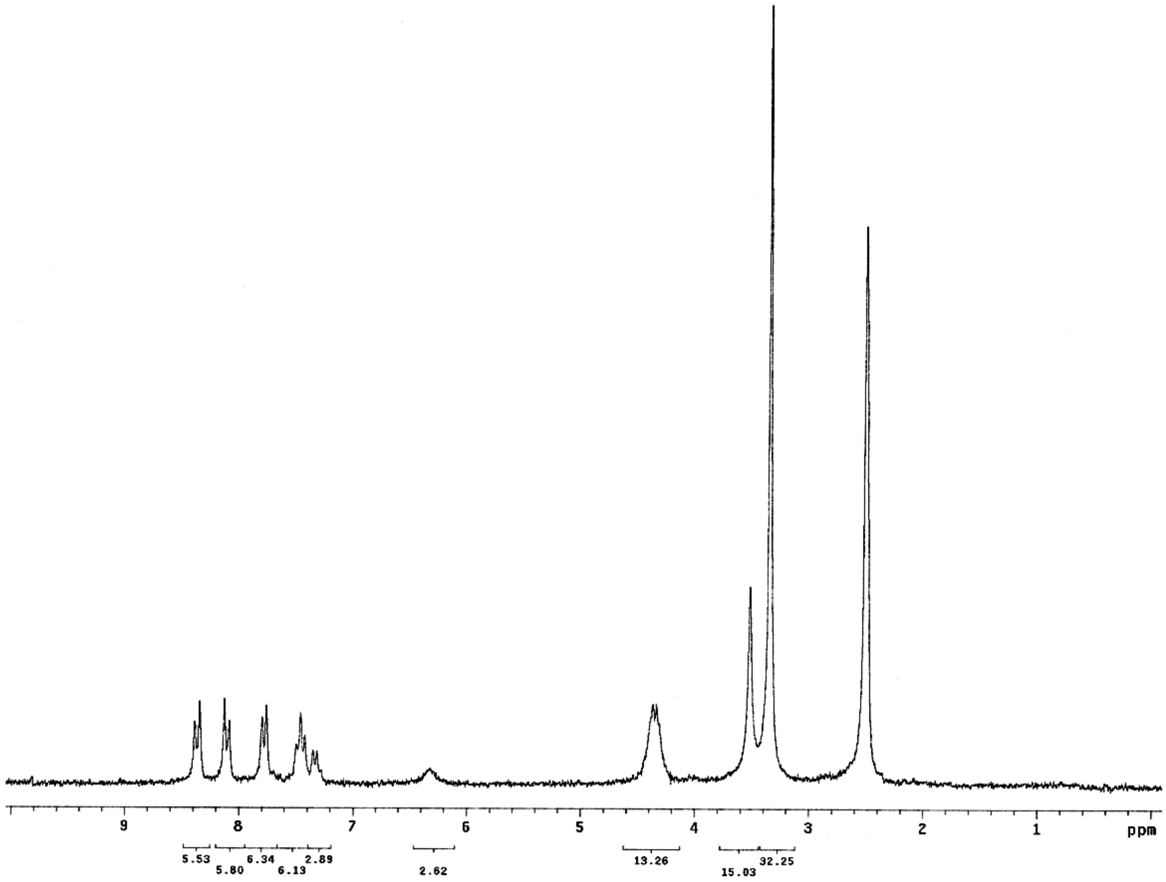

^{1}H-NMR ($CDCl_3$, 200 MHz) spectrum of *N*-(7-(4,5-dihydro-1*H*-imidazol-2-yl)-2-phenyl-6,7-dihydro-2*H*-imidazo[2,1-*c*][1,2,4]triazol-3(5*H*)-ylidene)naphthalene-1-sulfonamide(**5i**)

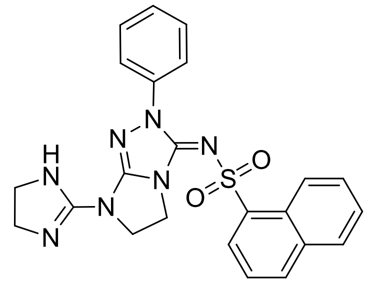

INDEX	FREQUENCY	PPM	HEIGHT	INDEX	FREQUENCY	PPM	HEIGHT	INDEX	FREQUENCY	PPM	HEIGHT	INDEX
1	1771.720	8.860	12.3	11	1580.352	7.903	15.0	21	1510.613	7.554	22.4	31
2	1769.229	8.848	11.6	12	1578.276	7.893	16.6	22	1508.537	7.544	22.2	32
3	1762.172	8.812	14.3	13	1540.086	7.702	25.3	23	1500.650	7.505	24.6	33
4	1660.469	8.304	19.1	14	1538.425	7.693	27.5	24	1492.763	7.465	14.0	34
5	1654.242	8.273	19.7	15	1532.199	7.662	34.7	25	1453.742	7.270	14.0	35
6	1652.997	8.266	20.3	16	1530.538	7.654	33.3	26	1449.591	7.249	7.7	36
7	1607.334	8.038	18.5	17	1522.651	7.615	20.3	27	1446.270	7.233	10.7	37
8	1599.447	7.999	21.8	18	1520.160	7.602	26.8	28	1440.873	7.206	33.2	38
9	1587.409	7.938	13.8	19	1516.424	7.583	33.5	29	1432.986	7.166	56.4	39
10	1583.258	7.918	10.4	20	1512.688	7.565	28.8	30	1427.590	7.139	12.7	40

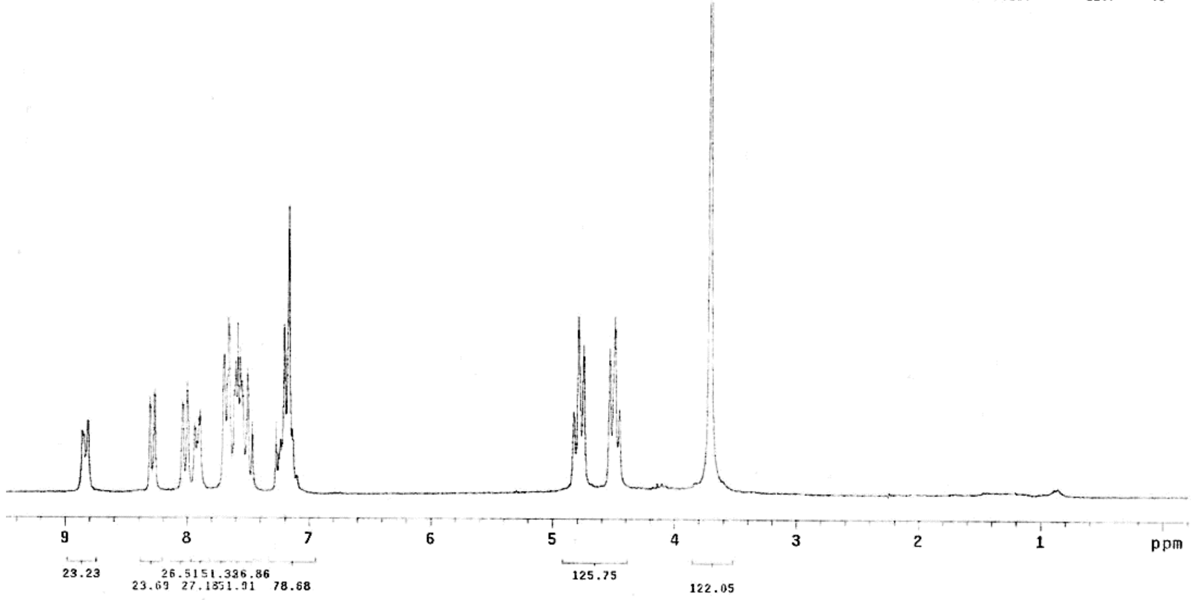

^{13}C-NMR (CDCl$_3$, 50 MHz) spectrum of *N*-(7-(4,5-dihydro-1*H*-imidazol-2-yl)-2-phenyl-6,7-dihydro-2*H*-imidazo[2,1-*c*][1,2,4]triazol-3(5*H*)-ylidene)naphthalene-1-sulfonamide (**5i**)

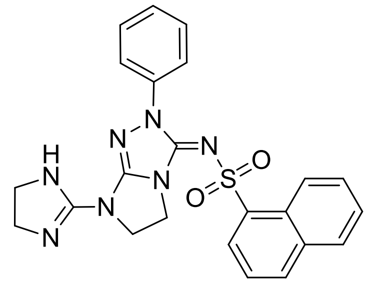

INDEX	FREQUENCY	PPM	HEIGHT	INDEX	FREQUENCY	PPM	HEIGHT	INDEX	FREQUENCY	PPM	HEIGHT
1	19434.547	154.639	0.2	11	16048.142	127.693	0.4	21	5754.999	45.792	0.4
2	18888.296	150.292	0.1	12	15951.538	126.925	0.4				
3	18082.093	143.877	0.1	13	15941.878	126.848	0.5				
4	17413.770	138.559	0.2	14	15864.156	126.229	0.4				
5	17273.255	137.441	0.1	15	15622.207	124.304	0.4				
6	16900.891	134.479	0.2	16	15299.023	121.733	0.9				
7	16793.309	133.623	0.4	17	9746.056	77.548	0.8				
8	16198.756	128.892	0.9	18	9714.001	77.293	0.8				
9	16186.022	128.790	0.4	19	9681.946	77.038	0.8				
10	16172.410	128.682	0.1	20	6362.725	50.628	0.4				

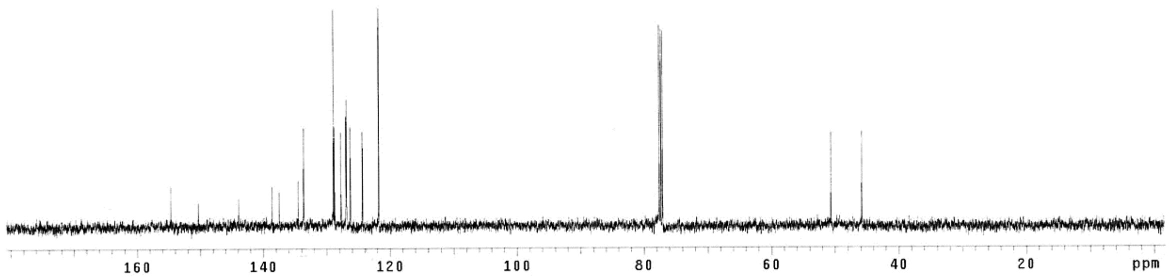

^{1}H-NMR ($CDCl_3$, 200 MHz) spectrum of *N*-(7-(4,5-dihydro-1*H*-imidazol-2-yl)-2-phenyl-6,7-dihydro-2*H*-imidazo[2,1-*c*][1,2,4]triazol-3(5*H*)-ylidene)naphthalene-2-sulfonamide (**5j**)

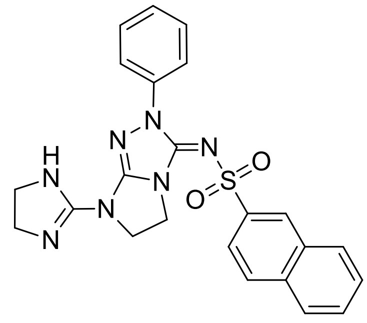

INDEX	FREQUENCY	PPM	HEIGHT	INDEX	FREQUENCY	PPM	HEIGHT
1	1702.811	8.516	17.1	11	1103.800	5.520	4.8
2	1591.975	7.961	49.3	12	962.661	4.814	12.6
3	1558.766	7.795	20.9	13	955.604	4.779	24.1
4	1551.294	7.758	23.7	14	947.717	4.739	19.6
5	1523.066	7.617	19.5	15	906.621	4.534	19.4
6	1518.915	7.596	20.0	16	898.734	4.494	23.8
7	1470.346	7.353	23.2	17	891.677	4.459	13.4
8	1462.874	7.316	17.9	18	754.689	3.774	16.9
9	1452.912	7.266	15.6	19	733.103	3.666	17.2
10	1445.440	7.228	11.6	20			

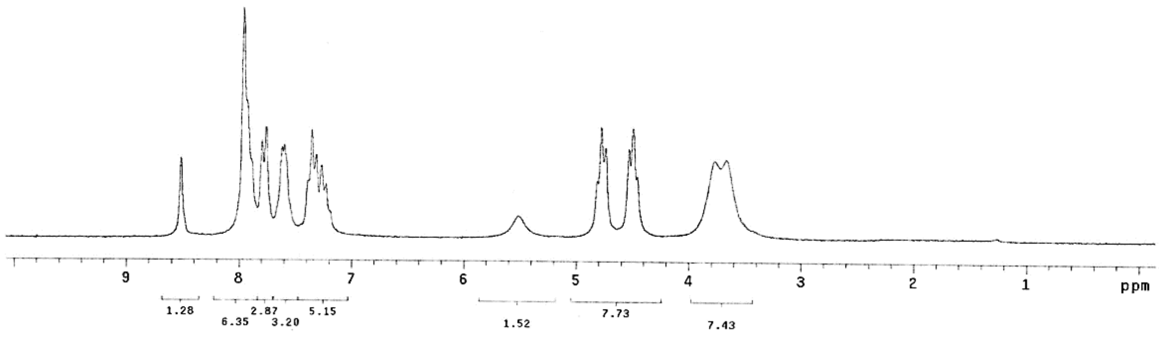

^{13}C-NMR ($CDCl_3$, 50 MHz) spectrum of *N*-(7-(4,5-dihydro-1*H*-imidazol-2-yl)-2-phenyl-6,7-dihydro-2*H*-imidazo[2,1-*c*][1,2,4]triazol-3(5*H*)-ylidene)naphthalene-2-sulfonamide (**5j**)

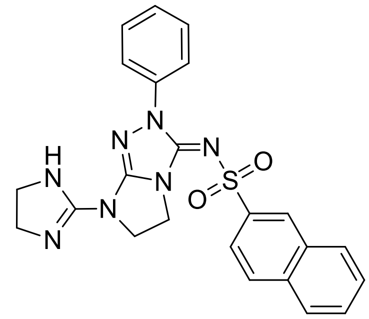

INDEX	FREQUENCY	PPM	HEIGHT	INDEX	REQUENCY	PPM	HEIGHT	INDEX	FREQUENCY	PPM	HEIGHT
1	7759.302	154.318	11.7	11	6446.299	128.205	32.0	21	2531.225	50.341	27.9
2	7542.571	150.008	7.4	12	6423.668	127.755	32.7	22	2306.661	45.875	23.0
3	7227.050	143.733	4.4	13	6391.463	127.115	32.7	23	2278.373	45.313	26.3
4	7043.395	140.080	7.3	14	6363.610	126.561	58.6				
5	6905.871	137.345	7.5	15	6151.232	122.337	31.9				
6	6759.644	134.437	8.8	16	6102.489	121.367	61.5				
7	6639.093	132.039	9.0	17	3905.592	77.675	27.6				
8	6495.041	129.175	34.5	18	3873.822	77.043	28.0				
9	6482.420	128.924	37.5	19	3841.617	76.403	27.8				
10	6471.540	128.707	65.8	20	2641.767	52.540	22.2				

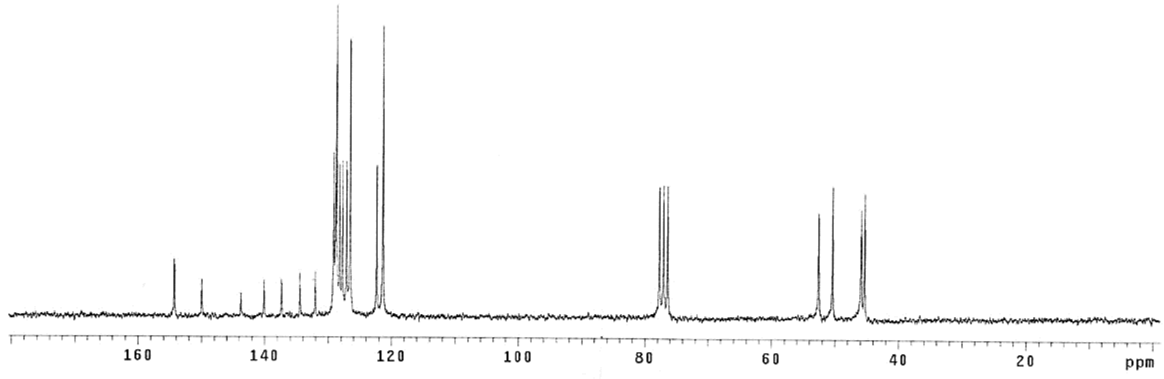

^{1}H-NMR ($CDCl_3$, 500 MHz) spectrum of *N*-(7-(4,5-dihydro-1*H*-imidazol-2-yl)-2-phenyl-6,7-dihydro-2*H*-imidazo[2,1-*c*][1,2,4]triazol-3(5*H*)-ylidene)-[1,1'-biphenyl]-4-sulfonamide (**5k**)

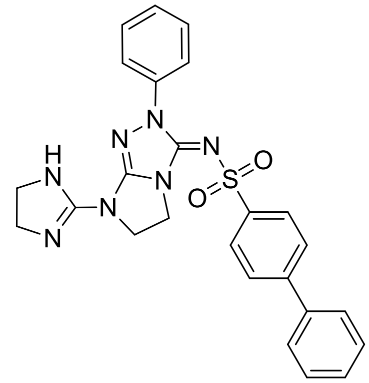

FREQUENCY	PPM	HEIGHT	FREQUENCY	PPM	HEIGHT
4014.597	8.032	17.7	3691.336	7.385	10.2
4005.808	8.015	17.6	3684.011	7.371	15.9
3890.078	7.783	12.9	3675.710	7.354	9.1
3889.102	7.781	13.5	3631.762	7.266	3.8
3881.289	7.766	16.0	3630.785	7.264	5.7
3846.130	7.695	20.6	3627.855	7.258	4.7
3837.829	7.679	17.0	3626.878	7.257	5.4
3805.112	7.613	14.1	3619.554	7.242	7.9
3797.787	7.598	17.6	3611.741	7.226	3.1
3740.655	7.484	7.5	2758.173	5.518	0.8
3739.678	7.482	7.2	2378.755	4.759	8.9
3732.842	7.469	15.6	2370.942	4.744	14.9
3725.029	7.453	9.9	2362.153	4.726	11.9
3706.473	7.416	4.7	2250.818	4.503	11.7
3705.497	7.414	5.6	2242.517	4.487	15.2
3698.660	7.400	6.9	2234.704	4.471	9.0
			1895.816	3.793	2.5
			1817.198	3.636	2.5

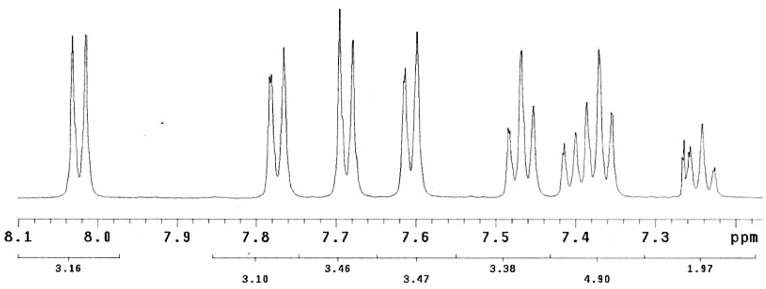

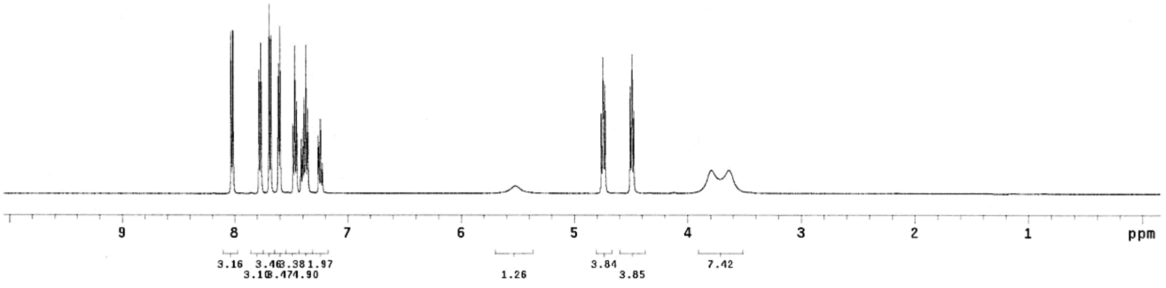

^{13}C-NMR ($CDCl_3$, 125 MHz) spectrum of *N*-(7-(4,5-dihydro-1*H*-imidazol-2-yl)-2-phenyl-6,7-dihydro-2*H*-imidazo[2,1-*c*][1,2,4]triazol-3(5*H*)-ylidene)-[1,1'-biphenyl]-4-sulfonamide (**5k**)

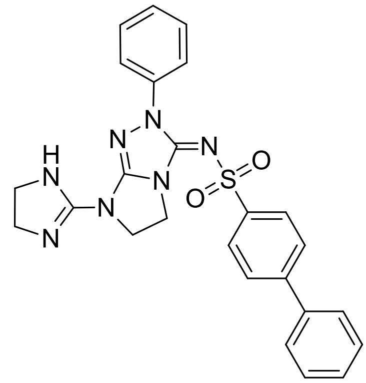

19438.938	154.673	0.2
18891.809	150.320	0.1
18219.095	144.967	0.2
18105.805	144.066	0.1
17853.317	142.057	0.2
17583.265	139.908	0.2
17295.649	137.620	0.2
16238.715	129.210	0.9
16218.516	129.049	1.0
16144.307	128.458	0.4
16038.482	127.616	1.0
16030.139	127.550	1.0
15956.807	126.967	0.4
15947.147	126.890	1.0
15305.609	121.785	1.0
9745.178	77.541	1.3
9713.123	77.286	1.3
9681.068	77.031	1.4
6361.847	50.621	0.4
5732.604	45.614	0.4

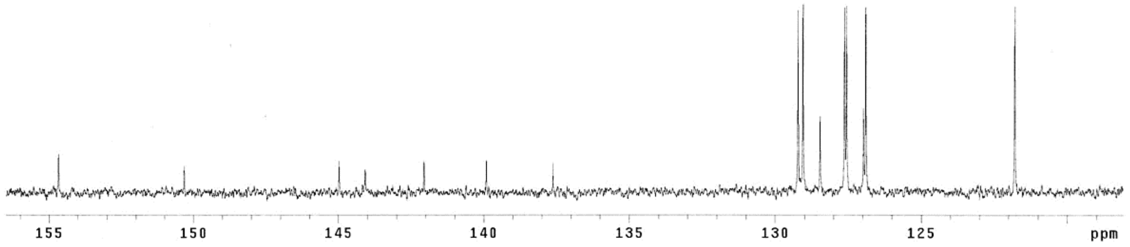

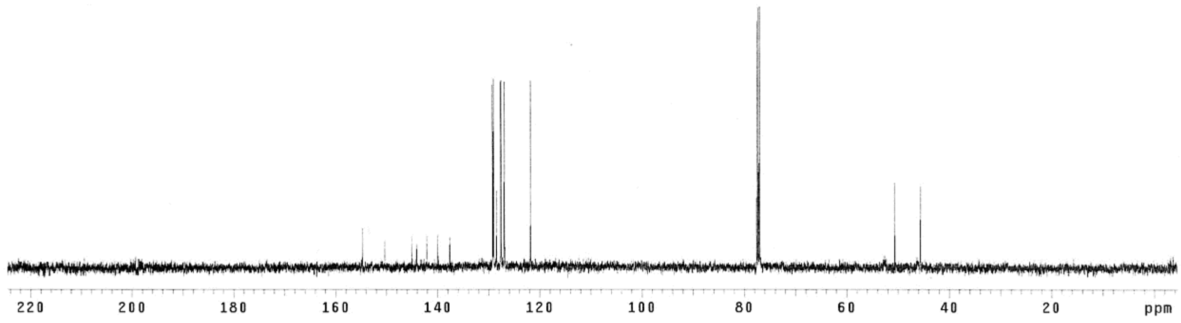

^{1}H-NMR ($CDCl_3$, 300 MHz) spectrum of *N*-(7-(4,5-dihydro-1*H*-imidazol-2-yl)-2-(*p*-tolyl)-6,7-dihydro-2*H*-imidazo[2,1-*c*][1,2,4]triazol-3(5*H*)-ylidene)-[1,1'-biphenyl]-4-sulfonamide (**5l**)

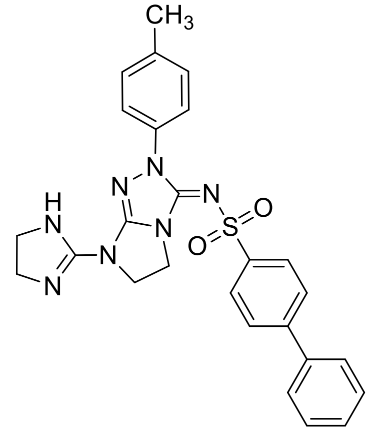

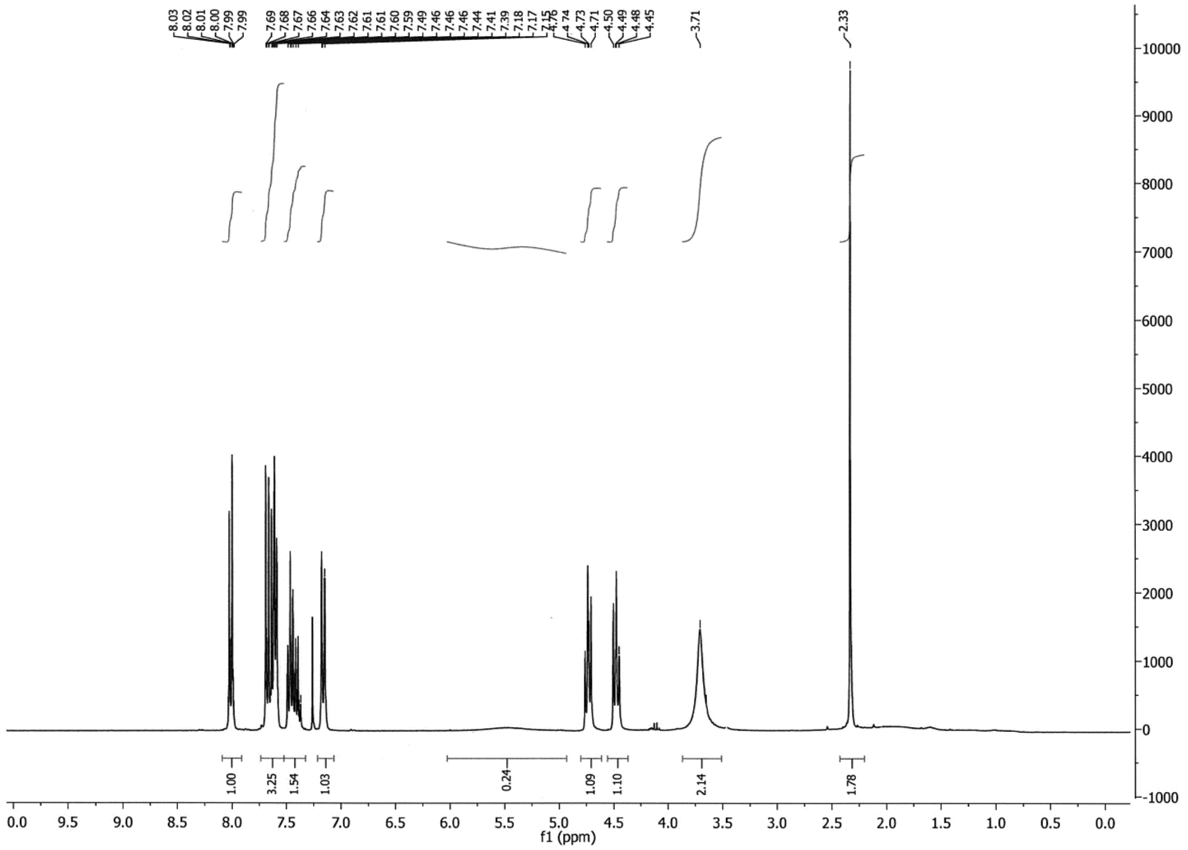

^{13}C-NMR ($CDCl_3$, 75 MHz) spectrum of *N*-(7-(4,5-dihydro-1*H*-imidazol-2-yl)-2-(*p*-tolyl)-6,7-dihydro-2*H*-imidazo[2,1-*c*][1,2,4]triazol-3(5*H*)-ylidene)-[1,1'-biphenyl]-4-sulfonamide (**5l**)

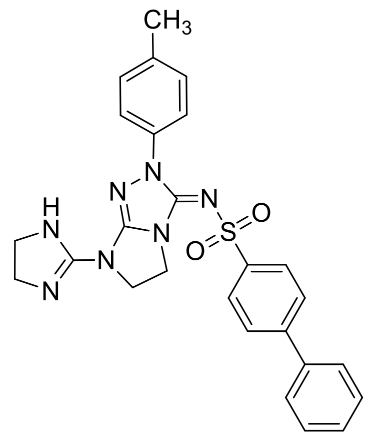

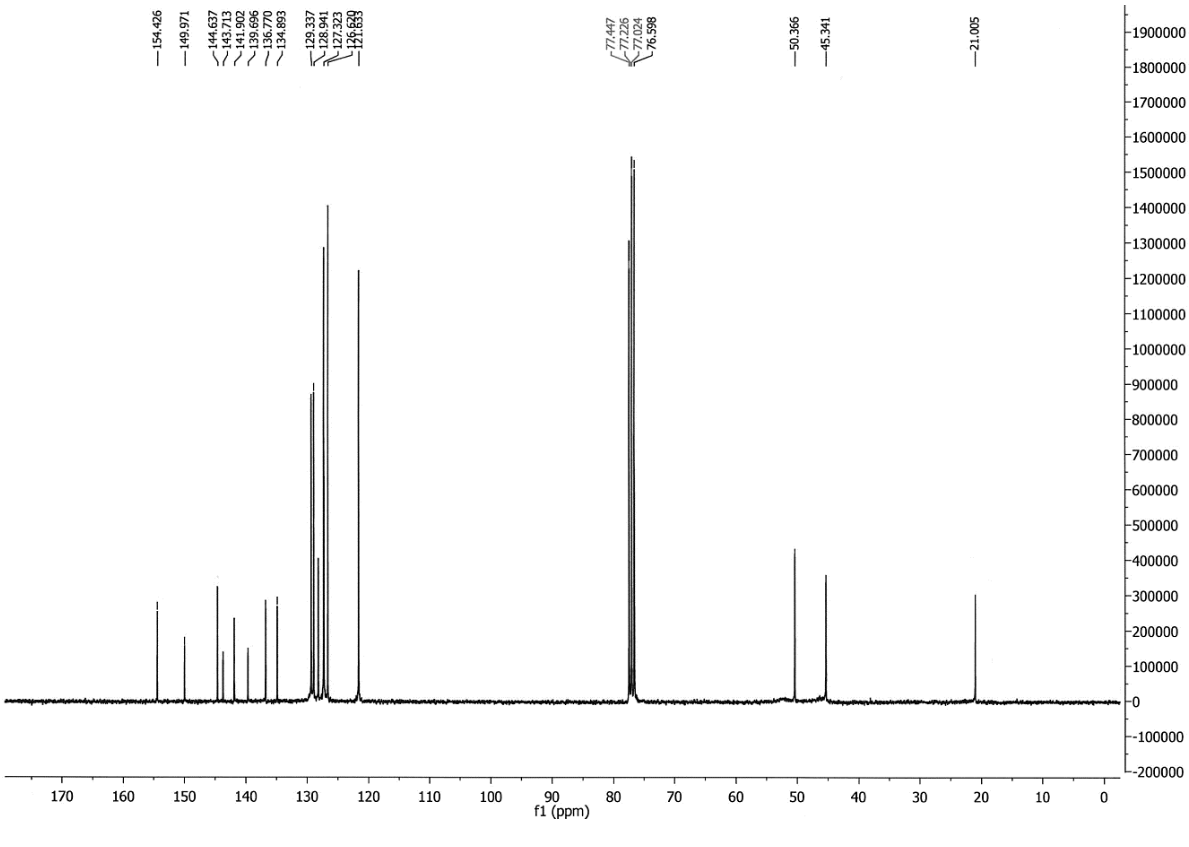

^{13}C-NMR ($CDCl_3$, 75 MHz) spectrum of *N*-(7-(4,5-dihydro-1*H*-imidazol-2-yl)-2-(*p*-tolyl)-6,7-dihydro-2*H*-imidazo[2,1-*c*][1,2,4]triazol-3(5*H*)-ylidene)-[1,1'-biphenyl]-4-sulfonamide (**5l**) - (*aromatic region*)

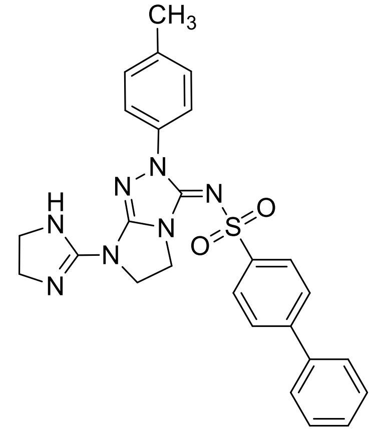

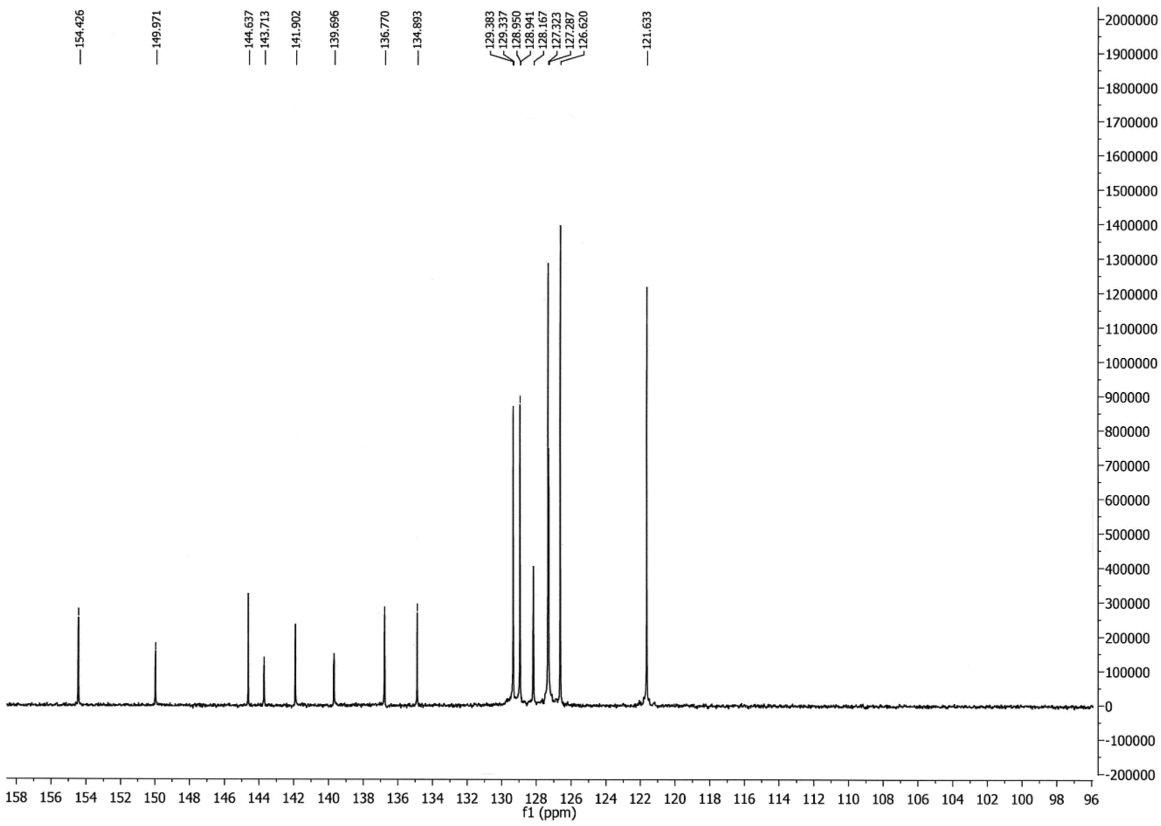

^{1}H-NMR (DMSO-d_6, 500 MHz) spectrum of *N*-(7-(4,5-dihydro-1*H*-imidazol-2-yl)-2-phenyl-6,7-dihydro-2*H*-imidazo[2,1-*c*][1,2,4]triazol-3(5*H*)-ylidene)-4-phenoxybenzenesulfonamide (**5m**)

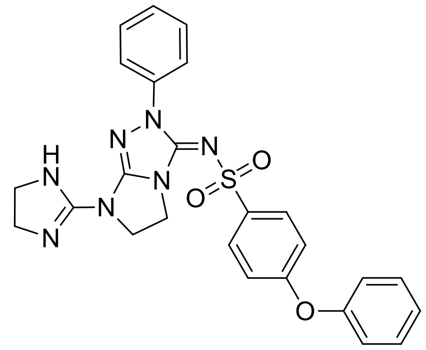

INDEX	FREQUENCY	PPM	HEIGHT	INDEX	FREQUENCY	PPM	HEIGHT	INDEX	FREQUENCY	PPM	HEIGHT
1	3924.269	7.851	11.0	17	3537.039	7.077	11.2	33	1575.005	3.151	1.4
2	3915.480	7.834	11.4	18	3528.249	7.059	10.5	34	1243.442	2.488	14.3
3	3904.249	7.811	9.0	19	3055.564	6.113	6.3				
4	3896.436	7.796	9.5	20	2208.344	4.418	4.1				
5	3728.945	7.461	5.1	21	2201.020	4.404	8.5				
6	3721.620	7.446	13.5	22	2198.578	4.399	5.9				
7	3713.807	7.430	14.3	23	2192.718	4.387	7.5				
8	3706.483	7.416	6.3	24	2152.677	4.307	7.3				
9	3644.955	7.293	3.2	25	2144.375	4.290	8.2				
10	3637.631	7.278	5.3	26	2137.051	4.276	3.8				
11	3630.306	7.263	2.3	27	2051.596	4.105	0.6				
12	3620.051	7.243	2.9	28	2046.225	4.094	0.7				
13	3612.727	7.228	4.8	29	1772.282	3.546	1.9				
14	3604.914	7.213	2.1	30	1726.870	3.455	1.9				
15	3560.478	7.124	8.7	31	1663.389	3.328	16.7				
16	3552.665	7.108	7.9	32	1580.376	3.162	1.5				

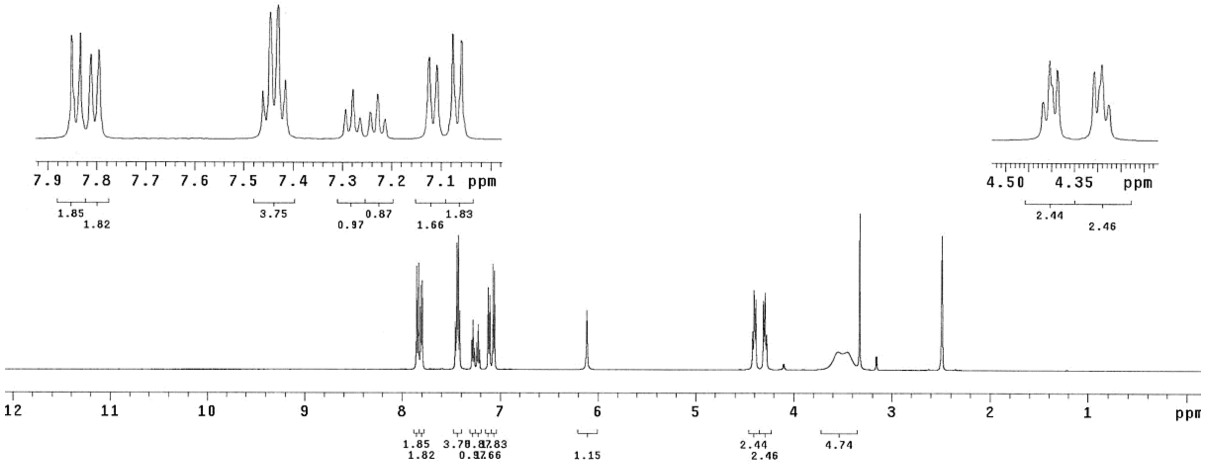

^{13}C-NMR (DMSO-d_6+TFA, 125 MHz) spectrum of *N*-(7-(4,5-dihydro-1*H*-imidazol-2-yl)-2-phenyl-6,7-dihydro-2*H*-imidazo[2,1-*c*][1,2,4]triazol-3(5*H*)-ylidene)-4-phenoxybenzenesulfonamide (**5m**)

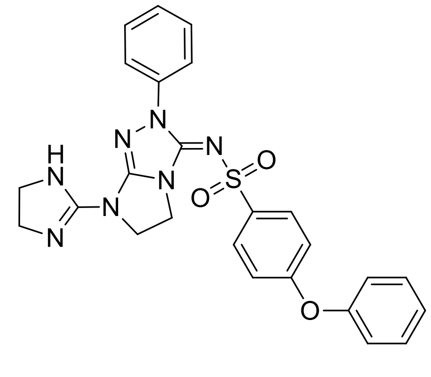

INDEX	FREQUENCY	PPM	HEIGHT	INDEX	FREQUENCY	PPM	HEIGHT	INDEX	FREQUENCY	PPM	HEIGHT
1	20195.118	160.690	0.3	17	15375.462	122.340	1.1	33	5036.652	40.076	0.5
2	20054.164	159.568	0.2	18	15161.177	120.635	1.2	34	5025.235	39.985	3.6
3	20015.961	159.264	0.7	19	14979.386	119.189	0.2	35	5004.158	39.817	3.1
4	19977.759	158.960	0.8	20	14852.484	118.179	1.0	36	4983.080	39.650	1.5
5	19939.117	158.653	0.3	21	14691.331	116.897	0.5	37	4962.442	39.485	0.5
6	19564.558	155.672	0.2	22	14402.837	114.601	0.5				
7	19340.612	153.890	0.3	23	14114.343	112.306	0.2				
8	18797.874	149.572	0.2	24	6612.173	52.612	0.3				
9	18123.404	144.205	0.2	25	5843.294	46.494	0.3				
10	17387.458	138.349	0.2	26	5567.095	44.297	0.4				
11	17308.418	137.721	0.3	27	5099.444	40.576	0.1				
12	16461.817	130.984	1.2	28	5088.466	40.488	0.5				
13	16292.321	129.636	1.0	29	5078.806	40.411	0.3				
14	16182.105	128.759	1.0	30	5067.389	40.320	1.5				
15	16056.081	127.756	0.3	31	5057.290	40.240	0.5				
16	15764.073	125.432	0.6	32	5046.312	40.153	3.1				

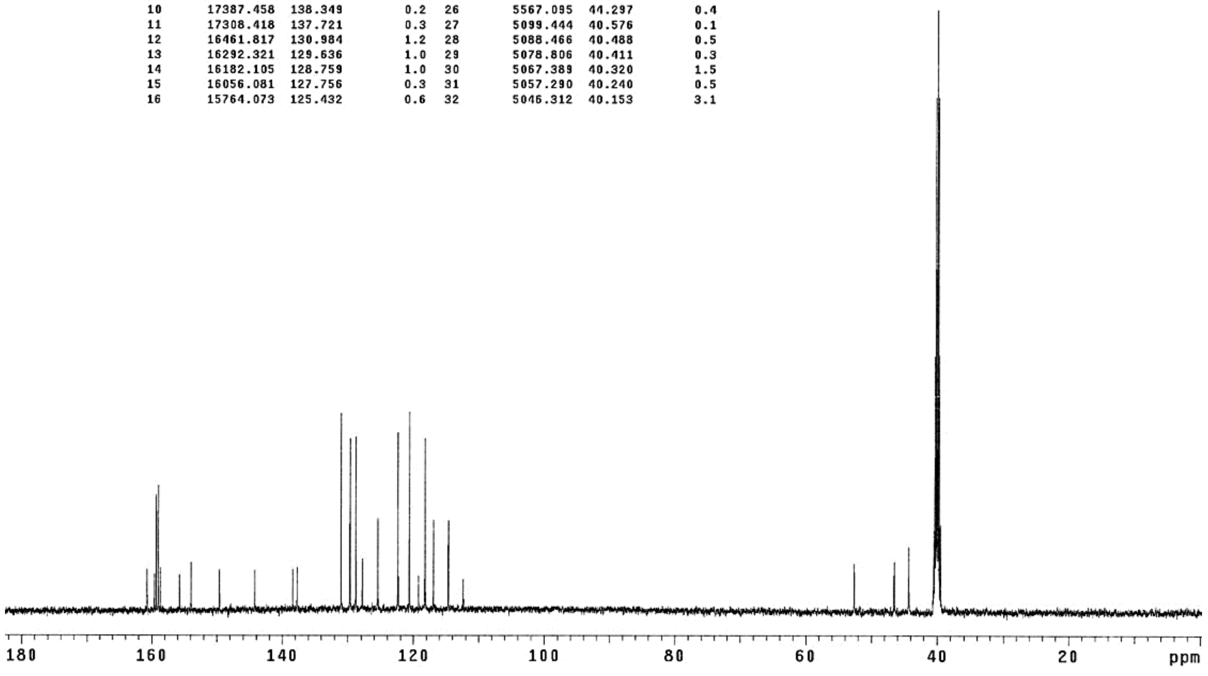

^{1}H-NMR (DMSO-d_6, 500 MHz) spectrum of *N*-(7-(4,5-dihydro-1*H*-imidazol-2-yl)-2-phenyl-6,7-dihydro-2*H*-imidazo[2,1-*c*][1,2,4]triazol-3(5*H*)-ylidene)-4-(trifluoromethyl)benzenesulfonamide (**5n**)

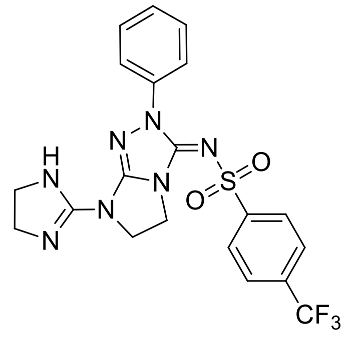

INDEX	FREQUENCY	PPM	HEIGHT	INDEX	FREQUENCY	PPM	HEIGHT
1	4035.053	8.073	13.0	17	2158.473	4.319	11.6
2	4026.752	8.057	15.7	18	2150.172	4.302	12.1
3	3965.224	7.934	17.3	19	2142.848	4.287	5.2
4	3956.923	7.917	13.2	20	1753.664	3.509	22.4
5	3892.954	7.789	13.4	21	1667.233	3.336	10.6
6	3891.978	7.787	14.2	22	1248.750	2.498	27.9
7	3884.165	7.771	16.1	23	1246.797	2.495	34.7
8	3728.882	7.461	9.7	24	1245.332	2.492	24.9
9	3721.557	7.446	16.9				
10	3713.256	7.429	9.7				
11	3658.565	7.320	5.1				
12	3651.240	7.305	8.1				
13	2209.746	4.421	6.2				
14	2202.421	4.407	13.1				
15	2199.492	4.401	8.8				
16	2193.632	4.389	11.7				

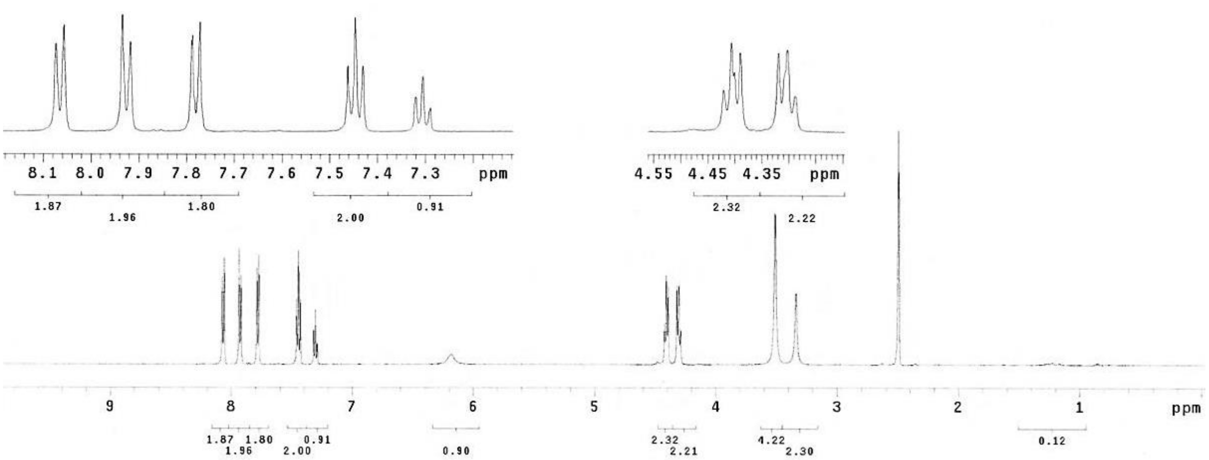

^{13}C-NMR (DMSO-d_6, 75 MHz) spectrum of *N*-(7-(4,5-dihydro-1*H*-imidazol-2-yl)-2-phenyl-6,7-dihydro-2*H*-imidazo[2,1-*c*][1,2,4]triazol-3(5*H*)-ylidene)-4-(trifluoromethyl)benzenesulfonamide (**5n**)

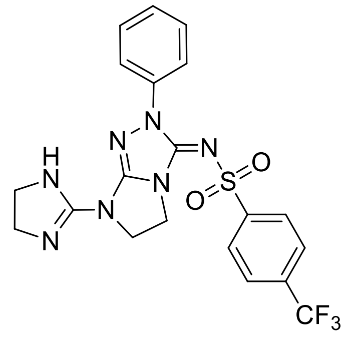

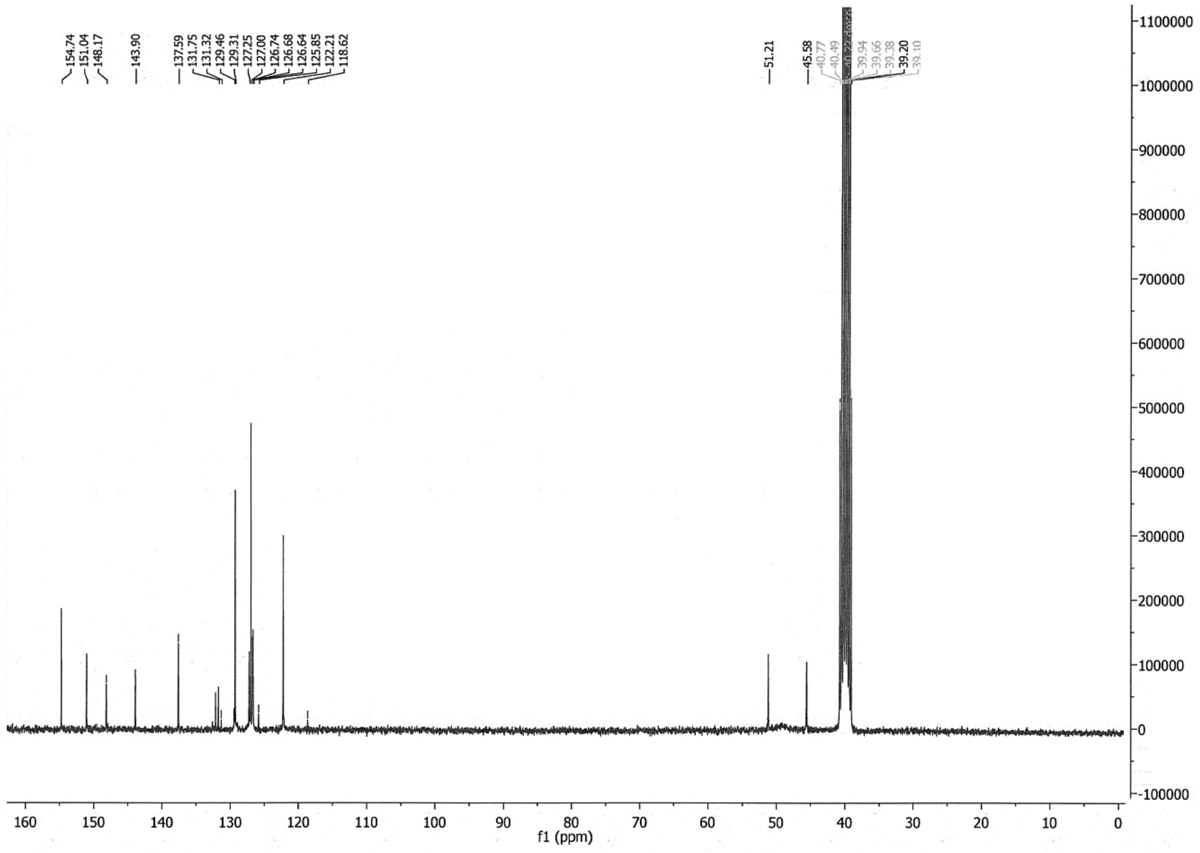

^{1}H-NMR (DMSO-d_6, 500 MHz) spectrum of 1-(7-(4,5-dihydro-1*H*-imidazol-2-yl)-2-phenyl-6,7-dihydro-2*H*-imidazo[2,1-*c*][1,2,4]triazol-3(5*H*)-ylidene)-3-phenylurea (**6a**)

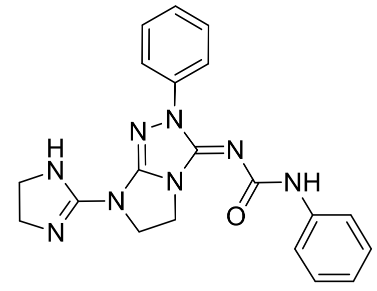

INDEX	FREQUENCY	PPM	HEIGHT	INDEX	FREQUENCY	PPM	HEIGHT	INDEX	FREQUENCY	PPM	HEIGHT
1	4583.489	9.170	5.0	14	3438.400	6.879	8.6	27	1243.930	2.489	12.5
2	4056.113	8.115	12.2	15	3431.075	6.865	4.1	28	1242.465	2.486	10.1
3	4048.300	8.100	12.4	16	3105.860	6.214	0.7				
4	3807.563	7.618	11.0	17	2175.627	4.353	5.5				
5	3799.750	7.602	11.7	18	2168.791	4.339	12.5				
6	3725.527	7.454	9.0	19	2160.490	4.323	12.3				
7	3717.225	7.437	15.5	20	2147.794	4.297	0.8				
8	3709.412	7.422	9.6	21	2131.679	4.265	11.7				
9	3616.633	7.236	5.1	22	2123.866	4.249	12.2				
10	3607.355	7.217	13.8	23	2116.053	4.234	5.4				
11	3599.542	7.202	16.9	24	1821.114	3.644	0.8				
12	3591.723	7.186	9.6	25	1758.121	3.518	34.3				
13	3445.724	6.894	4.8	26	1671.202	3.344	73.9				

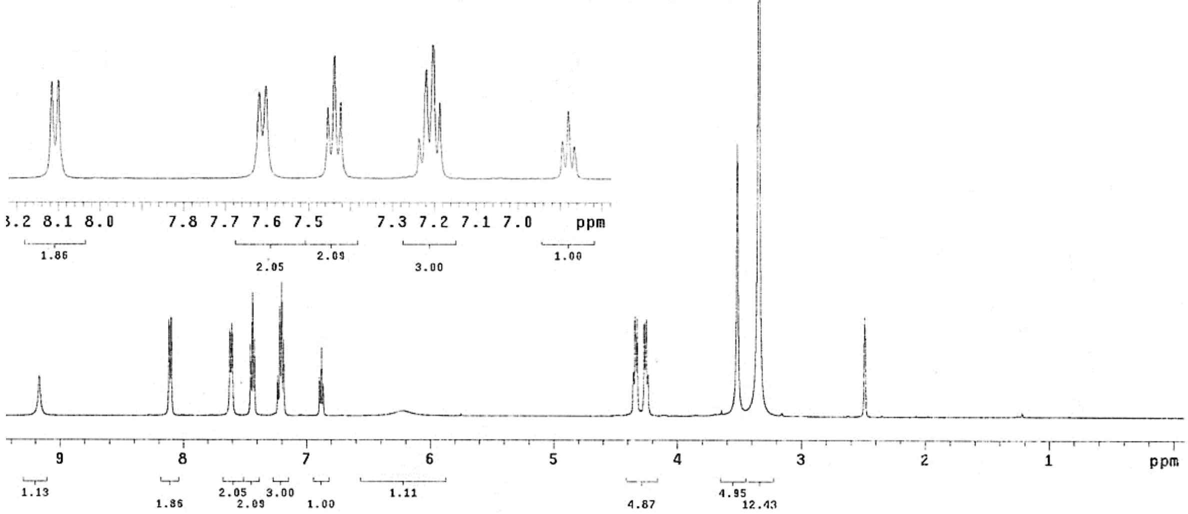

^{1}H-NMR (DMSO-d_6+TFA, 500 MHz) spectrum of 1-(7-(4,5-dihydro-1*H*-imidazol-2-yl)-2-phenyl-6,7-dihydro-2*H*-imidazo[2,1-*c*][1,2,4]triazol-3(5*H*)-ylidene)-3-phenylurea(**6a**)

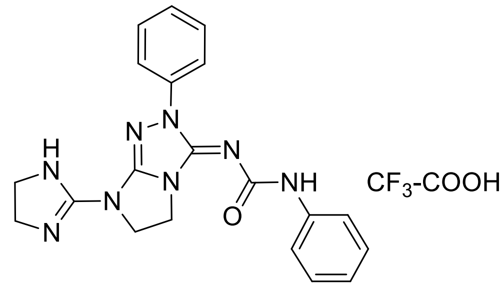

FREQUENCY	PPM	HEIGHT			
4655.271	9.314	4.3	3615.168	7.233	6.9
4599.603	9.203	3.9	3606.867	7.216	12.5
4090.295	8.184	10.7	3599.542	7.202	7.1
4081.994	8.167	10.9	3457.444	6.917	3.8
3810.981	7.625	8.8	3450.608	6.904	6.5
3803.656	7.610	9.2	3443.283	6.889	3.2
3745.059	7.493	6.1	2237.643	4.477	9.1
3737.246	7.477	11.4	2231.783	4.465	9.9
3729.433	7.462	6.8	2213.227	4.428	10.0
3643.979	7.291	3.7	2206.879	4.415	9.6
3637.142	7.277	6.1	1921.706	3.845	46.0
3629.818	7.262	3.1	1243.930	2.489	5.9

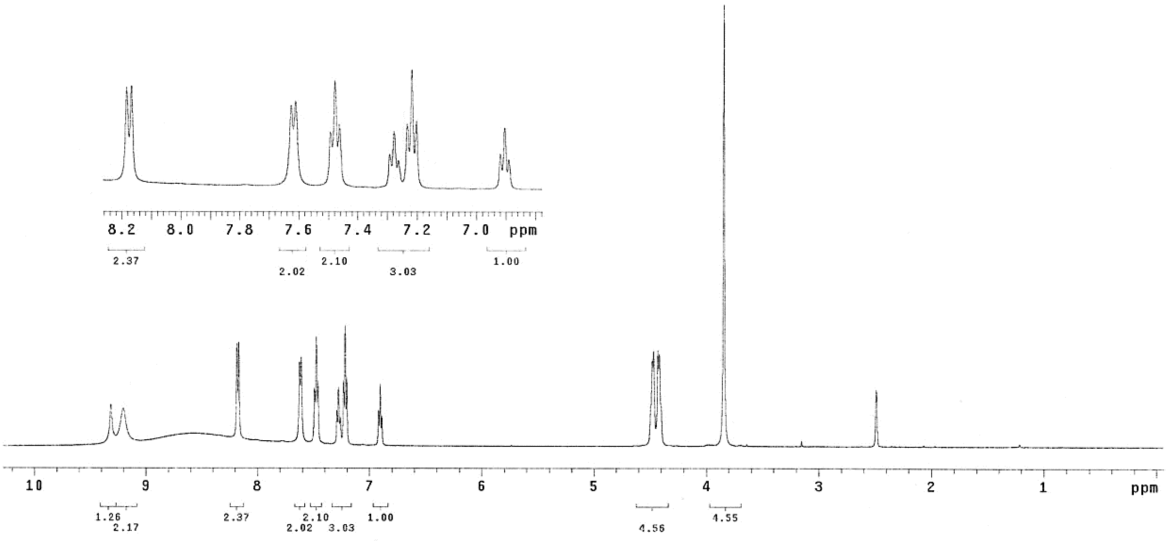

^{13}C-NMR (DMSO-d_6+TFA, 125 MHz) spectrum of 1-(7-(4,5-dihydro-1*H*-imidazol-2-yl)-2-phenyl-6,7-dihydro-2*H*-imidazo[2,1-*c*][1,2,4]triazol-3(5*H*)-ylidene)-3-phenylurea (**6a**)

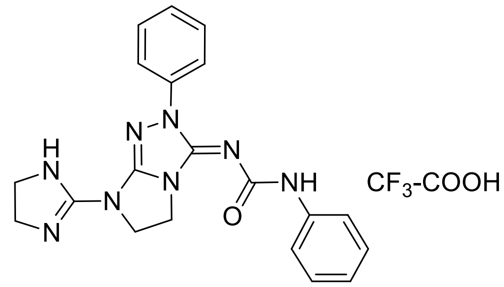

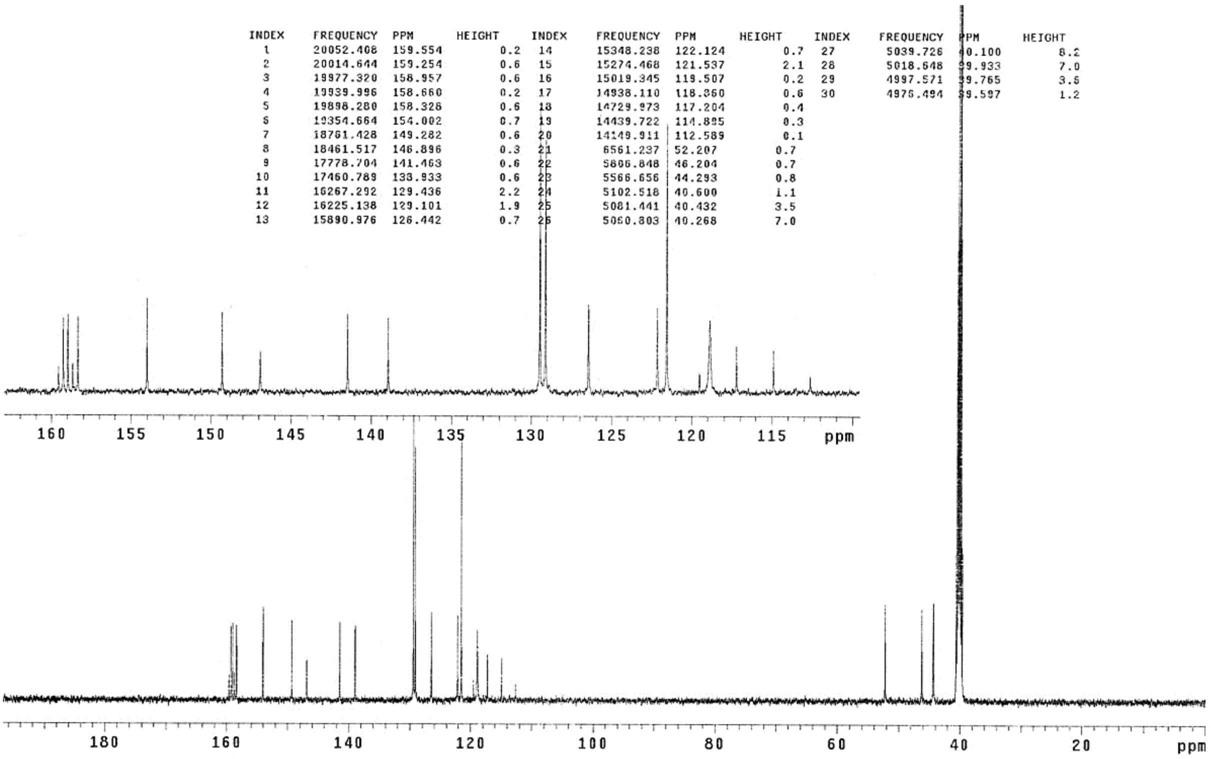

^{1}H-NMR (DMSO-d_6, 500 MHz) spectrum of 1-(7-(4,5-dihydro-1*H*-imidazol-2-yl)-2-(*p*-tolyl)-6,7-dihydro-2*H*-imidazo[2,1-*c*][1,2,4]triazol-3(5*H*)-ylidene)-3-phenylurea (**6b**)

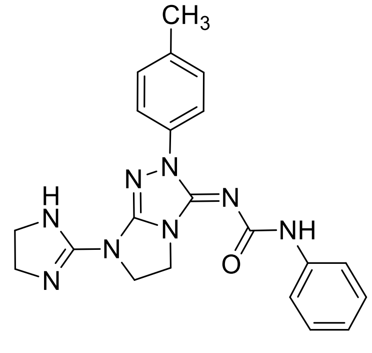

FREQUENCY	PPM	HEIGHT	FREQUENCY	PPM	HEIGHT
4558.097	9.120	14.3	2171.721	4.345	14.5
3980.425	7.964	36.4	2164.396	4.330	33.5
3972.124	7.947	38.5	2156.095	4.314	33 2
3804.145	7.611	30.4	2128.750	4.259	32.0
3795.843	7.595	32.8	2120.937	4.243	33 3
3621.028	7.245	42.4	2113.124	4.228	14.7
3612.727	7.228	40.5	1756.657	3.515	102.7
3603.937	7.211	25.0	1668.272	3.338	457.3
3596.124	7.195	43.0	1243.442	2.488	55.9
3587.823	7.178	24.7	1157.011	2.315	142.5
3441.818	6.886	12.9			
3434.493	6.872	22.8			
3427.169	6.857	11.1			

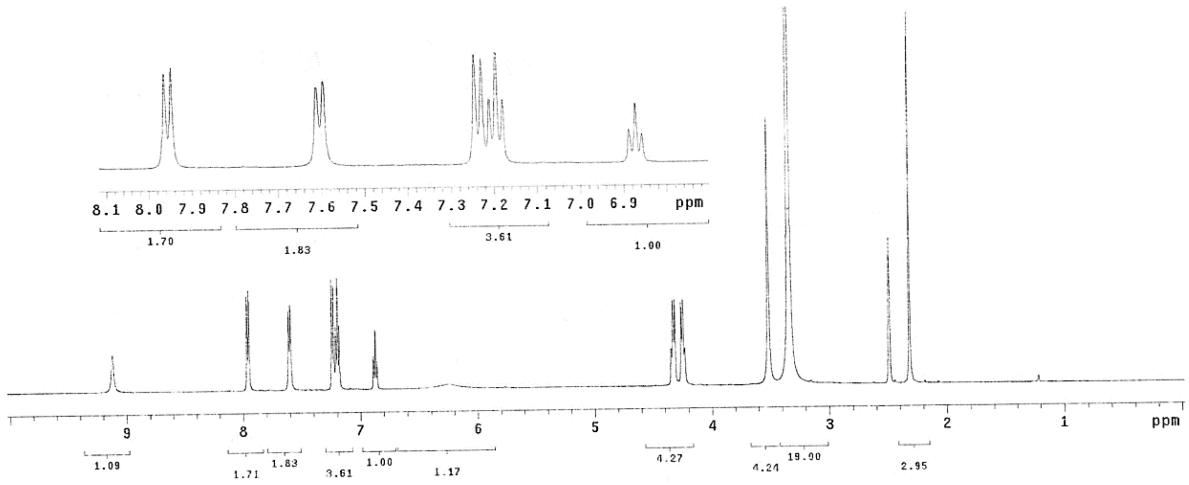

^{13}C-NMR (DMSO-d_6+TFA, 125 MHz) spectrum of 1-(7-(4,5-dihydro-1*H*-imidazol-2-yl)-2-(*p*-tolyl)-6,7-dihydro-2*H*-imidazo[2,1-*c*][1,2,4]triazol-3(5*H*)-ylidene)-3-phenylurea (**6b**)

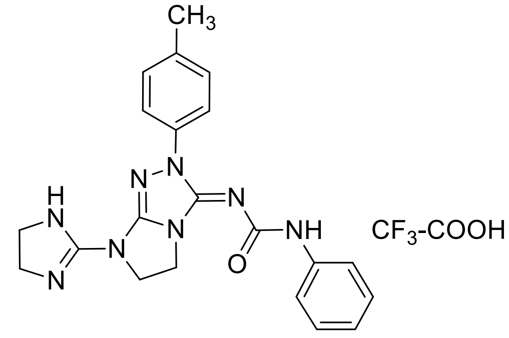

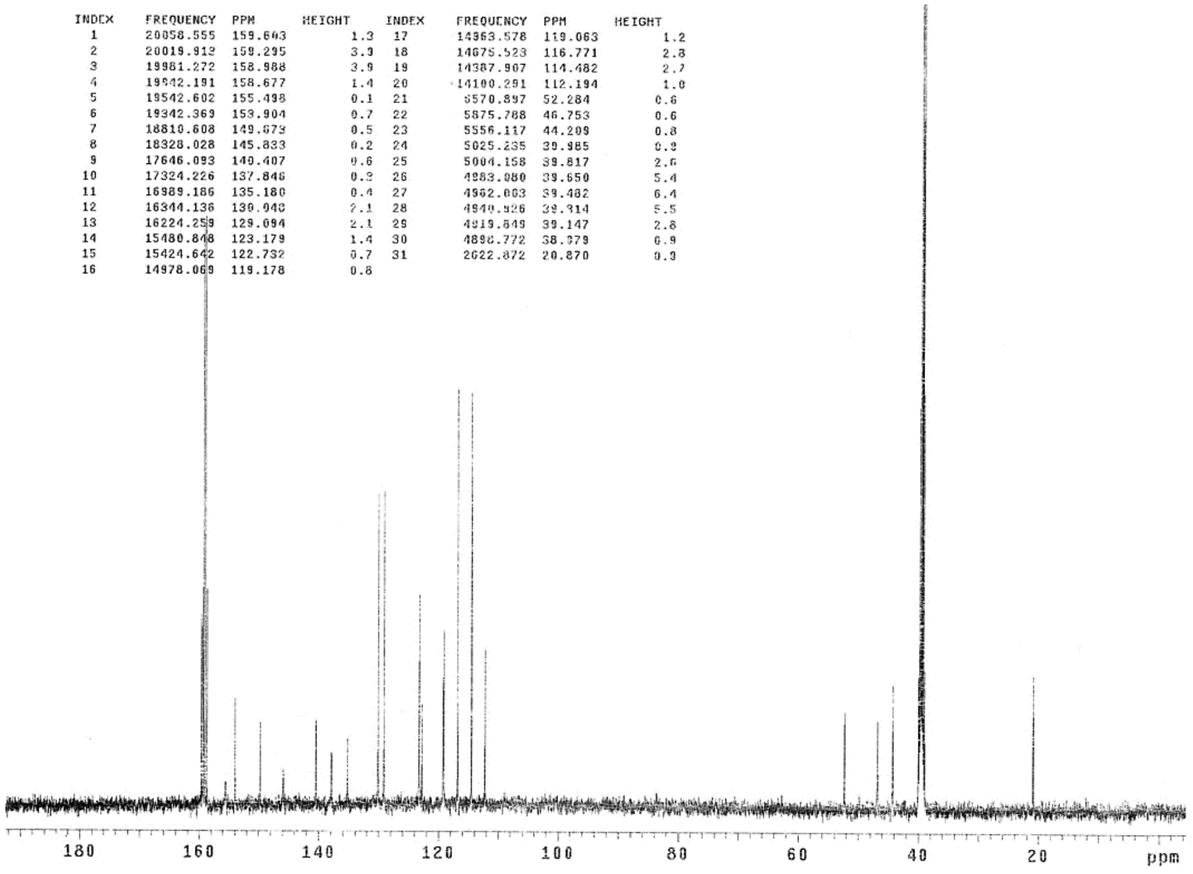

INDEX	FREQUENCY	PPM	HEIGHT	INDEX	FREQUENCY	PPM	HEIGHT
1	20058.555	159.603	1.3	17	14963.578	119.063	1.2
2	20019.912	159.295	3.3	18	14675.523	116.771	2.8
3	19981.272	158.988	3.9	19	14387.907	114.482	2.7
4	19942.191	158.677	1.4	20	14100.291	112.194	1.0
5	19542.602	155.498	0.1	21	6570.897	52.284	0.6
6	19342.369	153.904	0.7	22	5875.788	46.753	0.6
7	18810.608	149.673	0.5	23	5556.117	44.209	0.8
8	18328.028	145.833	0.2	24	5025.235	39.985	0.9
9	17646.093	140.407	0.6	25	5004.158	39.817	2.6
10	17324.226	137.846	0.3	26	4983.080	39.650	5.4
11	16989.186	135.180	0.4	27	4962.003	39.482	6.4
12	16344.136	130.048	2.1	28	4940.926	39.314	5.5
13	16224.259	129.094	2.1	29	4919.849	39.147	2.8
14	15480.848	123.179	1.4	30	4898.772	38.979	0.9
15	15424.642	122.732	0.7	31	2622.872	20.870	0.9
16	14978.069	119.178	0.8				

^{1}H-NMR (DMSO-d_6, 500 MHz) spectrum of 1-(7-(4,5-dihydro-1*H*-imidazol-2-yl)-2-phenyl-6,7-dihydro-2*H*-imidazo[2,1-*c*][1,2,4]triazol-3(5*H*)-ylidene)-3-(*p*-tolyl)urea (**6c**)

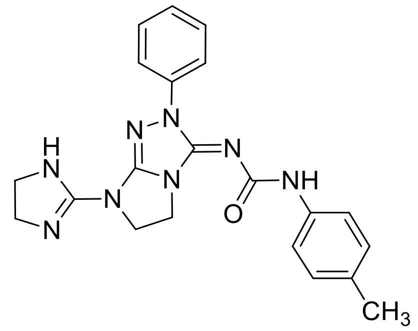

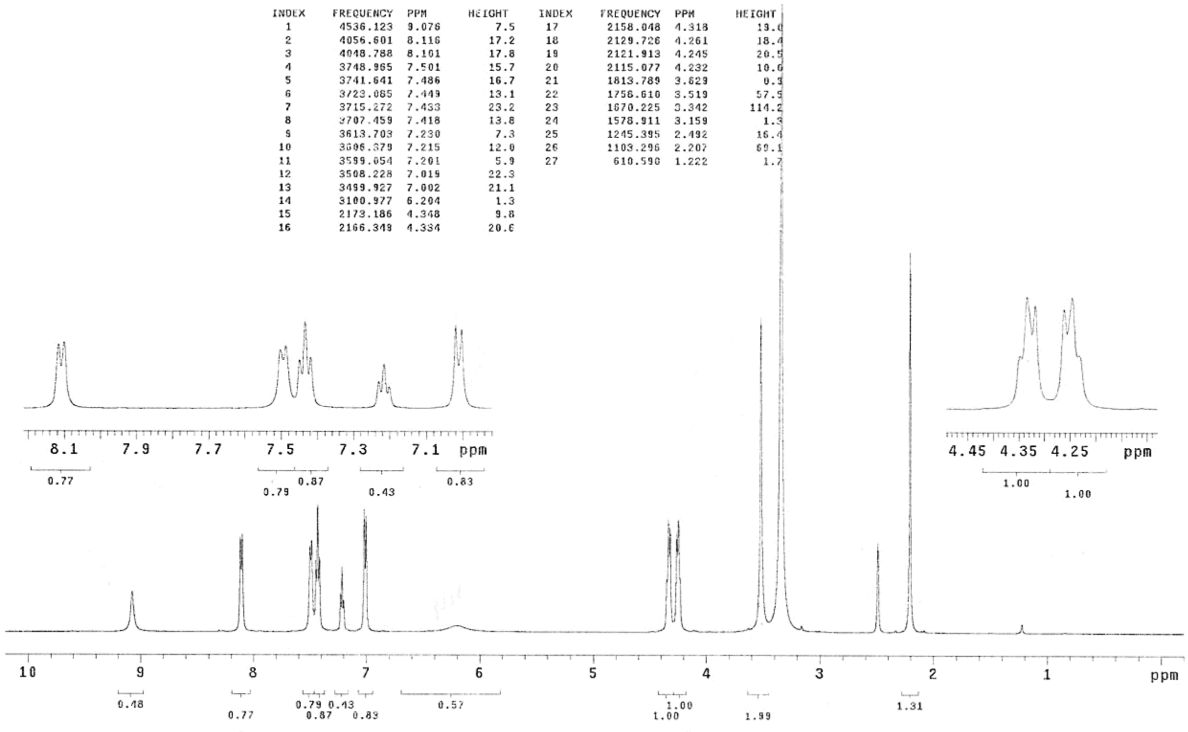

INDEX	FREQUENCY	PPM	HEIGHT	INDEX	FREQUENCY	PPM	HEIGHT
1	4536.123	9.076	7.5	17	2158.048	4.318	13.0
2	4056.601	8.116	17.2	18	2129.726	4.261	18.4
3	4048.788	8.101	17.8	19	2121.913	4.245	20.5
4	3748.965	7.501	15.7	20	2115.077	4.232	10.6
5	3741.641	7.486	16.7	21	1813.789	3.629	0.3
6	3723.085	7.449	13.1	22	1758.610	3.519	57.5
7	3715.272	7.433	23.2	23	1670.225	3.342	114.2
8	3707.459	7.418	13.8	24	1578.911	3.159	1.3
9	3613.703	7.230	7.3	25	1245.395	2.492	16.4
10	3606.379	7.215	12.0	26	1103.296	2.207	69.1
11	3599.054	7.201	5.9	27	610.590	1.222	1.7
12	3508.228	7.019	22.3				
13	3499.927	7.002	21.1				
14	3100.977	6.204	1.3				
15	2173.186	4.348	9.8				
16	2166.349	4.334	20.6				

^{13}C-NMR (DMSO-d_6+TFA, 125 MHz) spectrum of 1-(7-(4,5-dihydro-1*H*-imidazol-2-yl)-2-phenyl-6,7-dihydro-2*H*-imidazo[2,1-*c*][1,2,4]triazol-3(5*H*)-ylidene)-3-(*p*-tolyl)urea (**6c**)

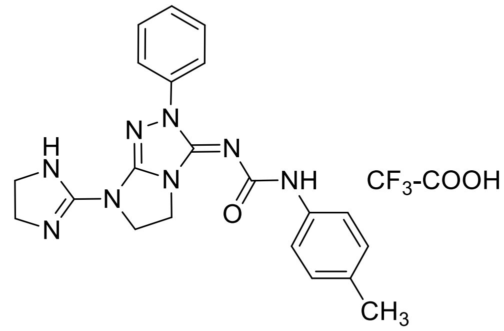

FREQUENCY	PPM	HEIGHT
20057.238	159.592	0.8
20018.157	159.282	2.6
19979.515	158.974	2.7
19940.435	158.663	0.9
19343.686	153.915	0.5
18815.000	149.708	0.3
18333.297	145.875	0.2
17324.226	137.846	0.4
17317.200	137.790	0.2
16560.177	131.767	0.4
16284.856	129.576	1.2
16273.000	129.482	1.3
16053.446	127.735	0.3
15451.428	122.945	0.8
14986.412	119.245	0.3
14960.505	119.039	0.6
14672.889	116.750	1.9
14385.273	114.461	1.9
14097.656	112.173	0.7
6568.701	52.266	0.4
5870.519	46.711	0.3
5554.361	44.195	0.5
5017.770	39.926	0.5
4996.693	39.758	1.6
4975.616	39.590	3.9
4954.538	39.423	3.9
4933.461	39.255	3.3
4912.384	39.087	1.7
4891.307	38.919	0.6
2585.548	20.573	0.5

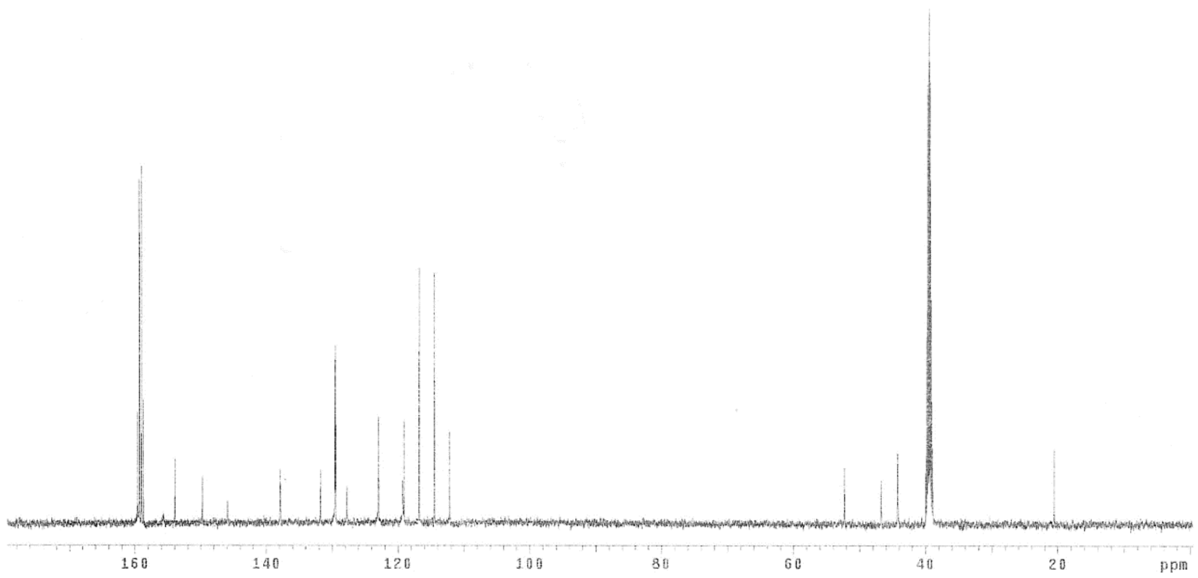

^{1}H-NMR (DMSO-d_6, 500 MHz) spectrum of 1-(7-(4,5-dihydro-1*H*-imidazol-2-yl)-2-(*p*-tolyl)-6,7-dihydro-2*H*-imidazo[2,1-*c*][1,2,4]triazol-3(5*H*)-ylidene)-3-(*p*-tolyl)urea (**6d**)

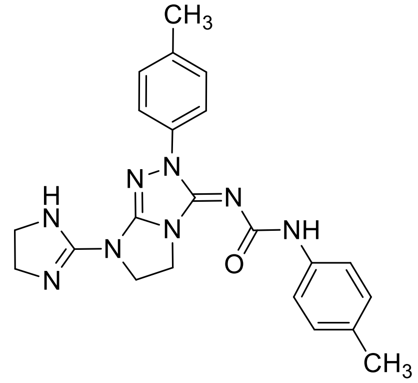

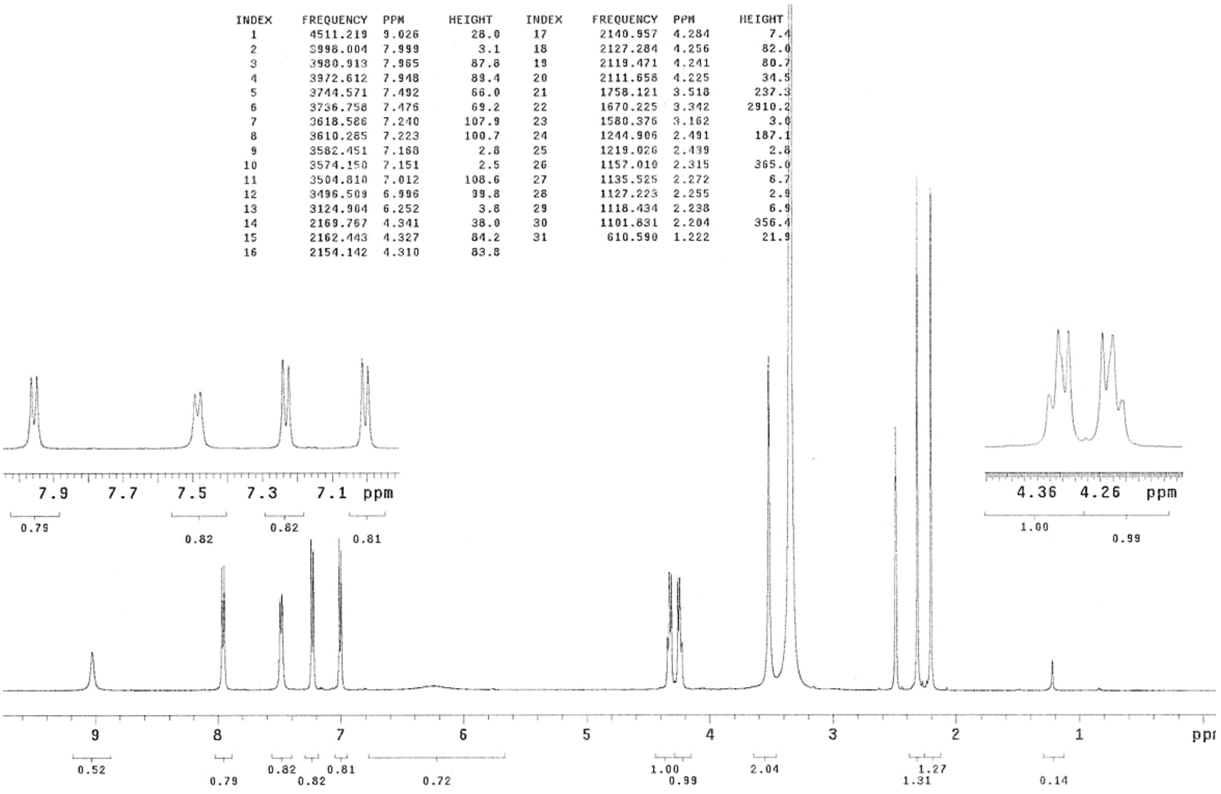

^{13}C-NMR (DMSO-d_6+TFA, 125 MHz) spectrum of 1-(7-(4,5-dihydro-1*H*-imidazol-2-yl)-2-(*p*-tolyl)-6,7-dihydro-2*H*-imidazo[2,1-*c*][1,2,4]triazol-3(5*H*)-ylidene)-3-(*p*-tolyl)urea (**6d**)

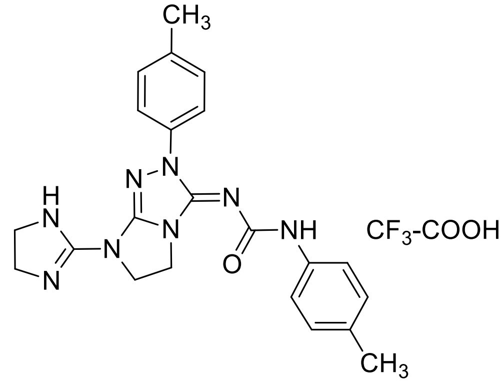

FREQUENCY	PPM	HEIGHT
20054.164	159.568	2.8
20015.083	159.257	9.1
19976.002	158.946	9.4
19937.361	158.638	3.4
19342.369	153.904	0.7
18852.763	150.009	0.3
18254.726	145.251	0.2
17443.224	138.793	0.2
17249.138	137.249	0.3
16906.195	134.520	0.2
16620.335	132.246	0.4
16370.043	130.254	1.3
16286.173	129.587	1.5
15573.939	123.920	0.6
15003.976	119.384	0.7
14956.114	119.004	2.7
14668.498	116.715	7.0
14380.881	114.427	6.9
14093.265	112.138	2.6
6584.070	52.388	0.4
5921.455	47.116	0.3
5558.752	44.230	0.6
5017.331	39.922	1.9
4996.254	39.754	5.8
4975.176	39.587	11.4
4954.099	39.419	13.3
4933.022	39.251	11.4
4911.945	39.084	5.7
4890.868	38.916	1.8
2624.189	20.880	0.6
2586.426	20.530	0.5

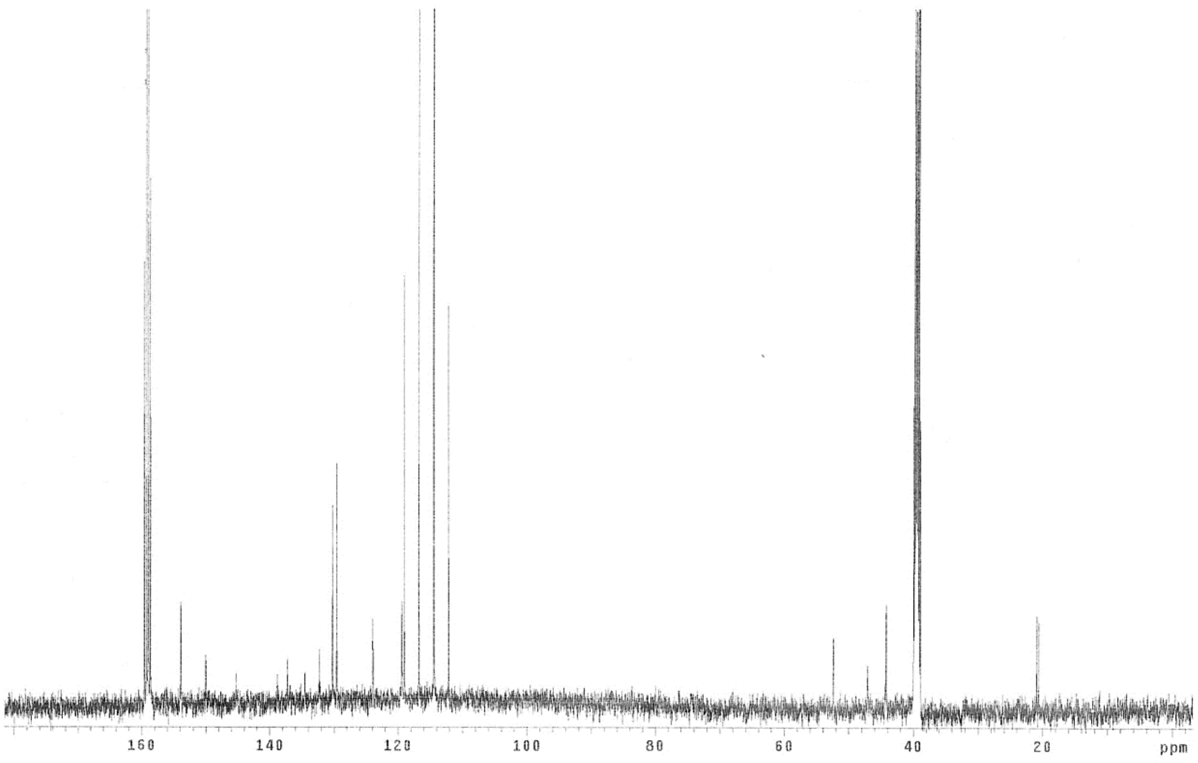

^{1}H-NMR (DMSO-*d*6, 300 MHz) spectrum of 1-(4-chlorophenyl)-3-(7-(4,5-dihydro-1*H*-imidazol-2-yl)-2-(*p*-tolyl)-6,7-dihydro-2*H*-imidazo[2,1-*c*][1,2,4]triazol-3(5*H*)-ylidene)urea (**6e**)

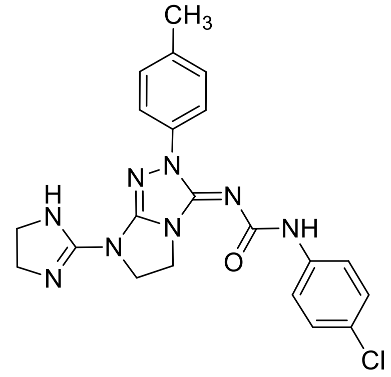

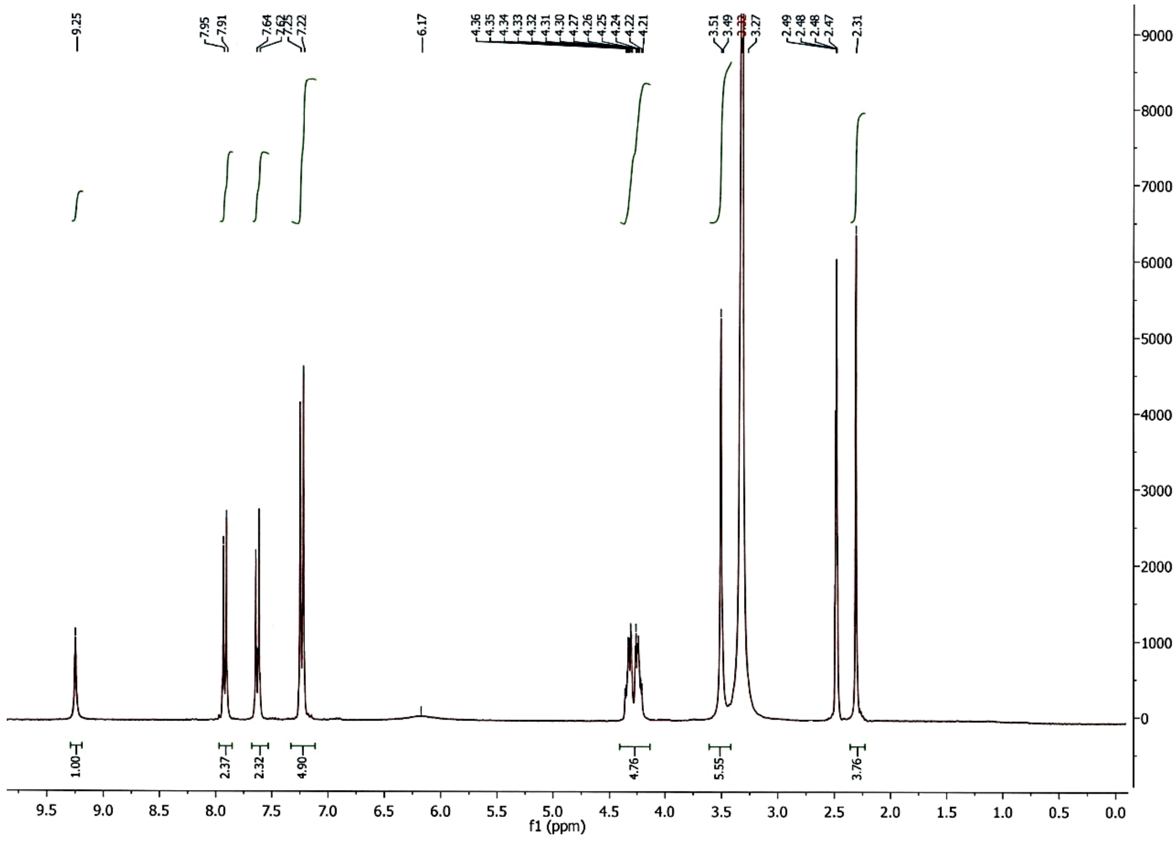

^{13}C-NMR (DMSO-d_6, 75 MHz) spectrum of 1-(4-chlorophenyl)-3-(7-(4,5-dihydro-1*H*-imidazol-2-yl)-2-(*p*-tolyl)-6,7-dihydro-2*H*-imidazo[2,1-*c*][1,2,4]triazol-3(5*H*)-ylidene)urea (**6e**)

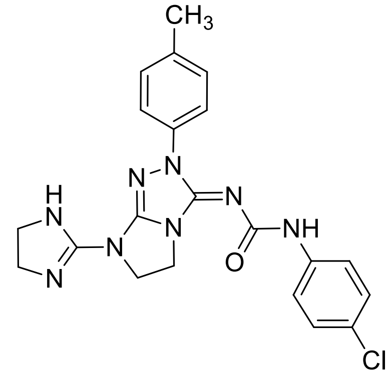

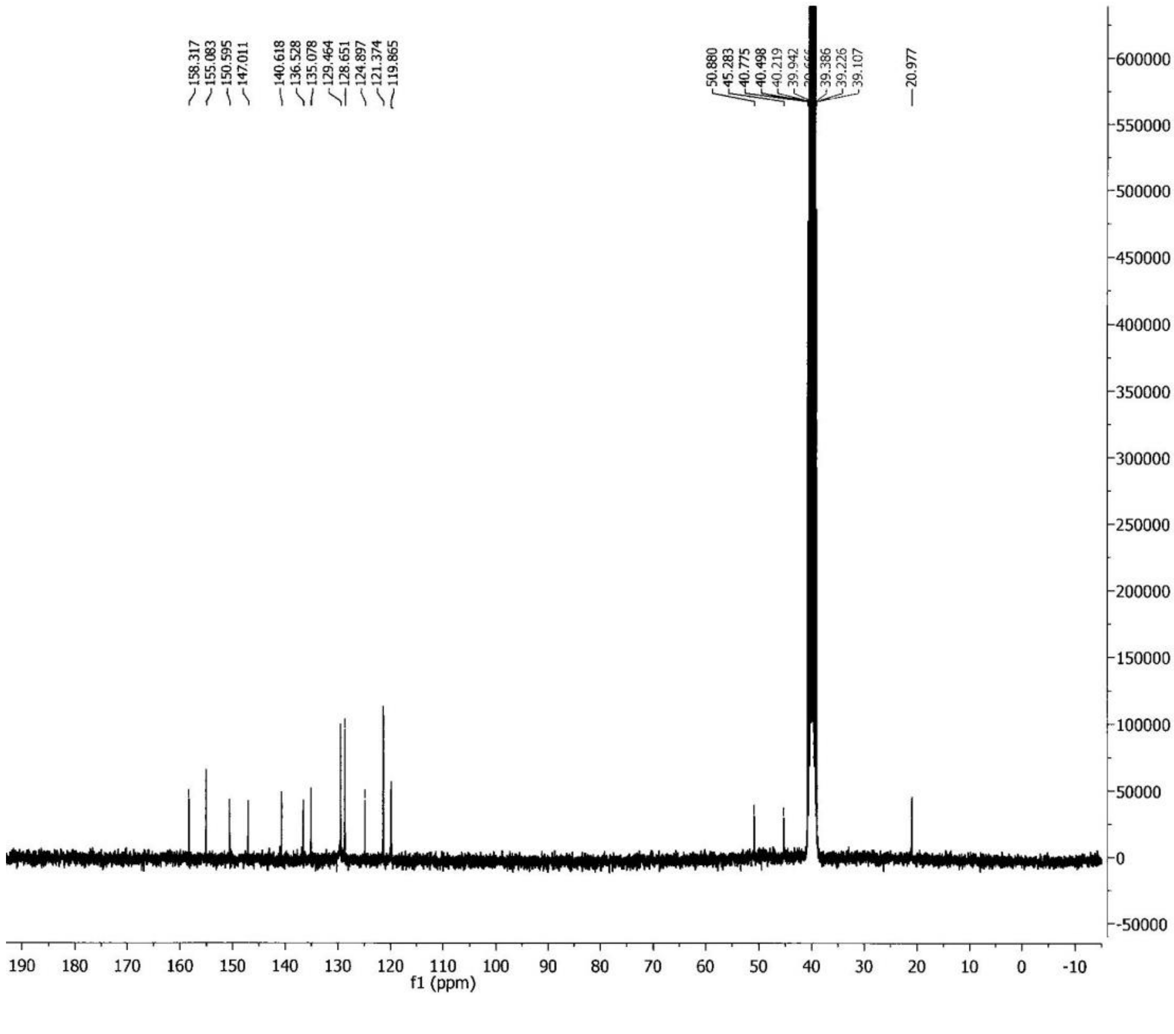

^{1}H-NMR (DMSO-d_6, 300 MHz) spectrum of 1-(4-chlorophenyl)-3-(2-(4-chlorophenyl)-7-(4,5-dihydro-1*H*-imidazol-2-yl)-6,7-dihydro-2*H*-imidazo[2,1-*c*][1,2,4]triazol-3(5*H*)-ylidene)urea (**6f**)

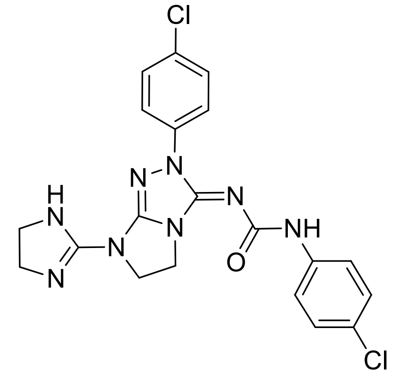

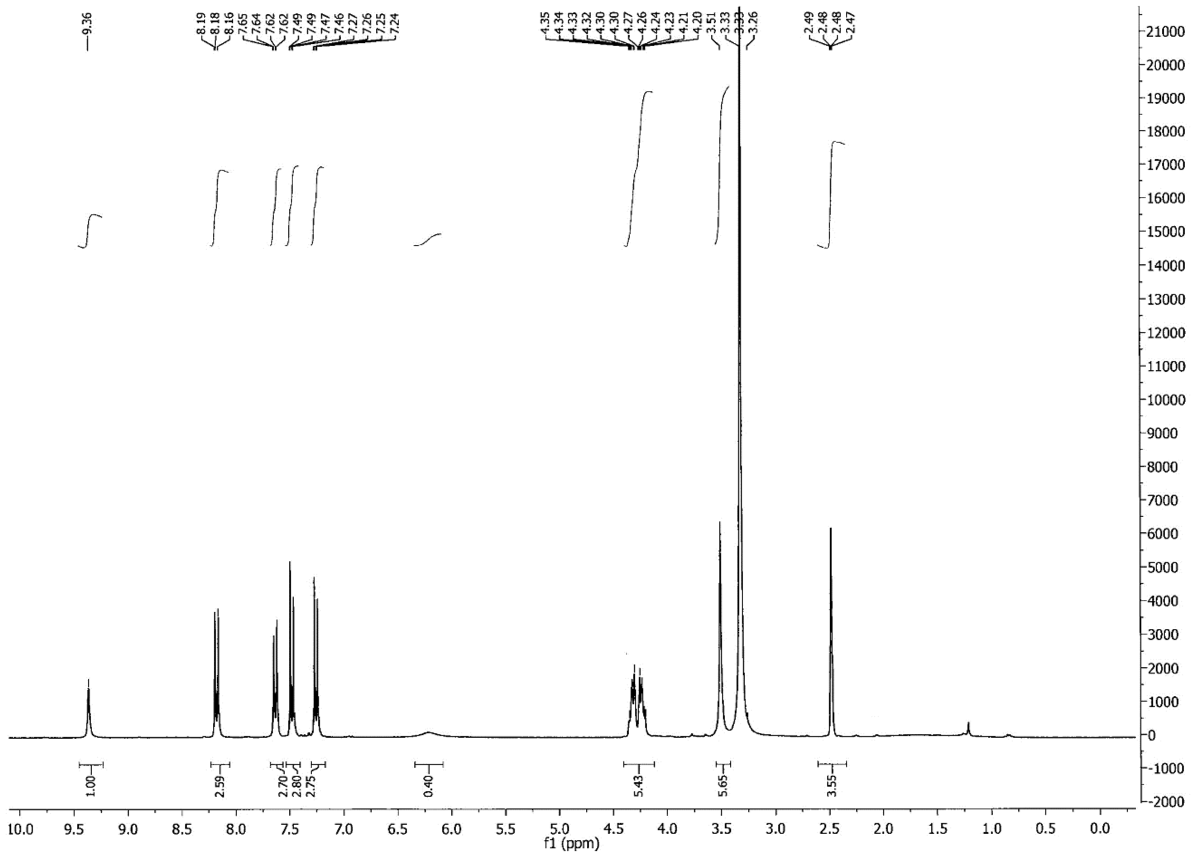

^{13}C-NMR (DMSO-*d*$_6$, 75 MHz) spectrum of 1-(4-chlorophenyl)-3-(2-(4-chlorophenyl)-7-(4,5-dihydro-1*H*-imidazol-2-yl)-6,7-dihydro-2*H*-imidazo[2,1-*c*][1,2,4]triazol-3(5*H*)-ylidene)urea (**6f**)

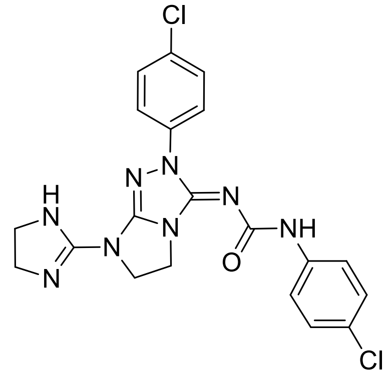

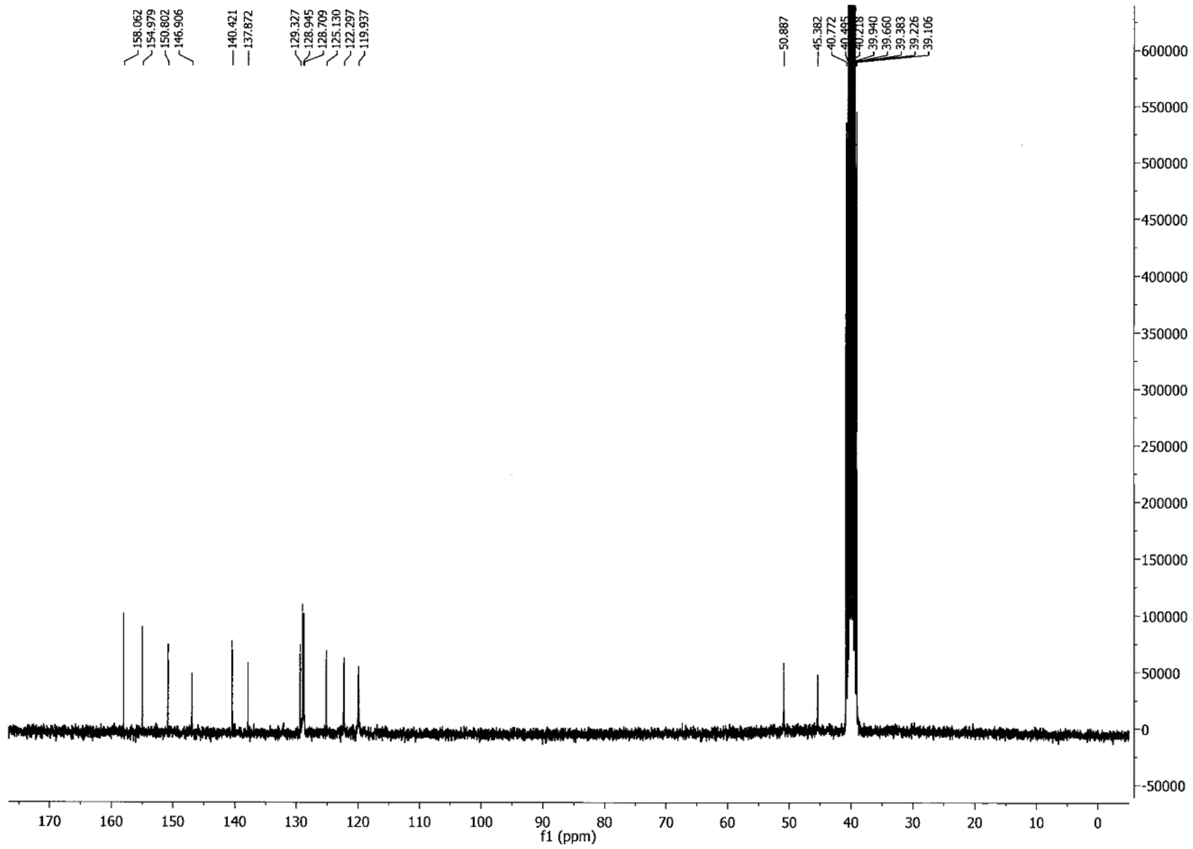

^{1}H-NMR (DMSO-d_6, 500 MHz) spectrum of 1-(7-(4,5-dihydro-1*H*-imidazol-2-yl)-2-phenyl-6,7-dihydro-2*H*-imidazo[2,1-*c*][1,2,4]triazol-3(5*H*)-ylidene)-3-(naphthalen-1-yl)urea (**6g**) recorded at a temperature of 70°C

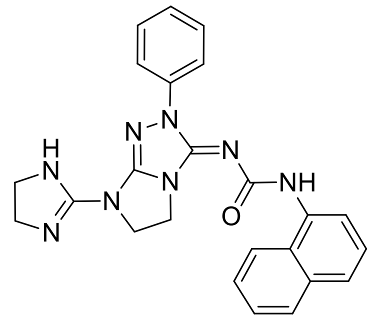

FREQUENCY	PPM	HEIGHT
4407.209	8.818	28.4
4072.716	8.148	16.4
4069.786	8.143	16.1
4063.438	8.130	17.6
4038.534	8.080	31.0
4030.721	8.064	31.4
3942.825	7.889	15.8
3940.383	7.884	12.1
3938.918	7.881	12.4
3936.477	7.876	17.3
3933.547	7.870	17.3
3929.641	7.862	3.7
3882.274	7.767	22.0
3874.950	7.753	23.3
3826.119	7.655	23.3
3818.306	7.639	26.9
3746.036	7.495	40.9
3742.817	7.488	39.1
3739.688	7.482	31.2
3736.758	7.476	39.1
3732.851	7.469	8.8
3729.921	7.463	22.9
3721.620	7.446	33.1
3713.807	7.430	16.4
3696.228	7.395	22.1
3688.415	7.380	39.7
3680.602	7.364	23.5
3602.472	7.208	14.1
3595.147	7.193	23.0
3587.823	7.176	9.9
2197.601	4.397	19.9
2190.277	4.382	43.2
2181.975	4.366	38.2
2142.910	4.287	38.4
2134.609	4.271	42.5
2127.284	4.256	19.1
1773.259	3.548	246.1
1763.493	3.528	8.0
1582.818	3.167	17.1
1245.395	2.492	14.2
1243.930	2.489	18.3
1241.976	2.485	14.2

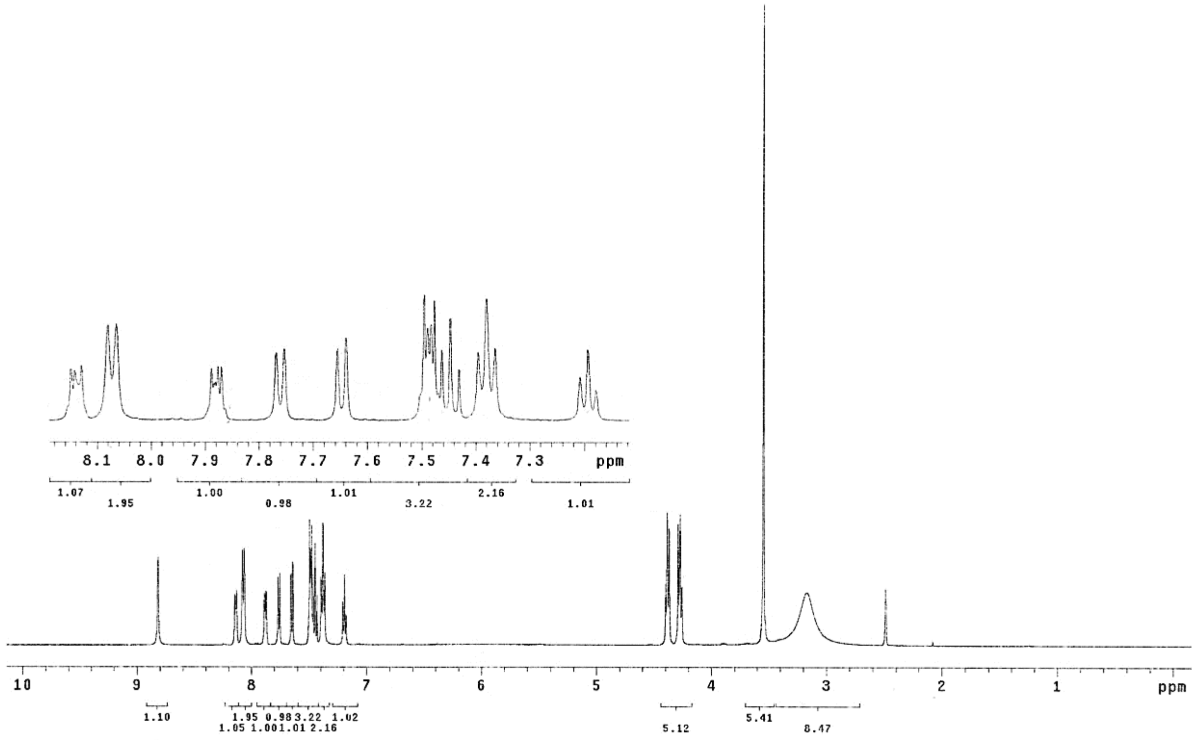

^{1}H-NMR (DMSO-d_6+TFA, 500 MHz) spectrum of 1-(7-(4,5-dihydro-1*H*-imidazol-2-yl)-2-phenyl-6,7-dihydro-2*H*-imidazo[2,1-*c*][1,2,4]triazol-3(5*H*)-ylidene)-3-(naphthalen-1-yl)urea (**6g**)

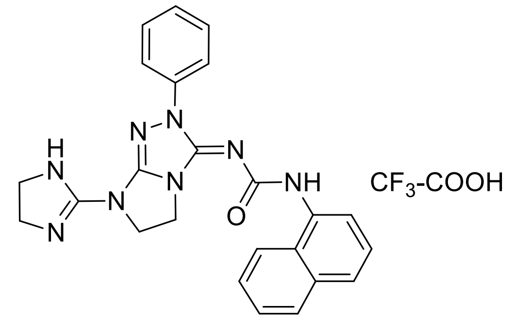

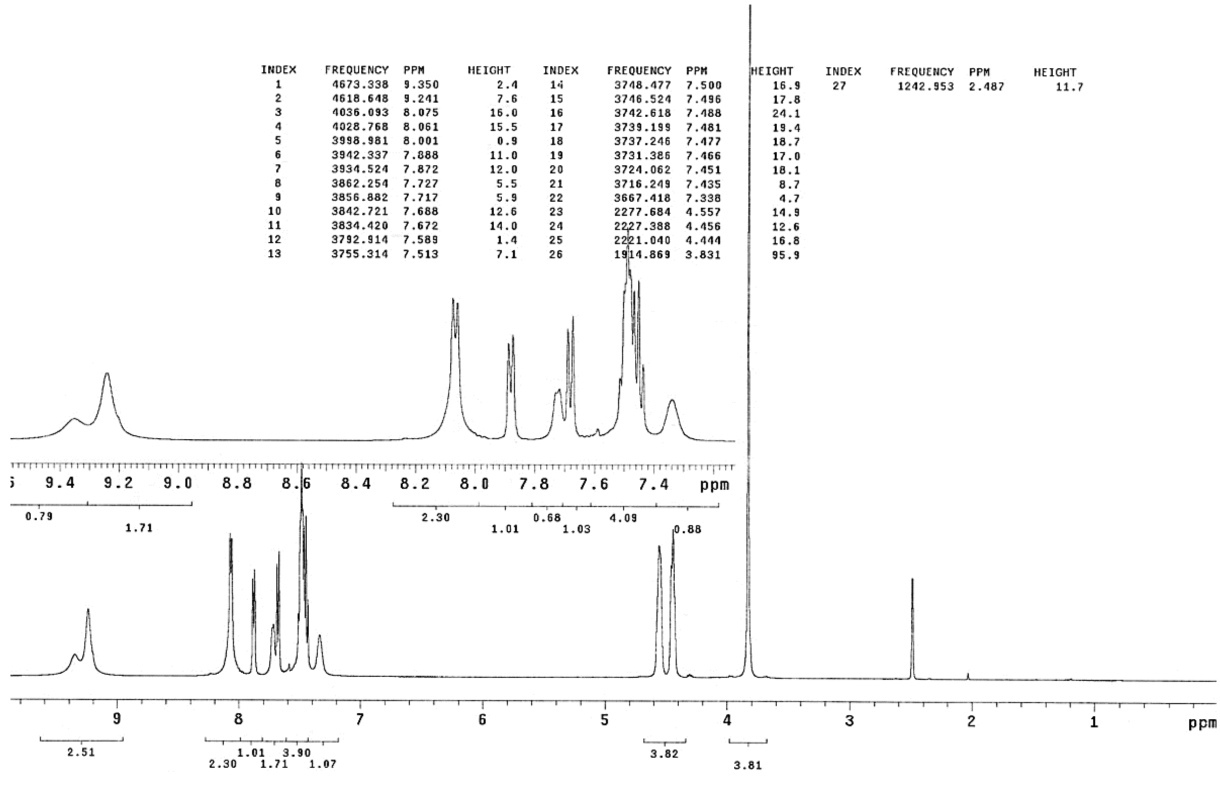

^{13}C-NMR (DMSO-d_6+TFA, 125 MHz) spectrum of 1-(7-(4,5-dihydro-1*H*-imidazol-2-yl)-2-phenyl-6,7-dihydro-2*H*-imidazo[2,1-*c*][1,2,4]triazol-3(5*H*)-ylidene)-3-(naphthalen-1-yl)urea (**6g**)

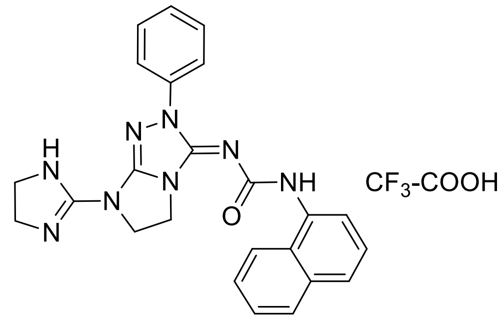

INDEX	FREQUENCY	PPM	HEIGHT	INDEX	FREQUENCY	PPM	HEIGHT	INDEX	FREQUENCY	PPM	HEIGHT
1	20058.555	159.603	2.7	17	16060.911	127.794	0.2	33	5022.161	39.961	2.2
2	20019.913	159.295	8.6	18	15898.441	126.502	1.4	34	5001.084	39.793	6.4
3	19981.272	158.988	9.0	19	15873.411	126.302	1.6	35	4980.007	39.625	12.8
4	19942.630	158.681	3.1	20	15843.113	126.061	1.6	36	4958.929	39.457	15.1
5	19338.417	153.873	1.1	21	15786.468	125.611	0.4	37	4937.852	39.290	12.9
6	18849.689	149.984	0.5	22	15747.826	125.303	0.7	38	4916.775	39.122	6.5
7	18294.217	145.564	0.3	23	15508.073	123.395	0.3	39	4895.698	38.954	2.1
8	17258.360	137.322	0.4	24	15471.188	123.102	0.6				
9	16909.708	134.548	0.2	25	15271.833	121.516	0.3				
10	16885.557	134.356	1.1	26	14963.139	119.059	2.4				
11	16396.829	130.467	0.4	27	14675.523	116.771	6.4				
12	16311.203	129.786	1.2	28	14387.907	114.482	6.3				
13	16251.484	129.311	0.2	29	14099.852	112.190	2.3				
14	16221.185	129.070	0.2	30	6581.435	52.368	1.0				
15	16172.005	128.678	1.1	31	5903.013	46.969	0.7				
16	16130.729	128.350	0.2	32	5558.752	44.230	1.3				

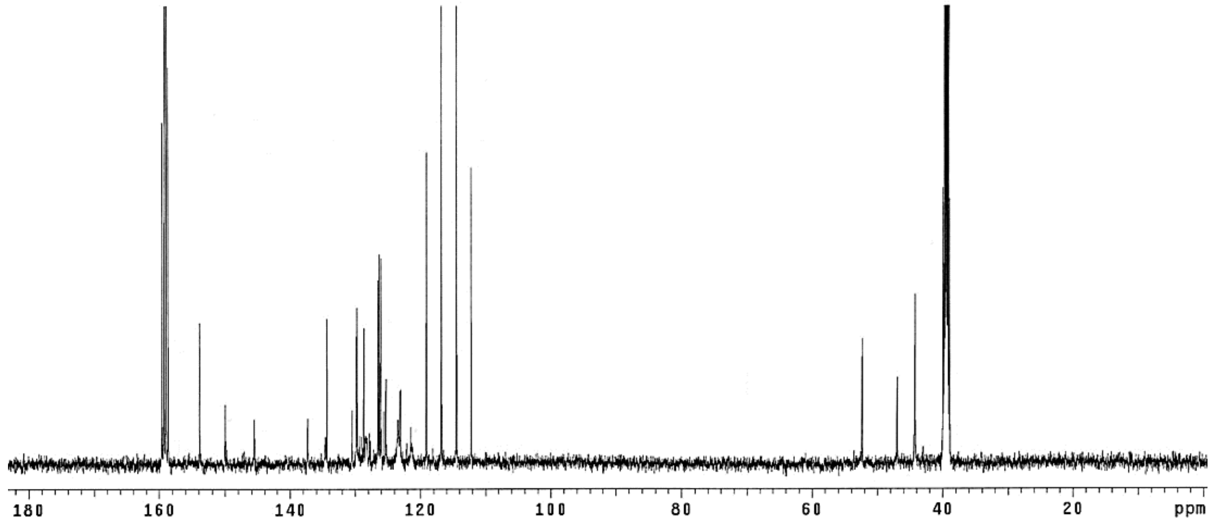

^{1}H-NMR (DMSO-d_6, 500 MHz) spectrum of *N*-((7-(4,5-dihydro-1*H*-imidazol-2-yl)-2-phenyl-6,7-dihydro-2*H*-imidazo[2,1-*c*][1,2,4]triazol-3(5*H*)-ylidene)carbamoyl)-4-methylbenzenesulfonamide (**6h**)

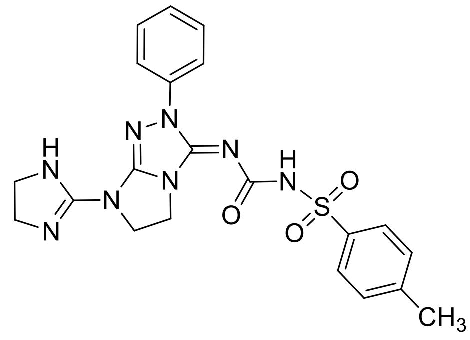

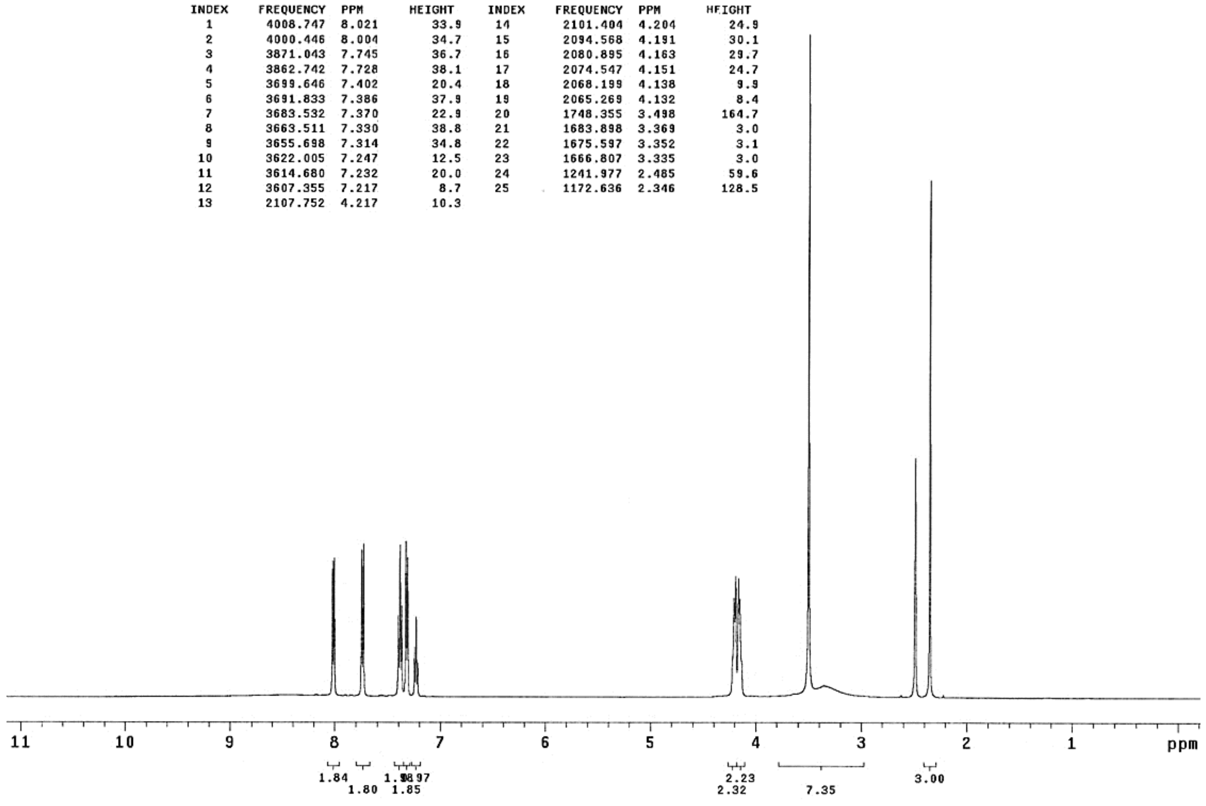

^{1}H-NMR (DMSO-d_6+TFA, 500 MHz) spectrum of *N*-((7-(4,5-dihydro-1*H*-imidazol-2-yl)-2-phenyl-6,7-dihydro-2*H*-imidazo[2,1-*c*][1,2,4]triazol-3(5*H*)-ylidene)carbamoyl)-4-methylbenzenesulfonamide (**6h**)

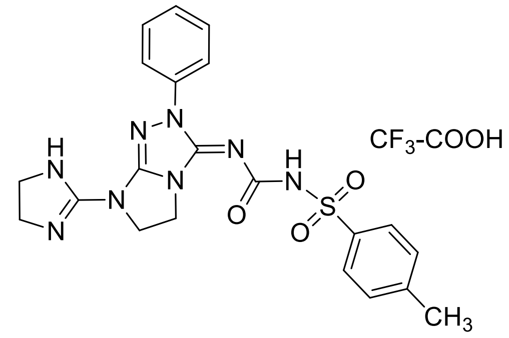

INDEX	FREQUENCY	PPM	HEIGHT	INDEX	FREQUENCY	PPM	HEIGHT
1	4018.513	8.040	10.1				
2	4010.212	8.023	10.7				
3	3866.160	7.735	11.3				
4	3857.859	7.719	11.8				
5	3693.298	7.389	6.0				
6	3685.485	7.374	11.5				
7	3677.672	7.358	6.7				
8	3632.259	7.267	12.1				
9	3623.958	7.251	13.9				
10	3615.168	7.233	6.3				
11	2172.698	4.347	13.1				
12	2167.326	4.336	12.8				
13	2162.931	4.327	8.3				
14	1894.849	3.791	47.5				
15	1241.977	2.485	22.7				
16	1147.244	2.295	39.6				

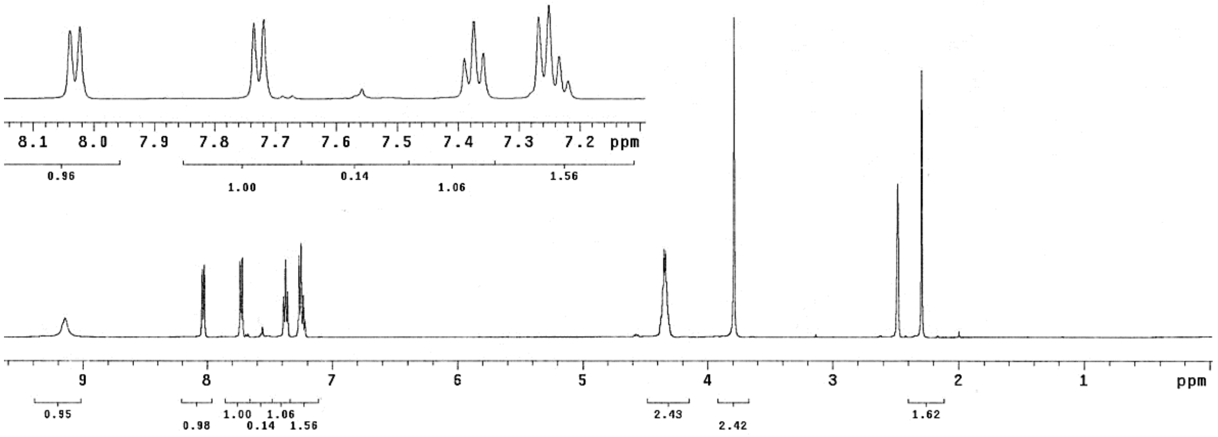

^{13}C-NMR (DMSO-d_6+TFA, 125 MHz) spectrum of *N*-((7-(4,5-dihydro-1*H*-imidazol-2-yl)-2-phenyl-6,7-dihydro-2*H*-imidazo[2,1-*c*][1,2,4]triazol-3(5*H*)-ylidene)carbamoyl)-4-methylbenzenesulfonamide (**6h**)

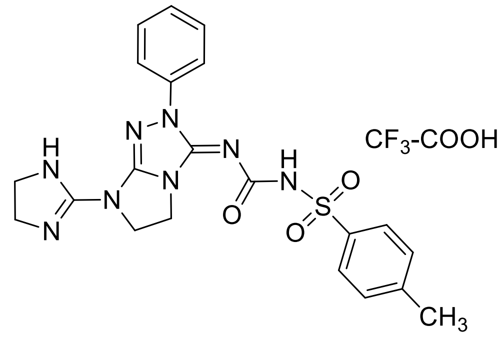

INDEX	FREQUENCY	PPM	HEIGHT	INDEX	FREQUENCY	PPM	HEIGHT	INDEX	FREQUENCY	PPM	HEIGHT
1	20052.846	159.558	0.9	17	14953.479	118.983	0.9	33	4880.329	38.832	0.5
2	20014.205	159.250	2.8	18	14665.863	116.694	2.2	34	2661.953	21.181	0.4
3	19975.124	158.939	3.0	19	14378.247	114.406	2.2				
4	19936.044	158.628	1.1	20	14091.070	112.121	0.8				
5	19582.122	155.812	0.3	21	6551.576	52.130	0.3				
6	19340.612	153.890	0.3	22	5825.291	46.351	0.3				
7	18757.915	149.254	0.3	23	5545.139	44.122	0.3				
8	18552.413	147.619	0.2	24	5006.792	39.838	0.6				
9	18067.637	143.762	0.3	25	4996.693	39.758	0.3				
10	17397.118	138.426	0.3	26	4985.715	39.671	1.6				
11	17369.893	138.210	0.3	27	4975.616	39.590	0.6				
12	16298.029	129.681	0.8	28	4964.638	39.503	3.2				
13	16233.041	129.164	0.9	29	4954.538	39.423	0.5				
14	16061.350	127.798	0.8	30	4943.561	39.335	3.8				
15	15949.816	126.910	0.3	31	4922.483	39.167	3.2				
		22.026	0.9	32	4901.406	39.000	1.5				

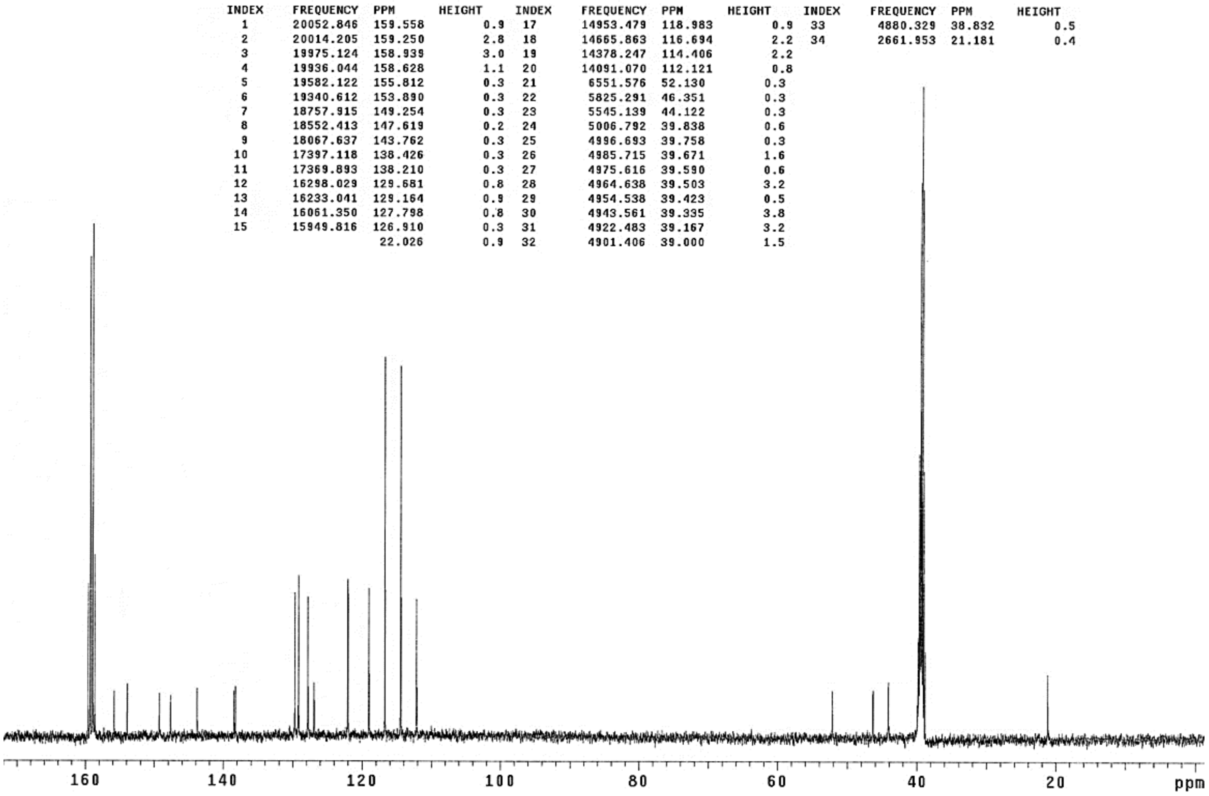

^{1}H-NMR (DMSO-d_6, 500 MHz) spectrum of *N*-((7-(4,5-dihydro-1*H*-imidazol-2-yl)-2-(*p*-tolyl)-6,7-dihydro-2*H*-imidazo[2,1-*c*][1,2,4]triazol-3(5*H*)-ylidene)carbamoyl)-4-methylbenzenesulfonamide (**6i**)

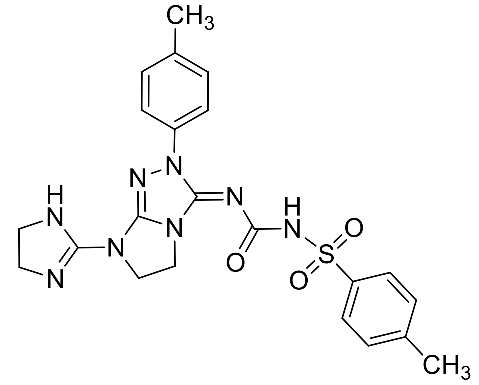

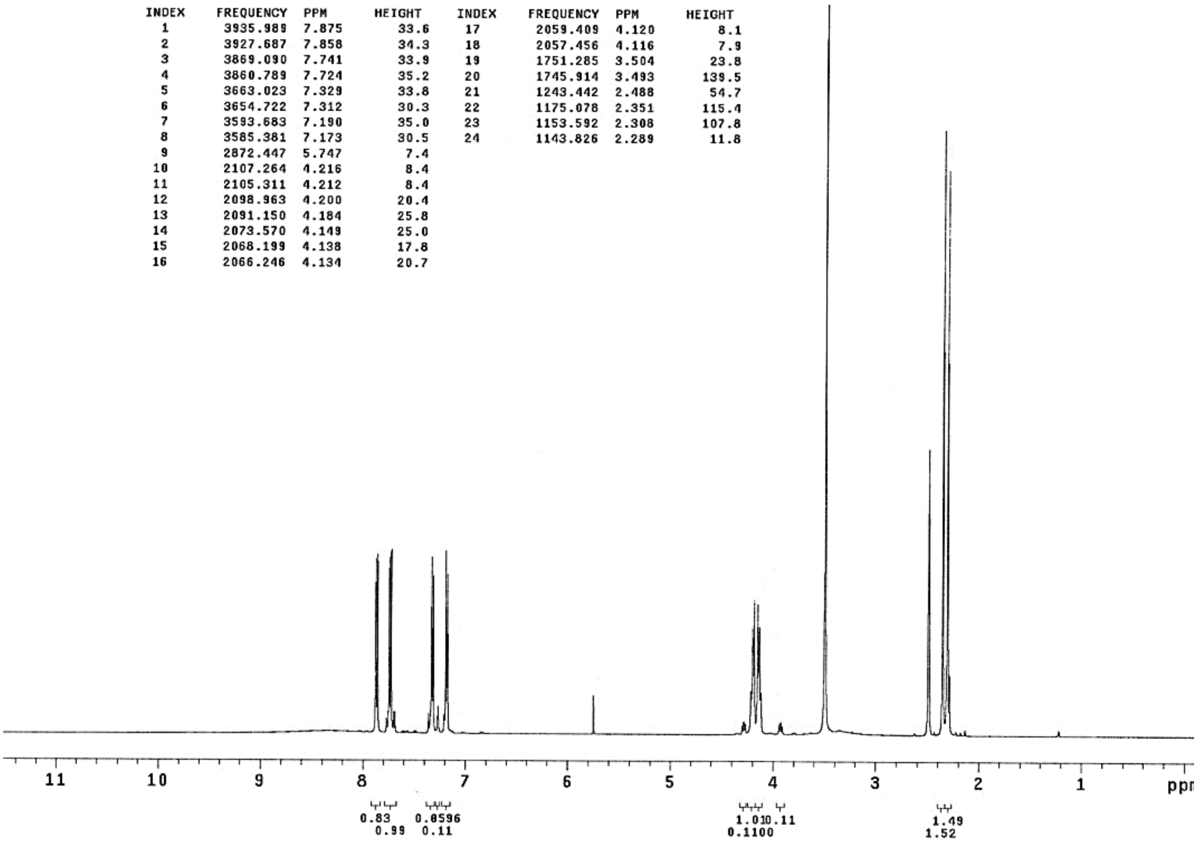

INDEX	FREQUENCY	PPM	HEIGHT
1	3935.989	7.875	33.6
2	3927.687	7.858	34.3
3	3869.090	7.741	33.9
4	3860.789	7.724	35.2
5	3663.023	7.329	33.8
6	3654.722	7.312	30.3
7	3593.683	7.190	35.0
8	3585.381	7.173	30.5
9	2872.447	5.747	7.4
10	2107.264	4.216	8.4
11	2105.311	4.212	8.4
12	2098.963	4.200	20.4
13	2091.150	4.184	25.8
14	2073.570	4.149	25.0
15	2068.199	4.138	17.8
16	2066.246	4.134	20.7
17	2059.409	4.120	8.1
18	2057.456	4.116	7.9
19	1751.285	3.504	23.8
20	1745.914	3.493	139.5
21	1243.442	2.488	54.7
22	1175.078	2.351	115.4
23	1153.592	2.308	107.8
24	1143.826	2.289	11.8

^{1}H-NMR (DMSO-d_6+TFA, 500 MHz) spectrum of *N*-((7-(4,5-dihydro-1*H*-imidazol-2-yl)-2-(*p*-tolyl)-6,7-dihydro-2*H*-imidazo[2,1-*c*][1,2,4]triazol-3(5*H*)-ylidene)carbamoyl)-4-methylbenzenesulfonamide (**6i**)

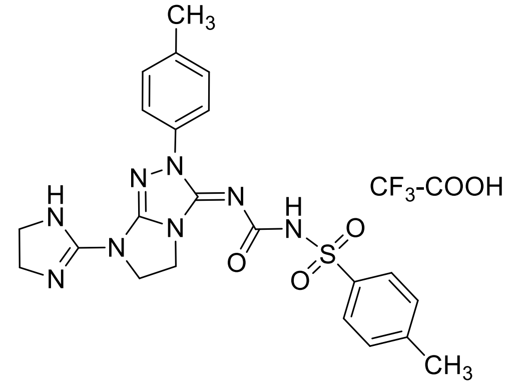

INDEX	FREQUENCY	PPM	HEIGHT	INDEX	FREQUENCY	PPM	HEIGHT	INDEX	FREQUENCY	PPM	HEIGHT
1	7728.419	15.463	4.1	17	2837.523	5.677	4.5	33	1165.414	2.332	83.8
2	3963.351	7.930	20.2	18	2288.190	4.578	1.6	34	1153.206	2.307	68.7
3	3954.805	7.913	21.0	19	2279.950	4.562	1.3				
4	3872.405	7.748	21.1	20	2192.056	4.386	4.4				
5	3864.165	7.731	22.4	21	2189.310	4.380	4.8				
6	3850.737	7.704	2.8	22	2182.901	4.367	12.2				
7	3842.497	7.688	2.9	23	2176.187	4.354	16.3				
8	3731.715	7.466	1.5	24	2165.505	4.333	15.4				
9	3723.475	7.450	2.6	25	2158.791	4.319	13.2				
10	3704.859	7.413	2.4	26	2152.382	4.306	5.9				
11	3696.619	7.396	1.4	27	2149.635	4.301	5.1				
12	3660.607	7.324	3.2	28	2143.532	4.289	1.4				
13	3653.893	7.311	22.9	29	1904.877	3.811	98.9				
14	3645.653	7.294	19.2	30	1243.846	2.489	40.0				
15	3605.368	7.213	20.4	31	1184.030	2.369	6.8				
16	3596.823	7.196	18.9	32	1177.926	2.357	1.4				

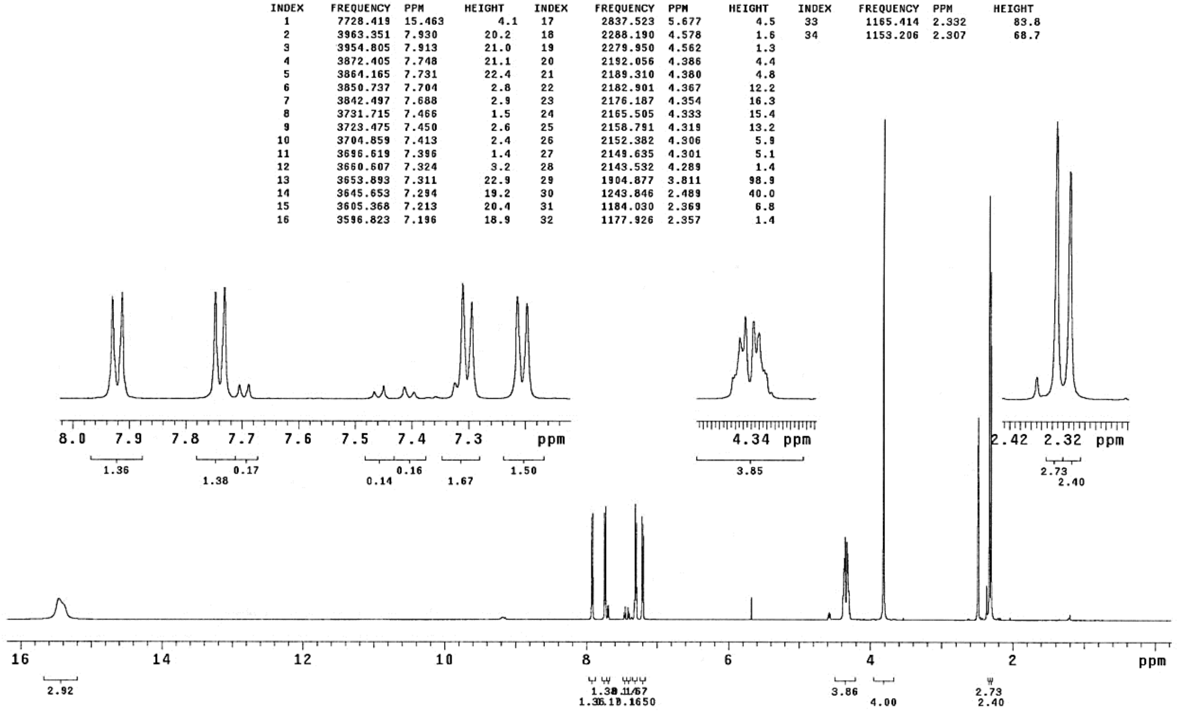

^{13}C-NMR (DMSO-d_6+TFA, 125 MHz) spectrum of *N*-((7-(4,5-dihydro-1*H*-imidazol-2-yl)-2-(*p*-tolyl)-6,7-dihydro-2*H*-imidazo[2,1-*c*][1,2,4]triazol-3(5*H*)-ylidene)carbamoyl)-4-methylbenzenesulfonamide (**6i**)

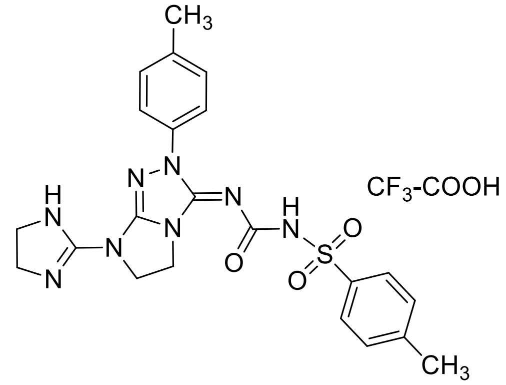

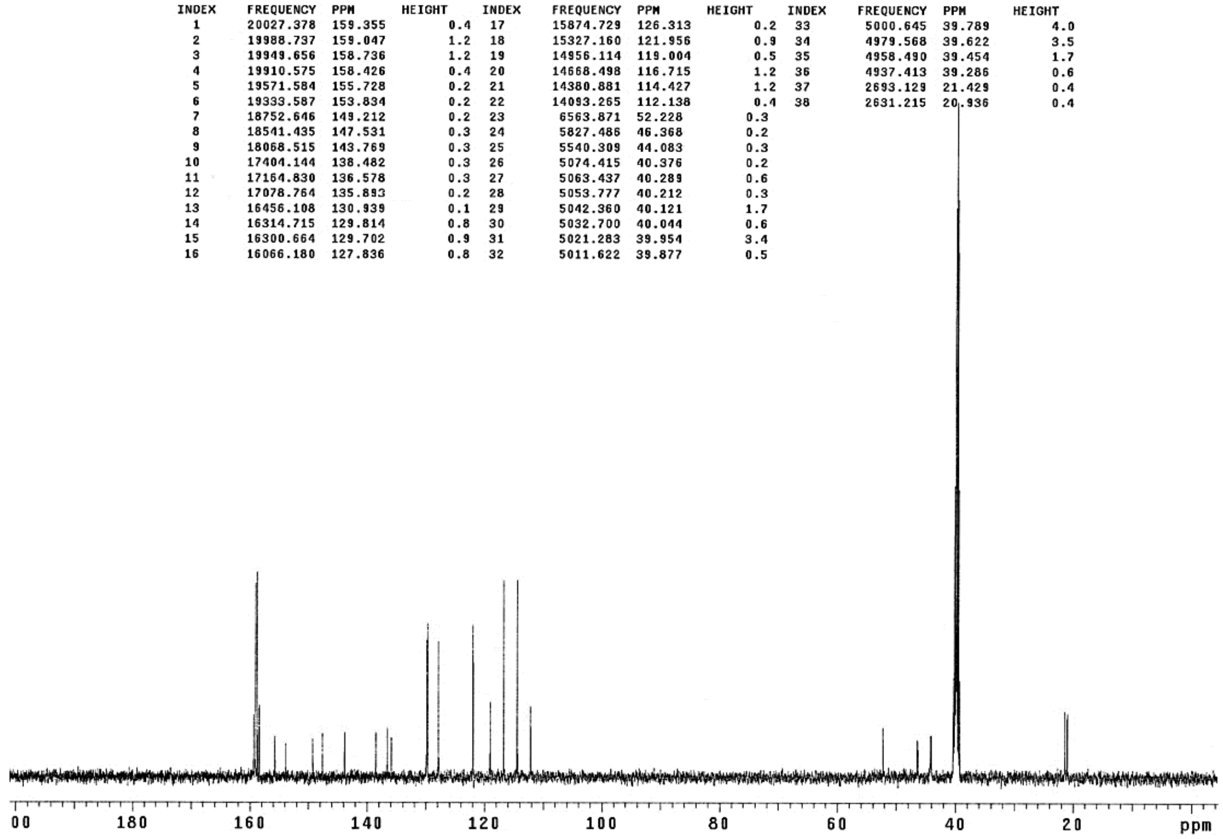

INDEX	FREQUENCY	PPM	HEIGHT	INDEX	FREQUENCY	PPM	HEIGHT	INDEX	FREQUENCY	PPM	HEIGHT
1	20027.378	159.355	0.4	17	15874.729	126.313	0.2	33	5000.645	39.789	4.0
2	19988.737	159.047	1.2	18	15327.160	121.956	0.9	34	4979.568	39.622	3.5
3	19949.656	158.736	1.2	19	14956.114	119.004	0.5	35	4958.490	39.454	1.7
4	19910.575	158.426	0.4	20	14668.498	116.715	1.2	36	4937.413	39.286	0.6
5	19571.584	155.728	0.2	21	14380.881	114.427	1.2	37	2693.129	21.429	0.4
6	19333.587	153.834	0.2	22	14093.265	112.138	0.4	38	2631.215	20.936	0.4
7	18752.646	149.212	0.2	23	6563.871	52.228	0.3				
8	18541.435	147.531	0.3	24	5827.486	46.368	0.2				
9	18068.515	143.769	0.3	25	5540.309	44.083	0.3				
10	17404.144	138.482	0.3	26	5074.415	40.376	0.2				
11	17164.830	136.578	0.3	27	5063.437	40.289	0.6				
12	17078.764	135.893	0.2	28	5053.777	40.212	0.3				
13	16456.108	130.939	0.1	29	5042.360	40.121	1.7				
14	16314.715	129.814	0.8	30	5032.700	40.044	0.6				
15	16300.664	129.702	0.9	31	5021.283	39.954	3.4				
16	16066.180	127.836	0.8	32	5011.622	39.877	0.5				

^{1}H-NMR (DMSO-d_6, 500 MHz) spectrum of 1-(7-(4,5-dihydro-1*H*-imidazol-2-yl)-2-phenyl-6,7-dihydro-2*H*-imidazo[2,1-*c*][1,2,4]triazol-3(5*H*)-ylidene)-3-phenylthiourea (**7a**)

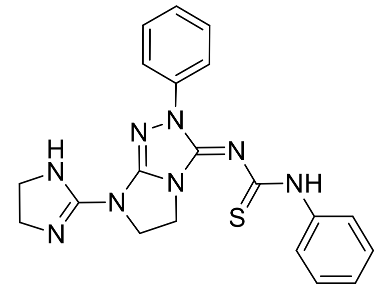

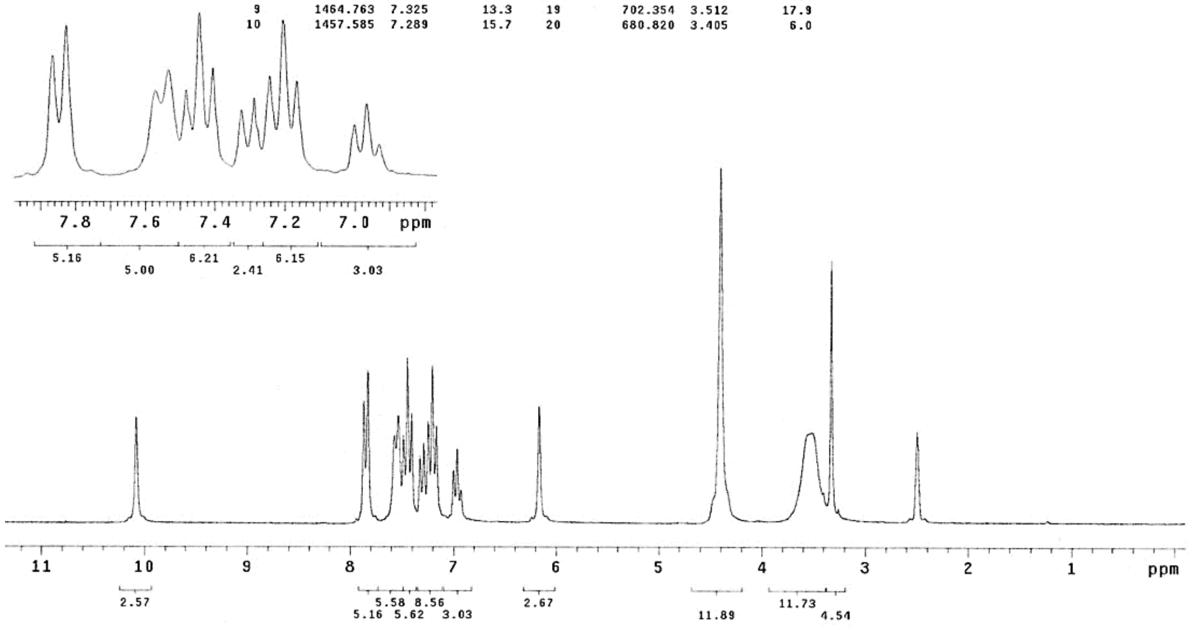

INDEX	FREQUENCY	PPM	HEIGHT	INDEX	FREQUENCY	PPM	HEIGHT	INDEX	FREQUENCY	PPM	HEIGHT
1	2016.190	10.083	20.8	11	1448.613	7.244	20.0	21	665.951	3.330	51.8
2	1573.203	7.867	24.0	12	1440.922	7.206	31.0	22	651.339	3.257	2.8
3	1565.256	7.828	30.0	13	1433.231	7.167	19.1	23	499.831	2.500	18.1
4	1514.241	7.573	17.2	14	1400.161	7.002	10.3				
5	1506.806	7.535	21.2	15	1392.983	6.966	14.5				
6	1496.552	7.484	17.3	16	1385.549	6.929	6.4				
7	1489.117	7.447	32.5	17	1233.528	6.169	23.0				
8	1481.170	7.407	21.6	18	881.036	4.406	70.4				
9	1464.763	7.325	13.3	19	702.354	3.512	17.9				
10	1457.585	7.289	15.7	20	680.820	3.405	6.0				

^{13}C-NMR (DMSO-d_6, 125 MHz) spectrum of 1-(7-(4,5-dihydro-1*H*-imidazol-2-yl)-2-phenyl-6,7-dihydro-2*H*-imidazo[2,1-*c*][1,2,4]triazol-3(5*H*)-ylidene)-3-phenylthiourea (**7a**) recorded at a temperature of 40°C

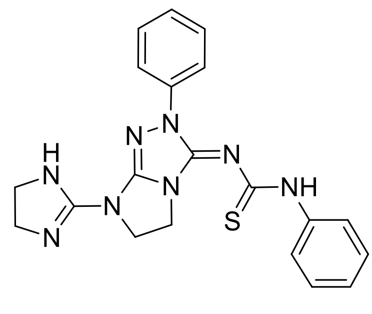

FREQUENCY	PPM	HEIGHT	15306.961	121.795	3.5
22815.278	181.538	0.4	14864.779	118.277	0.4
19503.961	155.190	3.4	6516.008	51.847	5.0
19071.439	151.749	2.8	6201.167	49.342	0.3
18672.289	148.573	0.5	5653.160	44.981	4.1
17737.866	141.138	3.1	5129.743	40.817	3.0
17365.063	138.171	2.3	5108.665	40.649	9.1
16282.221	129.555	14.9	5087.588	40.481	18.2
16193.082	128.846	12.1	5066.950	40.317	21.5
15995.484	127.274	3.8	5045.873	40.149	18.5
15531.785	123.584	3.6	5024.796	39.982	9.2
15318.817	121.890	15.5 ...	5003.718	39.814	3.0

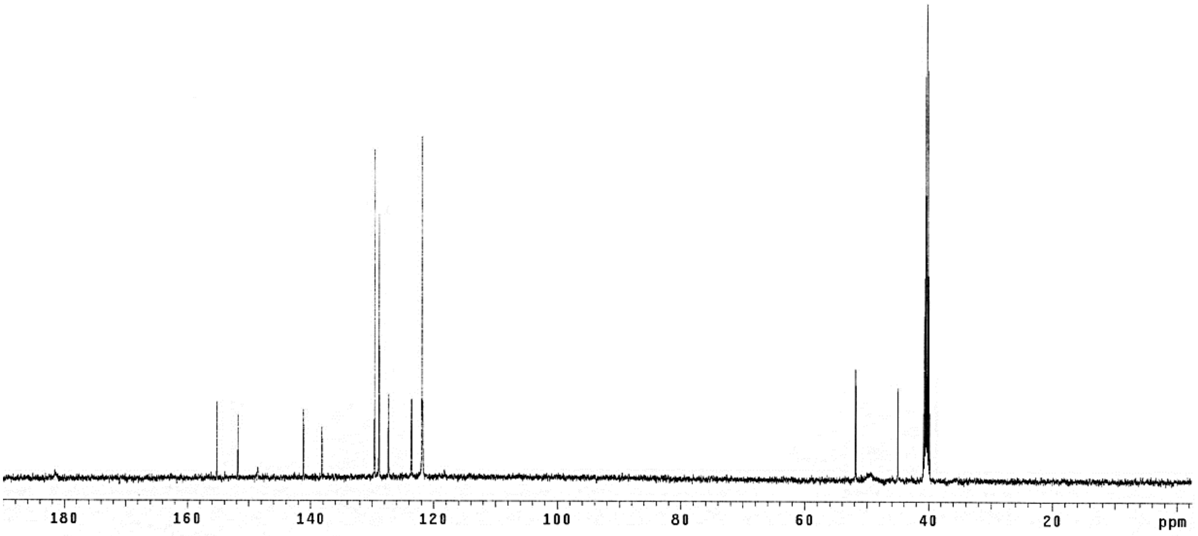

^{1}H-NMR ($CDCl_3$, 500 MHz) spectrum of 1-(7-(4,5-dihydro-1*H*-imidazol-2-yl)-2-phenyl-6,7-dihydro-2*H*-imidazo[2,1-*c*][1,2,4]triazol-3(5*H*)-ylidene)-3-(*p*-tolyl)thiourea (**7b**)

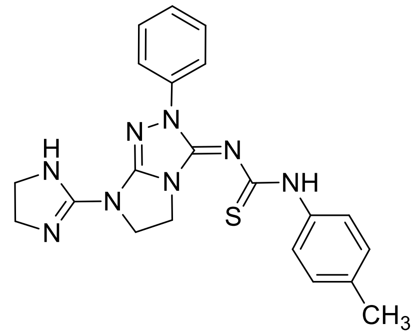

INDEX	FREQUENCY	PPM	HEIGHT
1	4045.849	8.095	5.1
2	3991.159	7.985	0.5
3	3881.289	7.766	10.0
4	3693.777	7.390	10.4
5	3644.458	7.292	7.6
6	3630.297	7.263	10.1
7	3507.731	7.018	8.3
8	3453.528	6.910	1.0
9	2751.337	5.505	0.9
10	2347.015	4.696	10.8
11	2255.213	4.512	11.7
12	2034.496	4.071	0.4
13	1879.213	3.760	5.8
14	1836.242	3.674	5.9
15	1133.074	2.267	21.2

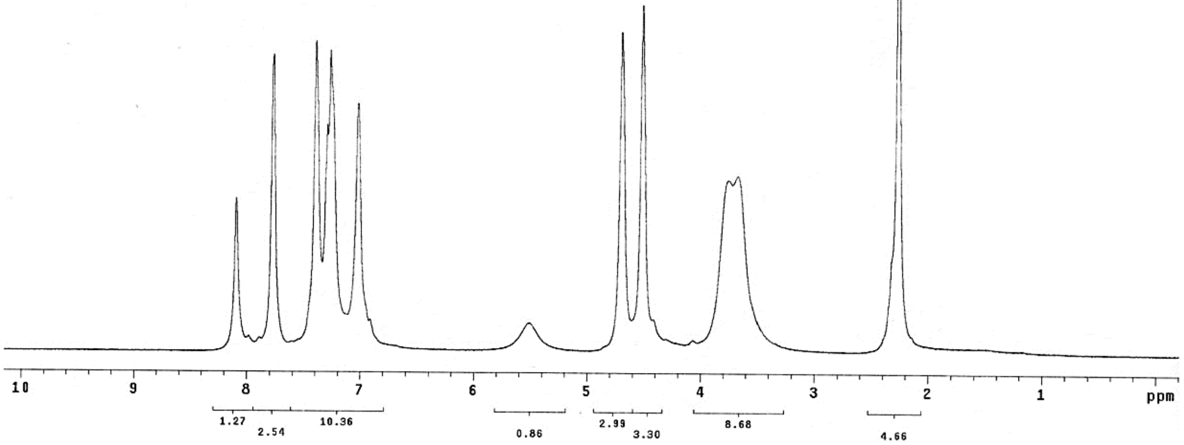

^{1}H-NMR (DMSO-d_6, 500 MHz) spectrum of 1-(4-chlorophenyl)-3-(7-(4,5-dihydro-1*H*-imidazol-2-yl)-2-phenyl-6,7-dihydro-2*H*-imidazo[2,1-*c*][1,2,4]triazol-3(5*H*)-ylidene)thiourea (**7c**)

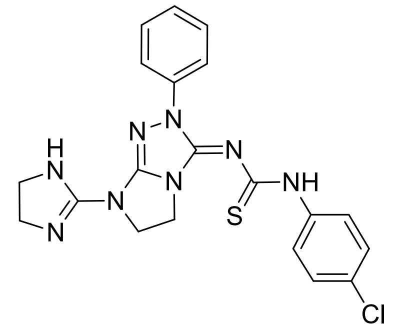

INDEX	FREQUENCY	PPM	HEIGHT	INDEX	FREQUENCY	PPM	HEIGHT
1	5091.333	10.186	24.1	17	2197.601	4.397	93.3
2	3910.597	7.824	38.4	18	2193.207	4.388	95.0
3	3902.784	7.809	40.1	19	2181.487	4.365	18.7
4	3788.030	7.579	15.3	20	2176.604	4.355	9.5
5	3730.898	7.465	43.0	21	1762.028	3.525	188.7
6	3723.085	7.449	74.5	22	1670.225	3.342	20.3
7	3715.272	7.433	45.6	23	1244.907	2.491	18.6
8	3654.722	7.312	26.8	24	1243.442	2.488	24.3
9	3647.397	7.298	45.1	25	1241.977	2.485	18.4
10	3640.072	7.283	19.3				
11	3622.981	7.249	52.8				
12	3614.192	7.231	46.5				
13	3126.857	6.256	8.1				
14	2213.716	4.429	10.0				
15	2209.809	4.421	18.3				
16	2201.020	4.404	55.0				

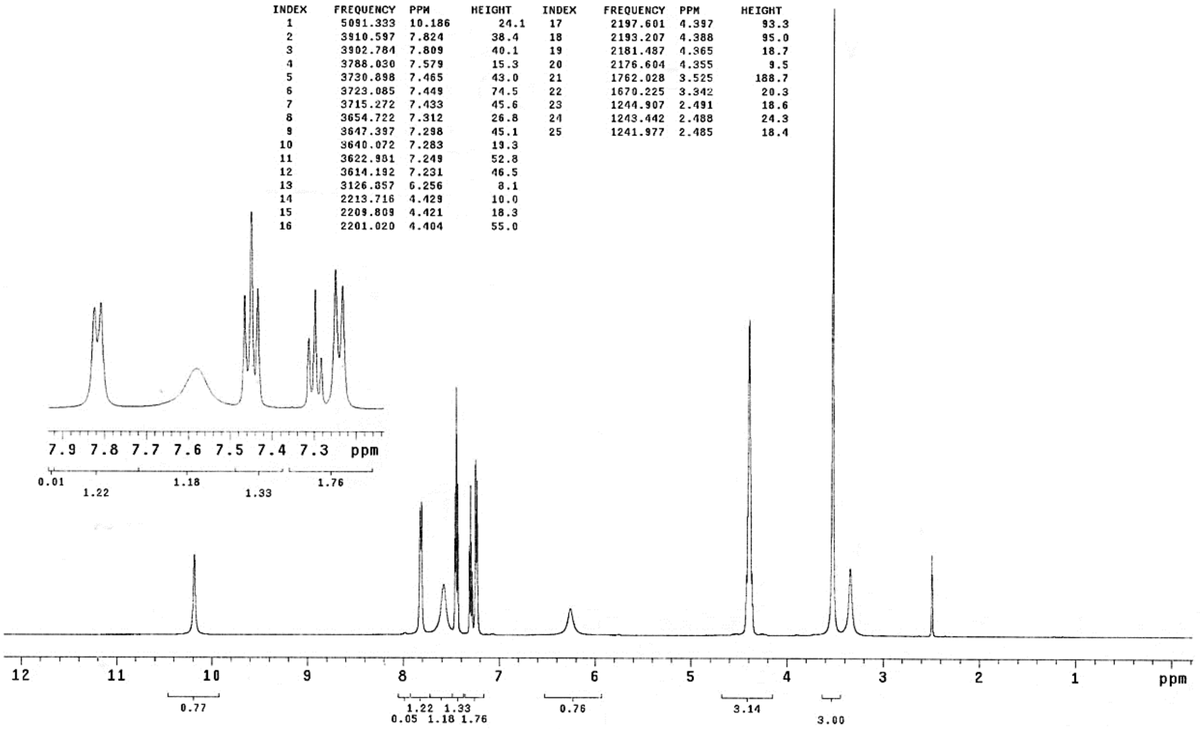

^{1}H-NMR (DMSO-d_6, 500 MHz) spectrum of 1-(7-(4,5-dihydro-1*H*-imidazol-2-yl)-2-phenyl-6,7-dihydro-2*H*-imidazo[2,1-*c*][1,2,4]triazol-3(5*H*)-ylidene)-3-(4-nitrophenyl)thiourea (**7d**)

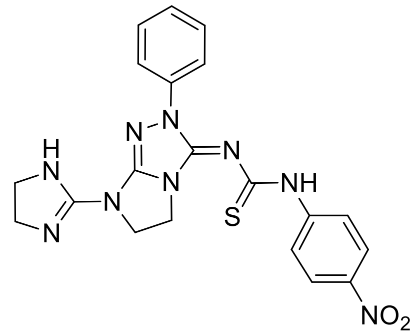

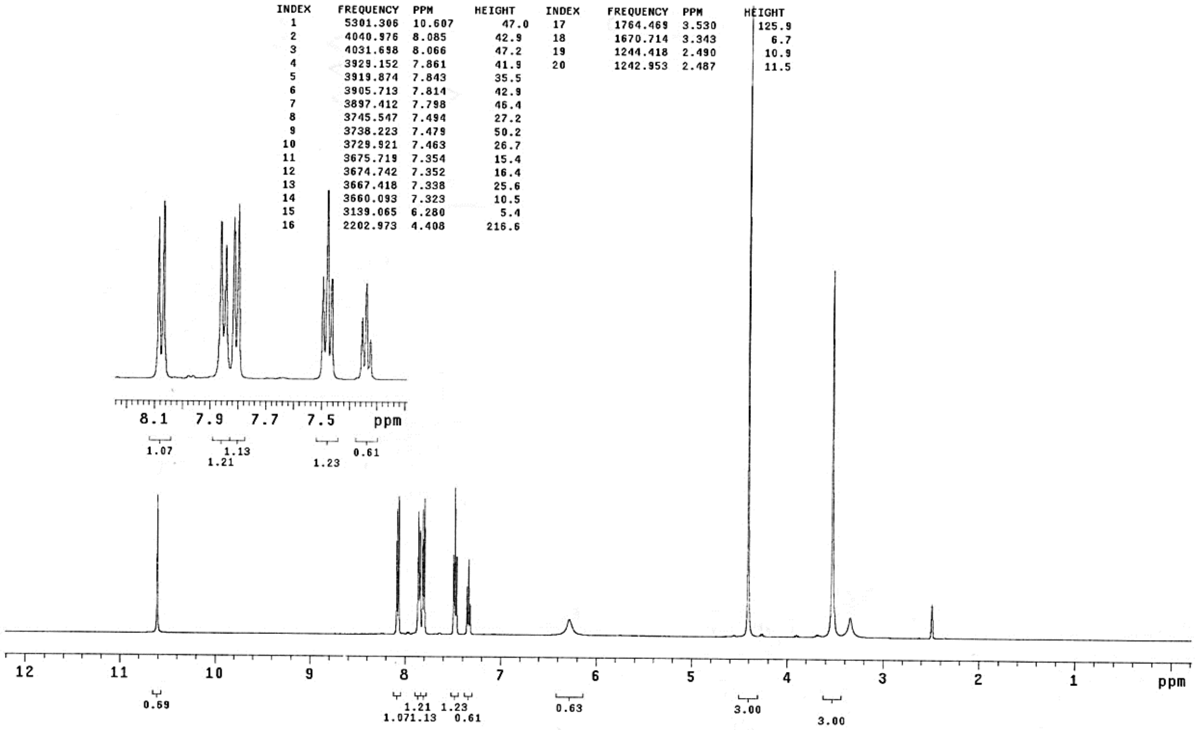

^{1}H-NMR (DMSO-d_6+TFA, 500 MHz) spectrum of 1-(7-(4,5-dihydro-1*H*-imidazol-2-yl)-2-phenyl-6,7-dihydro-2*H*-imidazo[2,1-*c*][1,2,4]triazol-3(5*H*)-ylidene)-3-(4-nitrophenyl)thiourea (**7d**)

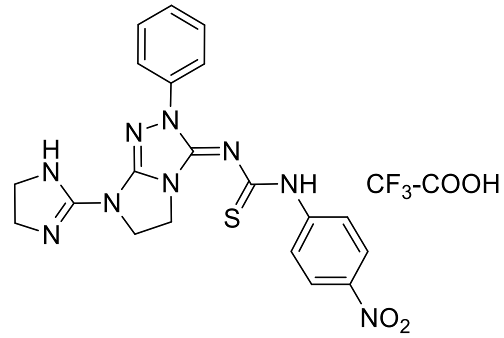

INDEX	FREQUENCY	PPM	HEIGHT
1	5344.766	10.694	16.9
2	4632.320	9.268	7.9
3	4019.978	8.043	21.5
4	4010.700	8.024	26.5
5	3928.664	7.860	21.8
6	3920.851	7.845	33.9
7	3912.062	7.827	11.6
8	3733.340	7.469	12.8
9	3725.527	7.454	23.0
10	3717.225	7.437	15.4
11	3667.418	7.338	8.1
12	3659.605	7.322	13.0
13	3652.280	7.307	5.5
14	2279.149	4.560	91.7
15	1917.799	3.837	93.1
16	1243.442	2.488	12.2
17	1241.977	2.485	9.8
18	1003.681	2.008	30.6

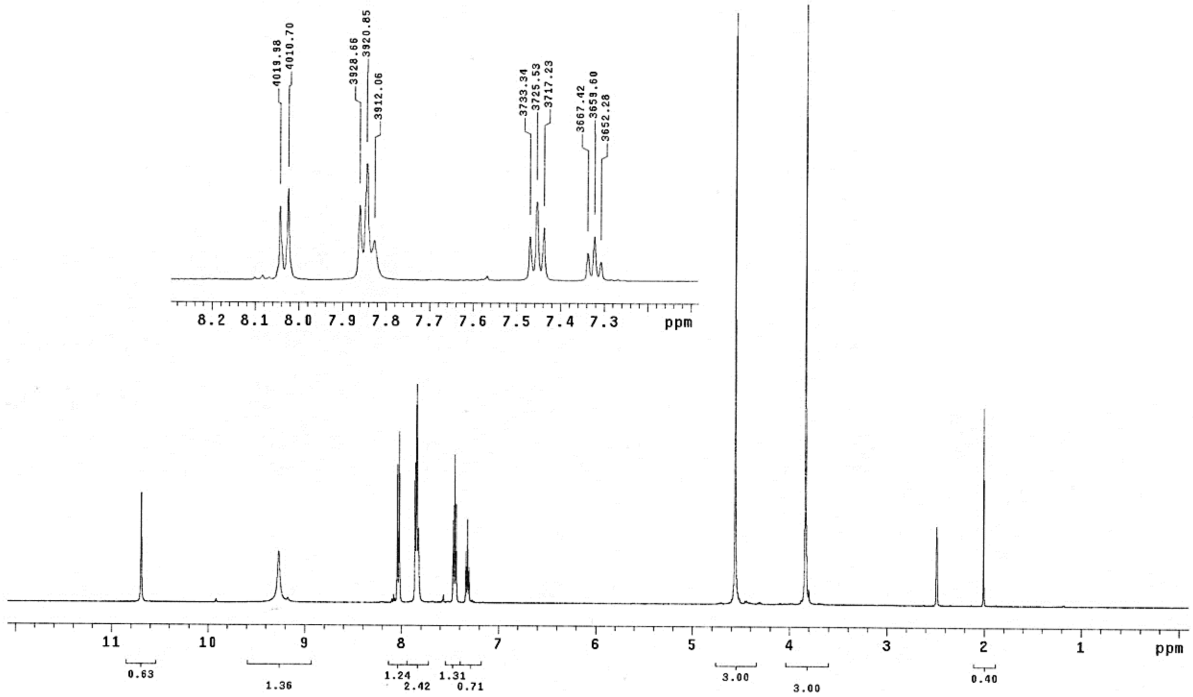

^{13}C-NMR (DMSO-d_6+TFA, 125 MHz) spectrum of 1-(7-(4,5-dihydro-1*H*-imidazol-2-yl)-2-phenyl-6,7-dihydro-2*H*-imidazo[2,1-*c*][1,2,4]triazol-3(5*H*)-ylidene)-3-(4-nitrophenyl)thiourea (**7d**)

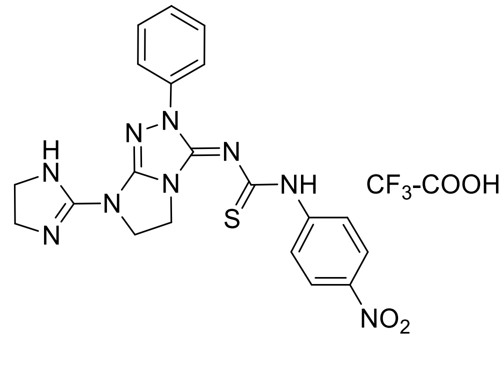

INDEX	FREQUENCY	PPM	HEIGHT	INDEX	FREQUENCY	PPM	HEIGHT	INDEX	FREQUENCY	PPM	HEIGHT
1	22883.779	182.083	0.1	11	17269.777	137.413	0.2	21	6667.501	53.052	0.2
2	20053.286	159.561	0.6	12	16294.956	129.657	1.0	22	5752.399	45.771	0.2
3	20014.644	159.254	2.0	13	16120.630	128.269	0.3	23	5560.947	44.248	0.3
4	19975.564	158.943	2.1	14	15698.207	124.908	0.6	24	5022.161	39.961	0.4
5	19936.483	158.632	0.7	15	15409.713	122.613	1.0	25	5001.084	39.793	1.3
6	19371.789	154.138	0.3	16	15103.215	120.174	0.5	26	4980.007	39.625	2.5
7	18889.648	150.302	0.2	17	14957.870	119.018	0.6	27	4958.930	39.457	3.0
8	18703.466	148.821	0.1	18	14670.254	116.729	1.5	28	4937.852	39.290	2.5
9	18447.466	146.784	0.2	19	14382.638	114.441	1.5	29	4916.775	39.122	1.3
10	17859.060	142.102	0.2	20	14095.022	112.152	0.6	30	4895.698	38.954	0.4

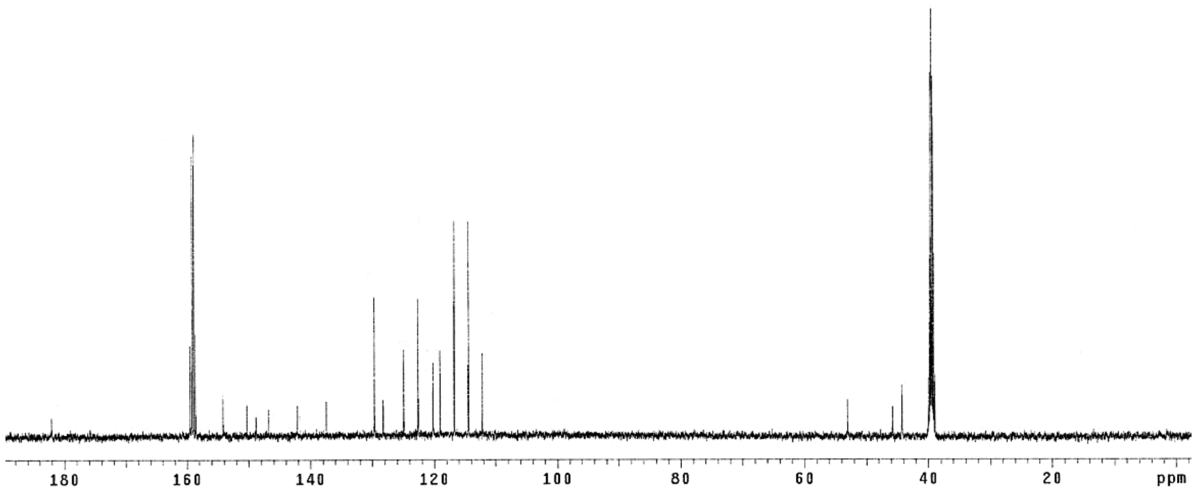

^{1}H-NMR ($CDCl_3$, 200 MHz) spectrum of 4-methyl-*N*-(7-(1-(4-methylbenzoyl)-4,5-dihydro-1*H*-imidazol-2-yl)-2-(*p*-tolyl)-6,7-dihydro-2*H*-imidazo[2,1-*c*][1,2,4]triazol-3(5*H*)-ylidene)benzamide (**8a**)

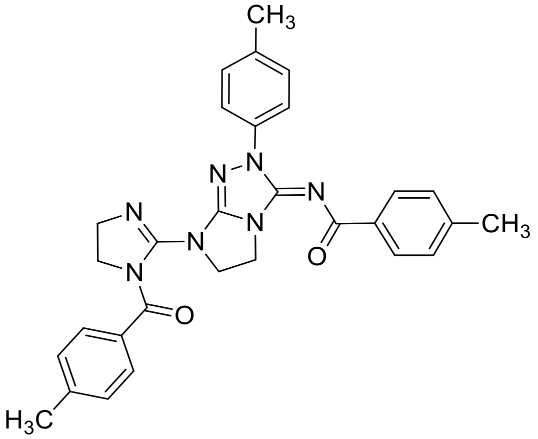

FREQ	PPM	INTENSITY
1619.85	8.100	36.094
1611.78	8.060	39.722
1545.43	7.728	35.234
1537.26	7.687	74.333
1528.67	7.644	40.977
1460.47	7.303	39.940
1452.86	7.265	47.217
1444.18	7.222	39.458
1436.16	7.182	36.037
1413.83	7.070	37.703
1405.48	7.028	33.299
907.10	4.536	124.905
812.78	4.064	11.834
804.84	4.025	31.995
797.47	3.988	24.702
769.81	3.850	24.775
762.61	3.814	32.815
754.67	3.774	11.983
490.02	2.450	122.338
478.18	2.391	121.821
460.73	2.304	119.958

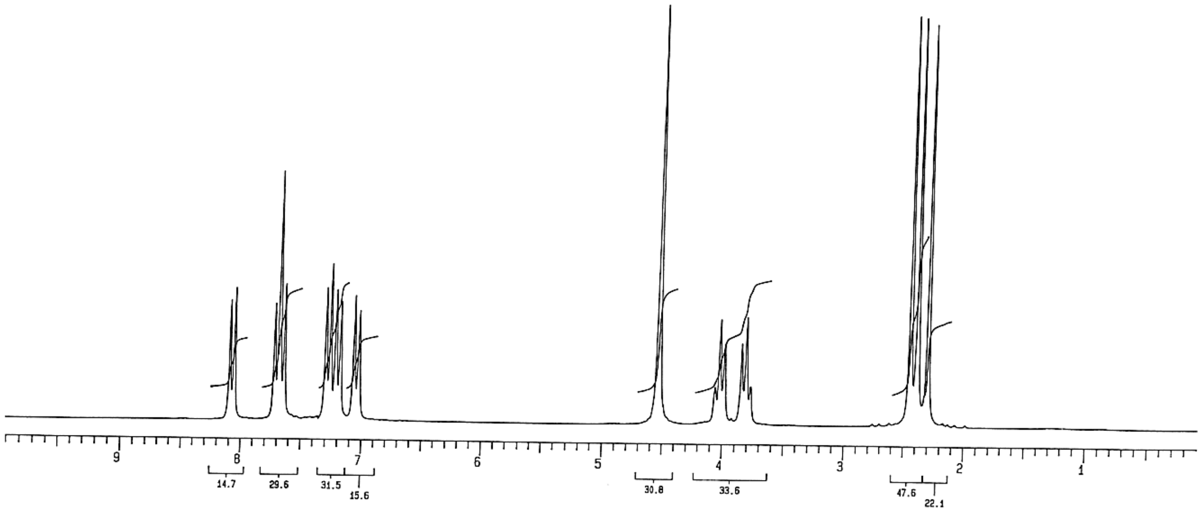

^{13}C-NMR ($CDCl_3$, 50 MHz) spectrum of 4-methyl-*N*-(7-(1-(4-methylbenzoyl)-4,5-dihydro-1*H*-imidazol-2-yl)-2-(*p*-tolyl)-6,7-dihydro-2*H*-imidazo[2,1-*c*][1,2,4]triazol-3(5*H*)-ylidene)benzamide (**8a**)

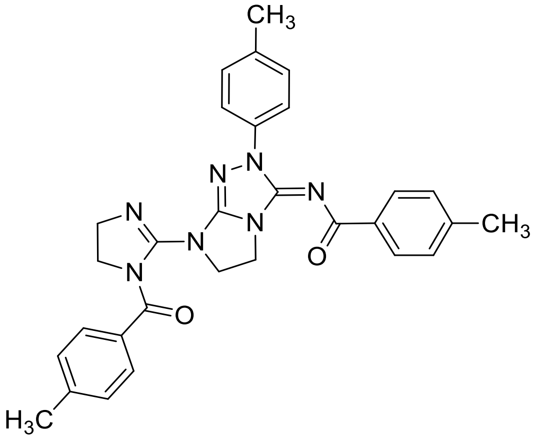

FREQ	PPM	INTENSITY
8682.4	172.650	17.496
8622.8	171.463	14.014
7605.5	151.236	18.546
7530.6	149.745	14.732
7449.1	148.124	12.185
7239.4	143.955	35.797
7138.8	141.955	32.399
6858.7	136.385	24.772
6844.1	136.093	33.641
6803.6	135.289	21.005
6630.8	131.853	29.167
6538.6	130.020	114.551
6520.7	129.664	115.825
6516.4	129.579	121.312
6508.3	129.417	131.361
6493.4	129.120	117.676
6119.3	121.681	125.964
3930.4	78.156	121.881
3898.5	77.521	124.299
3866.5	76.886	121.786
2650.4	52.704	39.840
2646.4	52.623	46.815
2619.7	52.093	44.463
2285.7	45.451	43.889
1517.3	30.172	9.725
1115.2	22.175	43.075
1107.1	22.015	47.913
1077.5	21.427	39.584

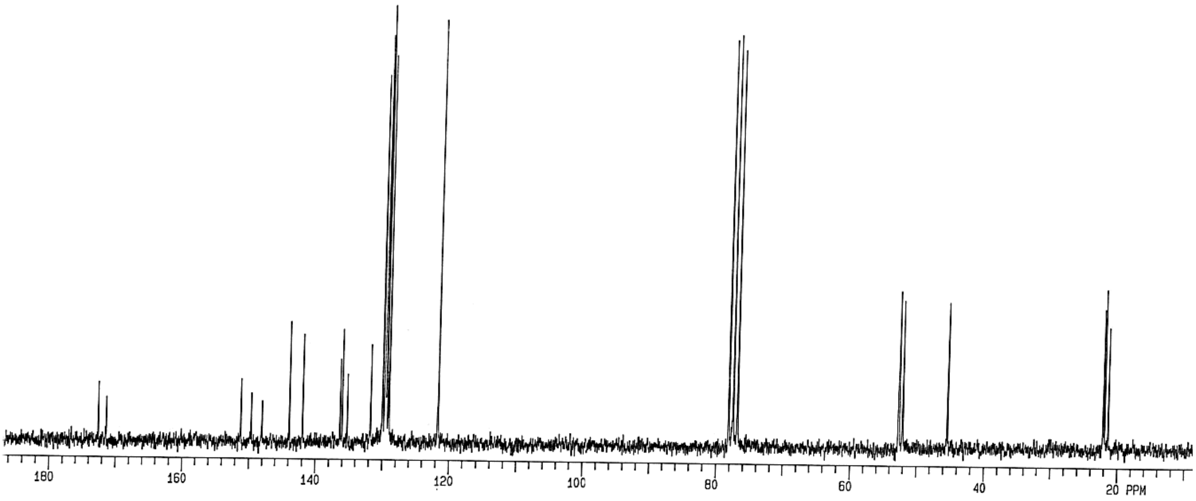

^{1}H-NMR ($CDCl_3$, 200 MHz) spectrum of 4-methoxy-*N*-(7-(1-(4-methoxybenzoyl)-4,5-dihydro-1*H*-imidazol-2-yl)-2-(*p*-tolyl)-6,7-dihydro-2*H*-imidazo[2,1-*c*][1,2,4]triazol-3(5*H*)-ylidene)benzamide (**8b**)

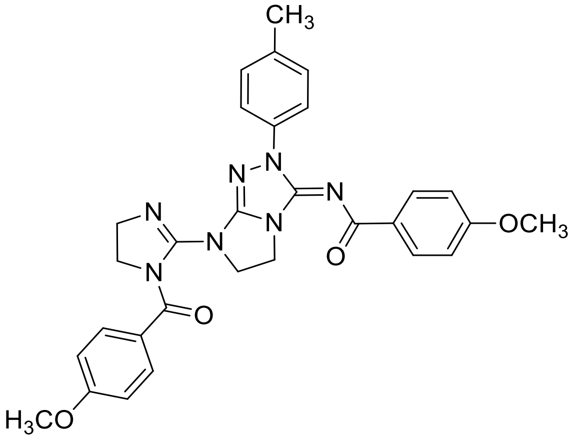

FREQ	PPM	INTENSITY
1631.69	8.159	24.378
1629.50	8.149	7.408
1622.90	8.116	20.560
1564.13	7.822	22.570
1562.10	7.811	7.674
1555.30	7.777	22.704
1530.68	7.654	20.489
1522.17	7.612	20.743
1453.51	7.268	6.849
1452.21	7.262	11.457
1412.29	7.062	22.066
1403.86	7.020	19.814
1398.37	6.993	26.191
1389.58	6.949	20.582
1382.25	6.912	26.708
1380.05	6.901	8.146
1373.46	6.868	20.271
907.53	4.538	58.354
814.78	4.074	6.877
806.52	4.033	17.203
799.11	3.996	13.219
778.03	3.891	90.140
767.98	3.840	98.215
762.48	3.813	20.186
754.79	3.774	7.246
459.19	2.296	65.005

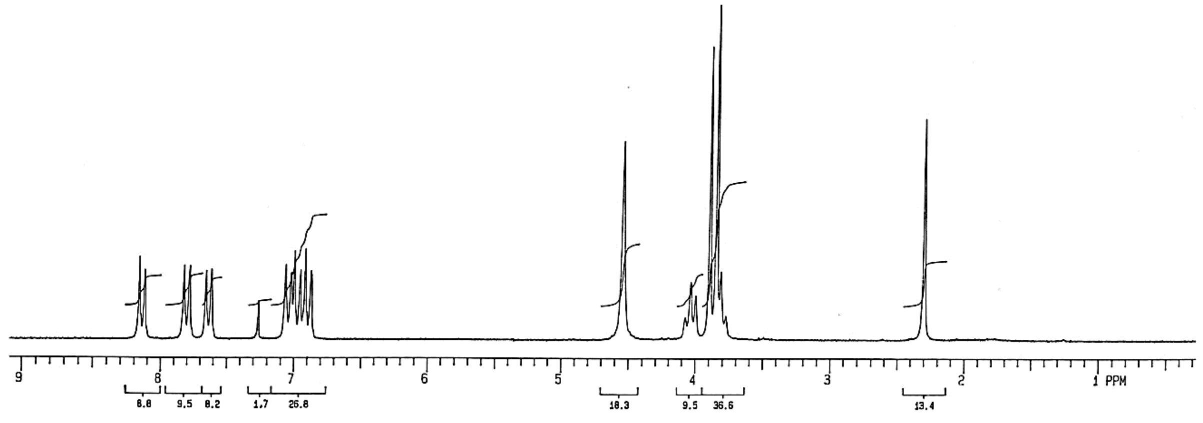

^{13}C-NMR ($CDCl_3$, 50 MHz) spectrum of 4-methoxy-*N*-(7-(1-(4-methoxybenzoyl)-4,5-dihydro-1*H*-imidazol-2-yl)-2-(*p*-tolyl)-6,7-dihydro-2*H*-imidazo[2,1-*c*][1,2,4]triazol-3(5*H*)-ylidene)benzamide (**8b**)

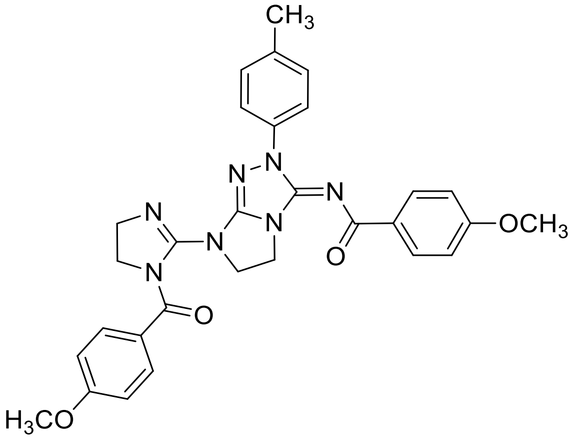

FREQ	PPM	INTENSITY			
8661.7	172.237	7.783	5745.0	114.239	10.620
8606.6	171.141	12.368	5712.8	113.598	85.142
8236.1	163.773	19.575	3947.0	78.485	4.348
8180.1	162.661	17.716	3929.3	78.133	394.366
7623.5	151.593	12.545	3907.4	77.699	20.787
7524.8	149.630	11.205	3897.4	77.498	408.127
7440.4	147.952	8.486	3865.3	76.862	400.819
6859.0	136.391	14.865	3853.4	76.625	4.864
6844.0	136.091	21.212	2819.4	56.064	37.918
6631.7	131.872	99.526	2807.6	55.830	39.425
6629.9	131.835	92.480	2654.0	52.775	28.176
6568.9	130.622	14.111	2639.4	52.483	21.771
6508.4	129.419	88.341	2622.6	52.150	29.427
6368.5	126.637	17.938	2287.6	45.490	31.094
6117.8	121.651	80.302	2287.6	45.490	31.094
5749.1	114.319	78.045	1077.1	21.419	30.647

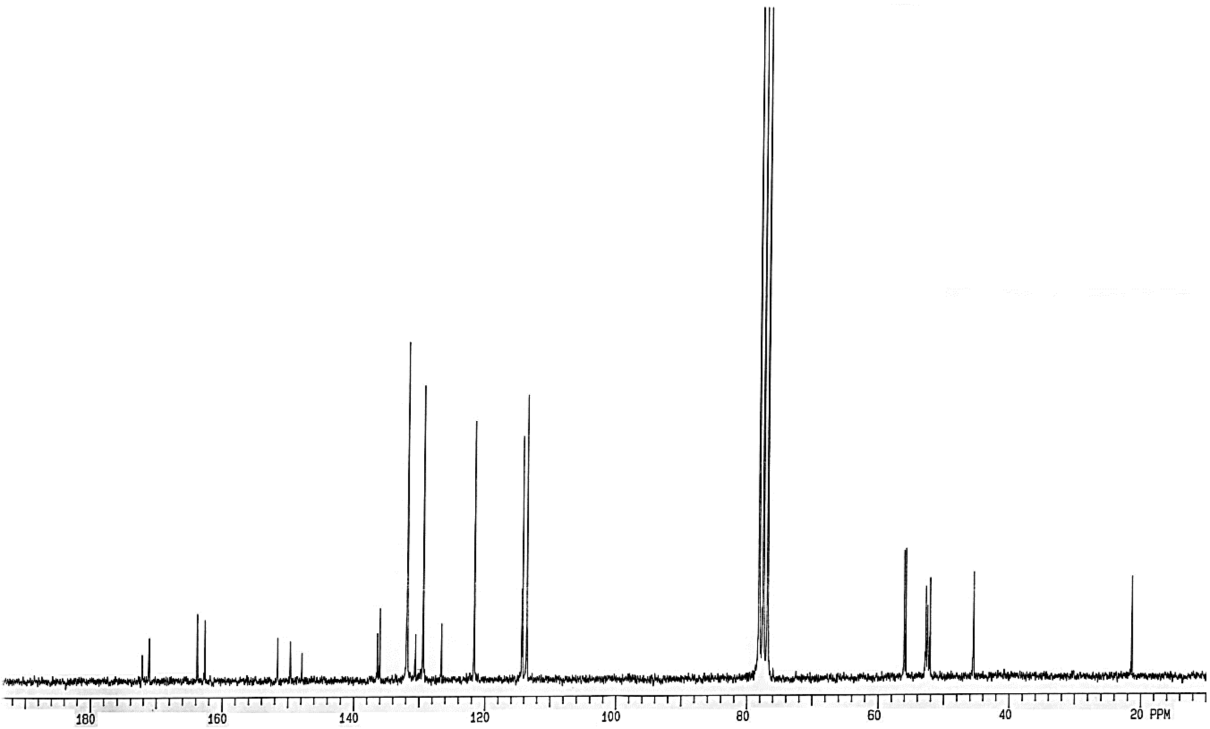

^{1}H-NMR (DMSO-d_6, 500 MHz) spectrum of 4-chloro-*N*-(7-(1-(4-chlorobenzoyl)-4,5-dihydro-1*H*-imidazol-2-yl)-2-phenyl-6,7-dihydro-2*H*-imidazo[2,1-*c*][1,2,4]triazol-3(5*H*)-ylidene)benzamide(**8c**)

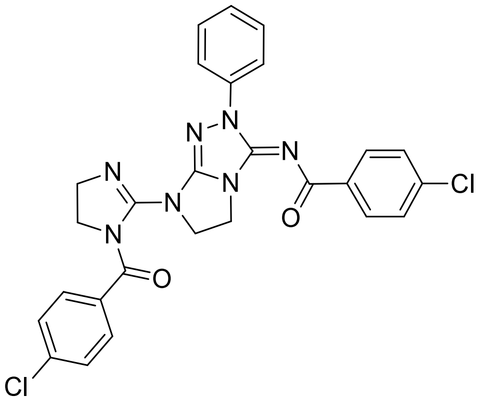

FREQUENCY	PPM	HEIGHT			
			3602.961	7.209	41.4
4027.303	8.058	198.9	2224.947	4.452	66.5
4018.513	8.040	216.7	2217.622	4.437	148.9
3890.088	7.783	123.7	2209.809	4.421	126.1
3881.786	7.766	160.6	2160.490	4.323	125.4
3863.719	7.730	169.1	1994.952	3.991	79.7
3855.417	7.714	180.6	1987.139	3.976	174.1
3803.168	7.609	176.6	1979.326	3.960	106.3
3794.867	7.593	159.2	1859.690	3.721	110.7
3748.966	7.501	216.2	1851.877	3.705	206.1
3740.176	7.483	205.8	1844.064	3.690	98.9
3684.020	7.371	87.1	1669.737	3.341	81372.7
3675.719	7.354	169.4	1658.018	3.317	1651.5
3667.906	7.339	111.3	1245.883	2.493	1575.2
3617.610	7.238	57.0	1243.930	2.489	2534.9
3610.285	7.223	96.7	1242.465	2.486	2380.0
			1240.512	2.482	1779.7

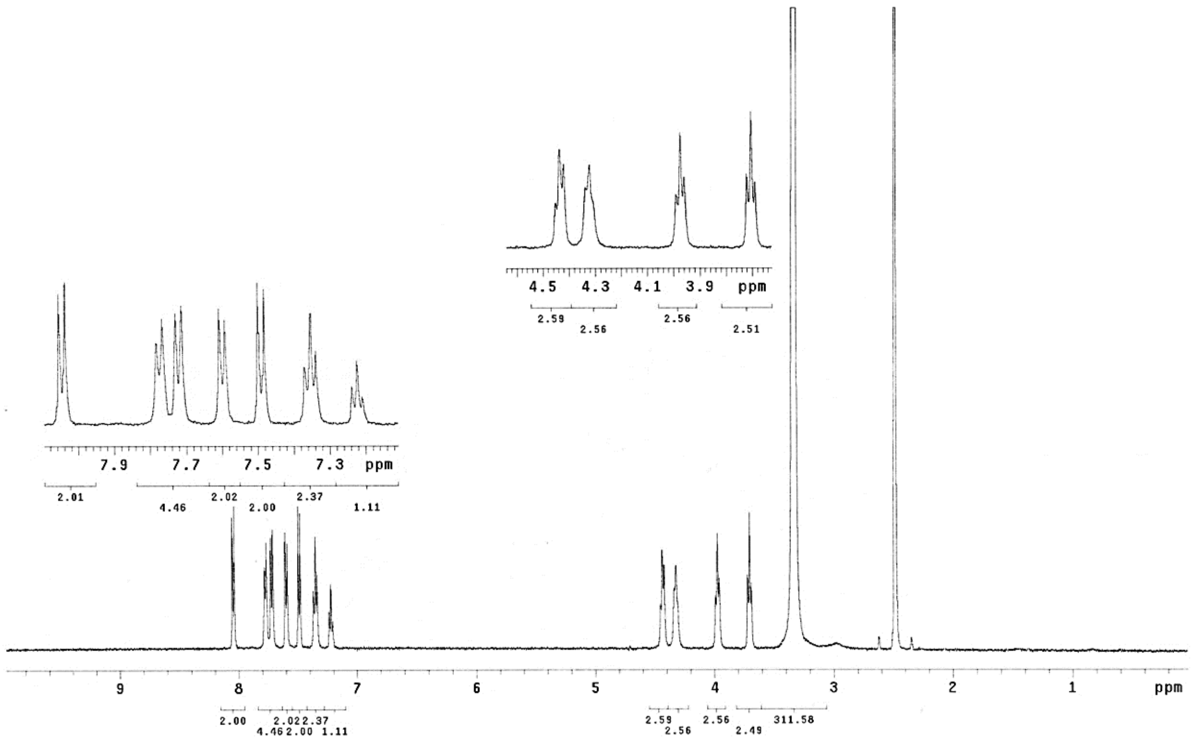

^{1}H-NMR ($CDCl_3$, 200 MHz) spectrum of *N*-(2-phenyl-7-(1-(phenylsulfonyl)-4,5-dihydro-1*H*-imidazol-2-yl)-6,7-dihydro-2*H*-imidazo[2,1-*c*][1,2,4]triazol-3(5*H*)-ylidene)benzenesulfonamide (**9**)

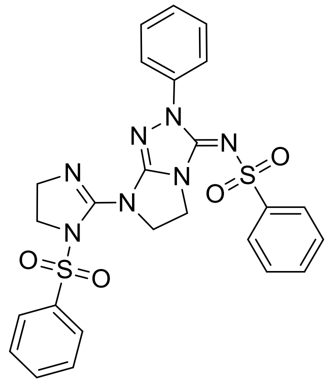

INDEX	FREQUENCY	PPM	HEIGHT	INDEX	FREQUENCY	PPM	HEIGHT	INDEX	FREQUENCY	PPM	HEIGHT
1	1600.277	8.003	31.6	11	1494.423	7.473	32.6	21	929.867	4.650	27.5
2	1594.466	7.974	59.1	12	1476.573	7.384	18.7	22	791.219	3.957	17.6
3	1587.409	7.938	41.2	13	1469.101	7.347	35.2	23	783.747	3.919	36.8
4	1550.464	7.754	32.9	14	1461.214	7.307	25.9	24	775.860	3.880	22.6
5	1542.992	7.716	43.7	15	1453.327	7.268	24.3	25	699.064	3.496	2.8
6	1535.520	7.679	21.9	16	1445.855	7.231	15.5	26	683.289	3.417	22.5
7	1528.048	7.642	19.8	17	1438.798	7.195	5.7	27	675.402	3.378	37.1
8	1515.594	7.579	33.1	18	954.774	4.775	27.6	28	667.930	3.340	17.6
9	1508.122	7.542	46.1	19	948.132	4.742	30.7				
10	1501.065	7.507	65.5	20	936.509	4.683	31.1				

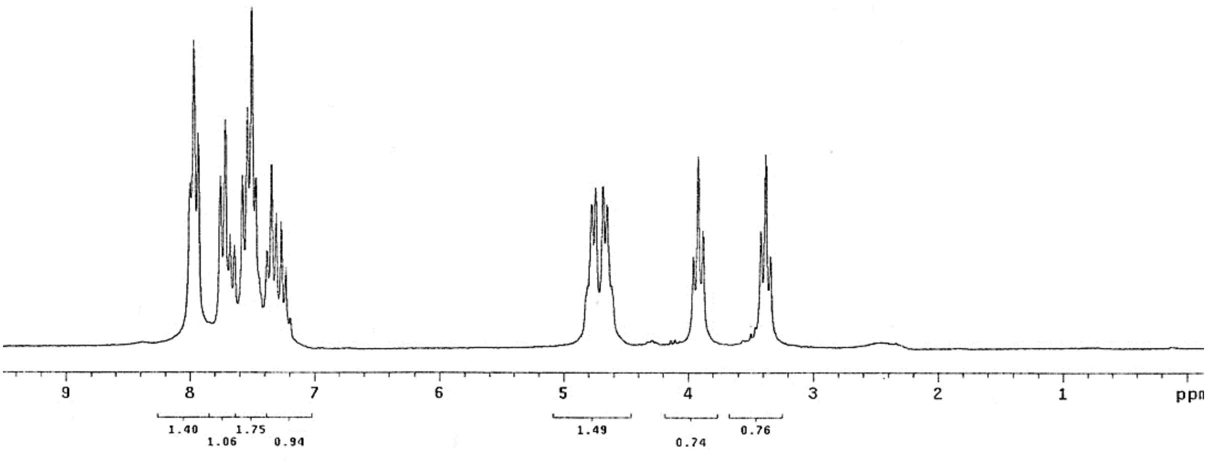

^{13}C-NMR ($CDCl_3$, 50 MHz) spectrum of *N*-(2-phenyl-7-(1-(phenylsulfonyl)-4,5-dihydro-1*H*-imidazol-2-yl)-6,7-dihydro-2*H*-imidazo[2,1-*c*][1,2,4]triazol-3(5*H*)-ylidene)benzenesulfonamide (**9**)

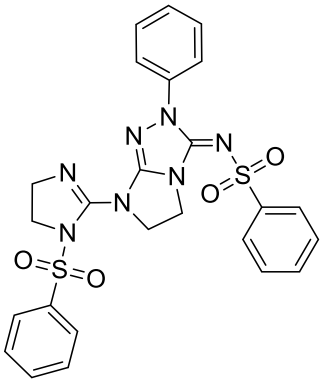

INDEX	FREQUENCY	PPM	HEIGHT	INDEX	FREQUENCY	PPM	HEIGHT	INDEX	FREQUENCY	PPM	HEIGHT
1	7546.923	150.095	4.9	11	6418.011	127.643	40.1	21	2267.493	45.096	16.1
2	7496.875	149.099	6.5	12	6374.490	126.777	19.7				
3	7222.263	143.638	3.6	13	6337.933	126.050	35.9				
4	7203.114	143.257	4.0	14	6146.010	122.233	38.8				
5	6905.871	137.345	6.0	15	3904.722	77.658	27.5				
6	6891.945	137.068	7.7	16	3872.952	77.026	27.9				
7	6750.940	134.264	19.3	17	3840.747	76.385	26.6				
8	6622.120	131.702	19.9	18	2694.426	53.587	16.2				
9	6516.366	129.599	39.5	19	2609.126	51.891	16.0				
10	6466.753	128.612	65.8	20	2522.521	50.168	18.0				

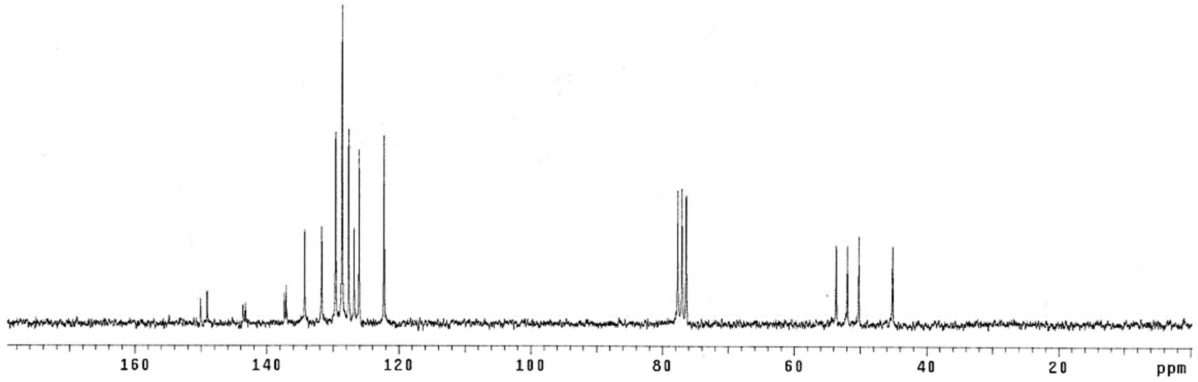

Supplement: Supplementary file 1 [file molecules-25-05924-s001.pdf]
